# The MTH1 inhibitor TH588 demonstrates anti-tumoral effects alone and in combination with everolimus, 5-FU and gamma-irradiation in neuroendocrine tumor cells

**DOI:** 10.1371/journal.pone.0178375

**Published:** 2017-05-25

**Authors:** Elke Tatjana Aristizabal Prada, Michael Orth, Svenja Nölting, Gerald Spöttl, Julian Maurer, Christoph Auernhammer

**Affiliations:** 1 Department of Internal Medicine II, Campus Grosshadern, Interdisciplinary Center of Neuroendocrine Tumours of the GastroEnteroPancreatic System (GEPNET-KUM), University-Hospital, Ludwig-Maximilians-University of Munich, Bavaria, Germany; 2 Department of Radiation Oncology, Campus Grosshadern, Ludwig-Maximilians-University Munich, Bavaria, Germany; Hunter College, UNITED STATES

## Abstract

Modulation of the redox system in cancer cells has been considered a promising target for anti-cancer therapy. The novel MTH1 inhibitor TH588 proved tremendous potential in terms of cancer cell eradication, yet its specificity has been questioned by recent reports, indicating that TH588 may also induce cancer cell death by alternative mechanisms than MTH1 inhibition. Here we used a panel of heterogeneous neuroendocrine tumor cells in order to assess cellular mechanisms and molecular signaling pathways implicated in the effects of TH588 alone as well as dual-targeting approaches combining TH588 with everolimus, cytotoxic 5-fluorouracil or γ-irradiation. Our results reflect that TH588 alone efficiently decreased the survival of neuroendocrine cancer cells by PI3K-Akt-mTOR axis downregulation, increased apoptosis and oxidative stress. However, in the dual-targeting approaches cell survival was further decreased due to an even stronger downregulation of the PI3K-Akt-mTOR axis and augmentation of apoptosis but not oxidative stress. Furthermore, we could attribute TH588 chemo- and radio-sensitizing properties. Collectively our data not only provide insights into how TH588 exactly kills cancer cells but also depict novel perspectives for combinatorial treatment approaches encompassing TH588.

## Introduction

Deregulation of the cellular redox system culminating in increased levels of oxidative stress is a common feature in cancer cells, and increased levels of oxidative stress are frequently associated with oncogenic transformation, deregulation of cell survival as well as proliferation, invasion and angiogenesis [1–3]. However, reactive oxygen species (ROS), which are highly abundant under oxidative stress conditions, can also compromise integrity of DNA thereby leading to proliferative arrest, senescence and even cell death [4, 5]. Thus, modulating the redox regulatory systems of cancer cells is an attractive target to develop new treatment strategies against cancer [6, 7]. An elevated cellular ROS level damages the cellular nucleotide pool, mainly by oxidizing free nucleotides, and incorporation of these nucleotides into DNA frequently results in manifestation of mutations and cell death [4, 8]. One of the most occurring DNA base-damage caused by ROS is the formation of 8-oxo-dGTP in the nucleotide pool, which causes G:C to T:A transversion mutations when incorporated to the DNA [9]. The nucleotide pool-sanitizing enzyme MTH1 was shown to be of pivotal importance for the progression and the survival of cancer cells as it degrades 8-oxo-dGTP as well as 2-OH-dATP to their respective monophosphatic states, which can then be discarded from the nucleotide pool, thus preventing their incorporation into the DNA [10, 11].

Recently, several new small-molecule inhibitors targeting the nucleotide-sanitizing enzyme MTH1 (TH287, TH588 and S-crizotinib) were described to specifically induce lethality in a broad spectrum of cancer cells without harming untransformed tissues [12–14]. One of them, TH588 was shown to directly interact with the active site of MTH1 via its aminopyrimidine moiety, as revealed by x-ray crystallography studies [12]. Thereby, TH588 interferes with the binding and sanitation of ROS damaged nucleotides by MTH1, leading to persistence of these damaged nucleotides in the cell and thus, to their incorporation into the DNA while DNA replication culminating in increased levels of cell death in cancer cells [12–14].

However the validity and importance of MTH1 as a novel promising target against cancer has been questioned recently [12–14]. The significance of MTH1 as a target for cancer treatment was questioned by several recent reports, in part by the fact that knockdowns of MTH1 by RNA interference (RNAi) or CRISPR technology failed to mirror the effects that were observed with the inhibitors [12–14]. This also led to the hypothesis that the anti-proliferative effects that were achieved by these first-line MTH1 inhibitors have to be attributed to off-target effects, rather than to inhibition of MTH1 [12, 14, 15].

Our aim was to contribute to the assessment of possible cellular mechanisms and molecular signaling pathways implicated in the effects of TH588 using dual-targeting approaches. Using a panel of heterogeneous neuroendocrine tumor (NET) cell lines, we tested TH588 alone or in combination with the mTOR inhibitor everolimus, 5-fluorouracil (5-FU) and γ-irradiation and found that found that TH588 induces cancer cell death by downregulating the PI3K-Akt-mTOR axis and apoptosis induction and these effects are augmented when TH588 is combined with everolimus or 5-fluorouracil. TH588 also exhibited a radiosensitizing potential when being combined with irradiation (radiotherapy), one of most important treatment modalities in nowadays cancer treatment [16]. Our data thus not only provide insights into how TH588 kills cancer cells but also depict novel perspectives for combinatorial treatment approaches encompassing TH588.

## Materials and methods

### Cell culture

The human pancreatic neuroendocrine BON1 tumor cell line [17] was kindly provided by Prof. R. Göke, Marburg, Germany and the pancreatic islet tumor cell line QGP1 [18] was acquired from JCRB Cell Bank (Japanese Collection of Research Bioresources Cell Bank). Human bronchopulmonary neuroendocrine NCI-H727 (H727) tumor cells [19] were purchased from ATCC, Manassas, VA and human midgut carcinoid GOT1 cells [20] were kindly provided by Prof. O. Nilsson, Sahlgrenska University Hospital Göteborg, Sweden. All cell lines used in this study were certified by the German Biological Resource Centre DSMZ (DSMZ, Braunschweig, Germany) using short tandem repeats (STRs) analysis. All human neuroendocrine cell lines were tested mycoplasma free and cultured as described [21, 22]. Cells were grown in DMEM/F12 (1:1) (Life Technologies, Karlsruhe, Germany) (BON1, QGP1) or RPMI-1640 (Sigma-Aldrich, Taufkirchen, Germany) (NCI-H727, GOT1), supplemented each with 10% FBS (Biochrom, Berlin, Germany), 100 unit / ml penicillin, 100 μg / ml streptomycin (Life Technologies) and 1μg / ml amphotericin B (Biochrom, Berlin, Germany) at 37°C and 5% CO2. The GOT1 culture medium was additionally supplemented with 5 μg/mL apo-transferrin and 0.135 IU/mL insulin.

The MTH1 inhibitor TH588 was kindly provided by T. Helleday (Karolinska Institutet, Stockholm). Everolimus and 5-fluorouracil (5-FU) were purchased from Selleckchem (Houston, TX, USA). All three substances were dissolved in dimethyl-sulfoxide (DMSO, Sigma-Aldrich), at 10 mM stock concentration and stored at -20°C.

### Cell viability assessment

Cells were seeded into 96-well plates at densities of 1,500 (BON1), 2,000 (QGP1 and NCI-H727) and 30,000 (GOT1) cells per well and grown for 24 h. Subsequently, cells were treated with different concentrations of TH588, either alone or in combination with 10 nM everolimus or 5 μM 5-FU. Metabolic activity was assessed by the “Cell Titer Blue®” cell viability assay (Promega, Madison, WI, USA) after 96 h and 144 h of incubation. Therefore, cells were incubated for 4 h with Cell Titer Blue® solution and fluorescence was measured at 560/590 nm using a GLOMAX plate reader (Promega, Madison, WI, USA).

### Cell cycle analysis by flow cytometric analysis (FACS)

Cell cycle phase distribution was analyzed using propidium iodide staining and flow cytometry (BD Accuri C6 Analysis, BD Biosciences, Heidelberg, Germany). Cells were cultured in 6-well plates for 24 h. Subsequently, medium was replaced by fresh medium and cells were incubated with different concentrations of TH588. After 72 h, cells were washed with PBS and treated with 300 μl trypsin for 5 min. at 37°C. Cells were collected, washed and resuspended in 300 μl propidium iodide (Sigma-Aldrich).

### Protein extraction and Western blotting

For Western blot experiments, cells were seeded into 10 cm plates and grown for 24 h in complete medium. Then medium was replaced by fresh medium and cells were incubated with different concentrations of TH588 (5 μM and 10 μM), either alone or in combination with 5-FU (5 μM) or everolimus (10 nM). The incubation times were up to 96 h. Western blotting was conducted as described previously [23]. The following primary antibodies used were: pAKT (Ser473) (#4060), AKT (#2920), pERK1/2 (Thr202/Tyr204) (#4370), p4EBP1 (Ser65) (#9451), 4EBP1 (#9644), pRb (Ser780) (#9307), pCDK1 (Tyr15) (#4539), CDK1 (#9116), Cyclin B1 (#12231), Cyclin D1 (# 2926), Cyclin D3 (#2936), CDK4 (#12790), CDK6 (#13331), Chk1 (#2360), pChk2 (Ser19) (#2666), pChk2 (Thr68) (#6334), Chk2 (#6334), Parp (#9542), PCNA (#2586) (all from CellSignaling, Danvers, USA), p16 INK4A (ab151303) (abcam, Cambridge, UK), Rb (#614602) (Biolegend, San Diego, USA), Actin (A5441) (Sigma, St.Louis, USA), ERK1/2 (06–182) (Merck-Millipore, Darmstadt, Germany).

### Caspase-3/-7 activity assay

To measure activity of caspase-3/-7 we used the Apo-One homogeneous caspase-3/7- Assay kit (Promega). 10,000 cells were seeded per well, incubated for 72 h with respective doses of TH588 (5 μM or 10 μM) and caspase-3/7 activity was assessed according to the manufacturer’s instructions.

### γ-irradiation treatment

Cells were irradiated at indicated doses with an RS-225 cabinet (200 kV and 10 mA, Thoraeus filter, 1 Gy in 1 min 5 s; Xstrahl, Camberley, UK) as described [24].

### Colony formation assays

Clonogenic survival was examined by colony formation assay. BON1 or QGP1 cells were seeded as single cell suspensions into 6-well plates in a range of 200–400,000 or 200–200,000 cells per well, respectively. After 4 h of adherence, cells were treated with 2.5 μM TH588 or DMSO as vehicle control and incubated for one additional hour before being irradiated at the indicated doses. Colony formation was allowed for 27 days before cells were fixed and stained in 80% ethanol containing 0.3% methylene blue. Colonies containing more than 50 cells were counted and the percentage of surviving cells was normalized to the plating efficiency.

### Oxidative stress assay

To measure oxidative stress levels, relative concentration of total glutathione (GSSG/GSH) were determined using the OxiSelect^TM^ Total Glutathione GSSG/GSH Assay kit (Cell Biolabs, Inc., CA, USA) in BON1 cells. 900,000 cells were seeded in 10 cm plates and 24 h later the cells were treated with TH588 (5 μM) alone and in combination with 10 nM everolimus or 5 μM 5-FU. After 96 h of incubation the cells were collected and relative GSSG/GSH ratio was assessed according to the manufacturer’s instructions.

### Statistical analysis

The results are displayed as mean ± standard deviation of the mean (SD) of at least three independently performed experiments. *A priori* Tests considering the normal distribution and homogeneity of variances were performed applying the Kolmogorov-Smirnov-Test and the Levene´s Test of the SPSS statistical package SPSS (version 13.0 for Windows, SPSS Inc (2005), Chicago, USA). When parametric criteria were met an ANOVA comparison of means with a *post hoc* Tukey test or a two-tailed t-test was performed; when non parametric criteria were met the Kruskal-Wallis followed by the Mann-Whitney test was performed. Statistical significance was assessed at p<0.05.

## Results

### TH588 effectively decreased cellular survival in heterogeneous neuroendocrine tumor cells

**Fig 1:**
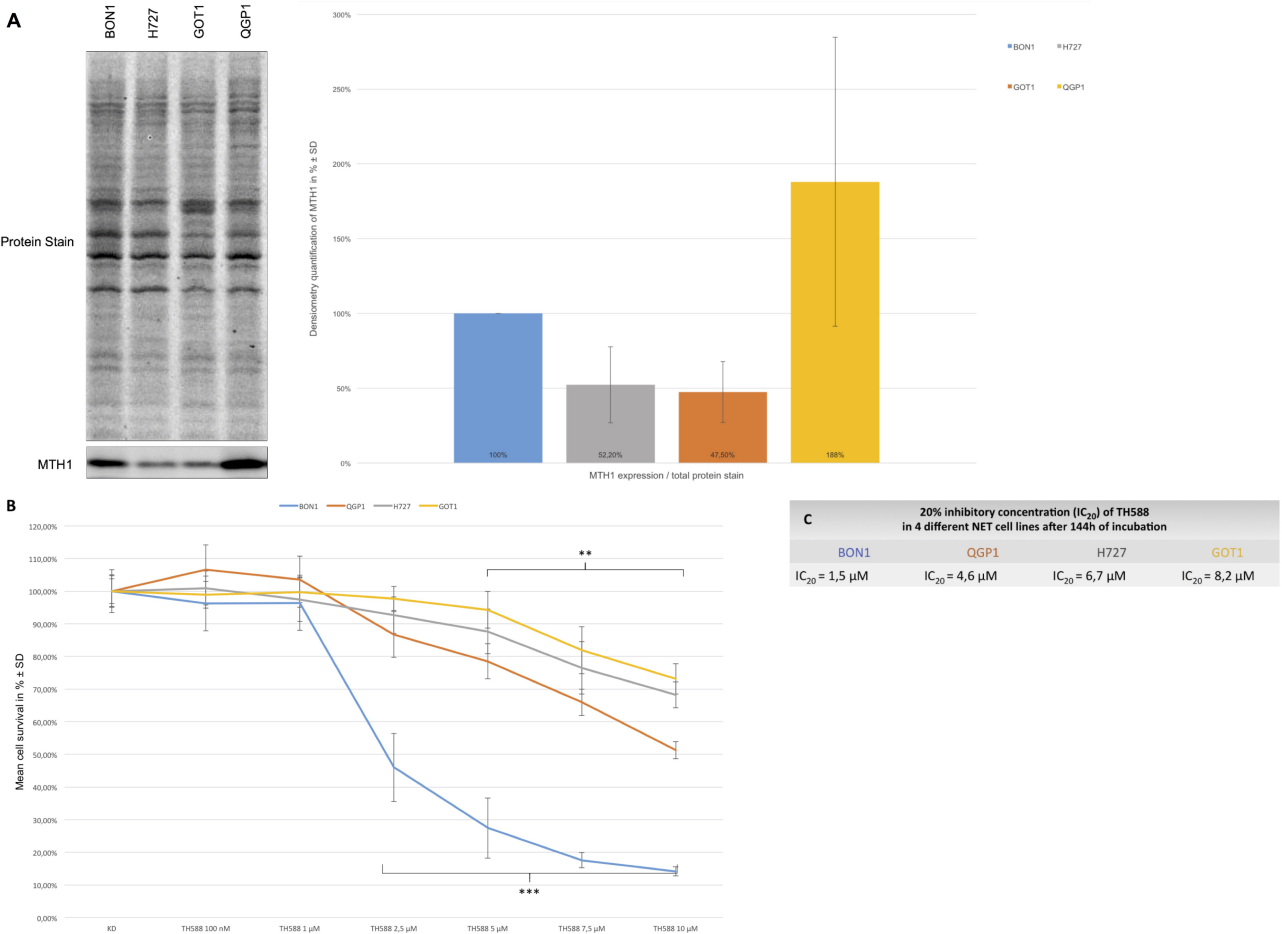
TH588 effectively decreases cellular survival in heterogeneous neuroendocrine tumor cells. (A) Basal protein expression level of endogenous MTH1 in all four NET cell lines. The expression of MTH1 is evaluated by Western blot analysis. A representative blot out of three independently performed experiments is shown, together with densitometry quantification of 3 independent Western blots. (B) The effects of different concentrations of TH588 (100 nM to 10 μM) on cellular survival in neuroendocrine pancreatic BON1, pancreatic islet QGP1, bronchopulmonary H727 and ileal GOT1 cells are displayed after 144 h of incubation. The arithmetic means and standard deviation of at least three independent experiments are shown. Statistical significant different results in comparison to either single substance treatment are shown considering p<0,05 = *; p<0,01 = **; p<0,001 = ***. (C) 20% inhibitory concentration (IC_20_) of TH588 (100 nM to 10 μM) in four different NET cell lines after 144 h of incubation.

The initially as MTH1 inhibitor raised compound TH588 was characterized in multiple entities of cancer cell lines [25]. However, TH588 was never tested for efficacy in the treatment of cancer cells derived from heterogeneous neuroendocrine tumors (NETs). Therefore, we took a panel of four different NET cell lines (pancreatic BON1, pancreatic islet QGP1, bronchopulmonary H727 and ileal GOT1) and tested them on their respective protein expression levels of MTH1 (Fig 1A). Two of these cell lines (BON1 and QGP1) showed a high expression of MTH1 while the other two cell lines (H727 and GOT1) only showed slight levels of MTH1 expression (Fig 1A). We next tested whether the treatment with different doses of TH588 impacts cellular viability in each of these cell lines (Fig 1B). All cell lines showed susceptibility towards TH588 treatment in a time- and dose- dependent manner, albeit to different extends (Fig 1B). While TH588 at 2,5 μM of concentration had only a minor effect on the survival of GOT1, H727 and QGP1 cells, it reduced the survival of BON1 cells to less than 50% (Fig 1B). We calculated the IC_20_ values for all cell lines again finding that BON1 cells were most sensitive to TH588 (IC_20_ 1,5 μM) while GOT1 cells showed the lowest sensitivity towards TH588 (IC_20_ 8,2 μM) (Fig 1C). However, these results are only reflected in part by the protein expression levels of MTH1 since QGP1 cells, which express MTH1 to highest levels (Fig 1A), only show moderate susceptibility towards TH588 (IC_20_ 4,6 μM) (Fig 1C).

### TH588 treatment causes apoptotic cell death

**Fig 2:**
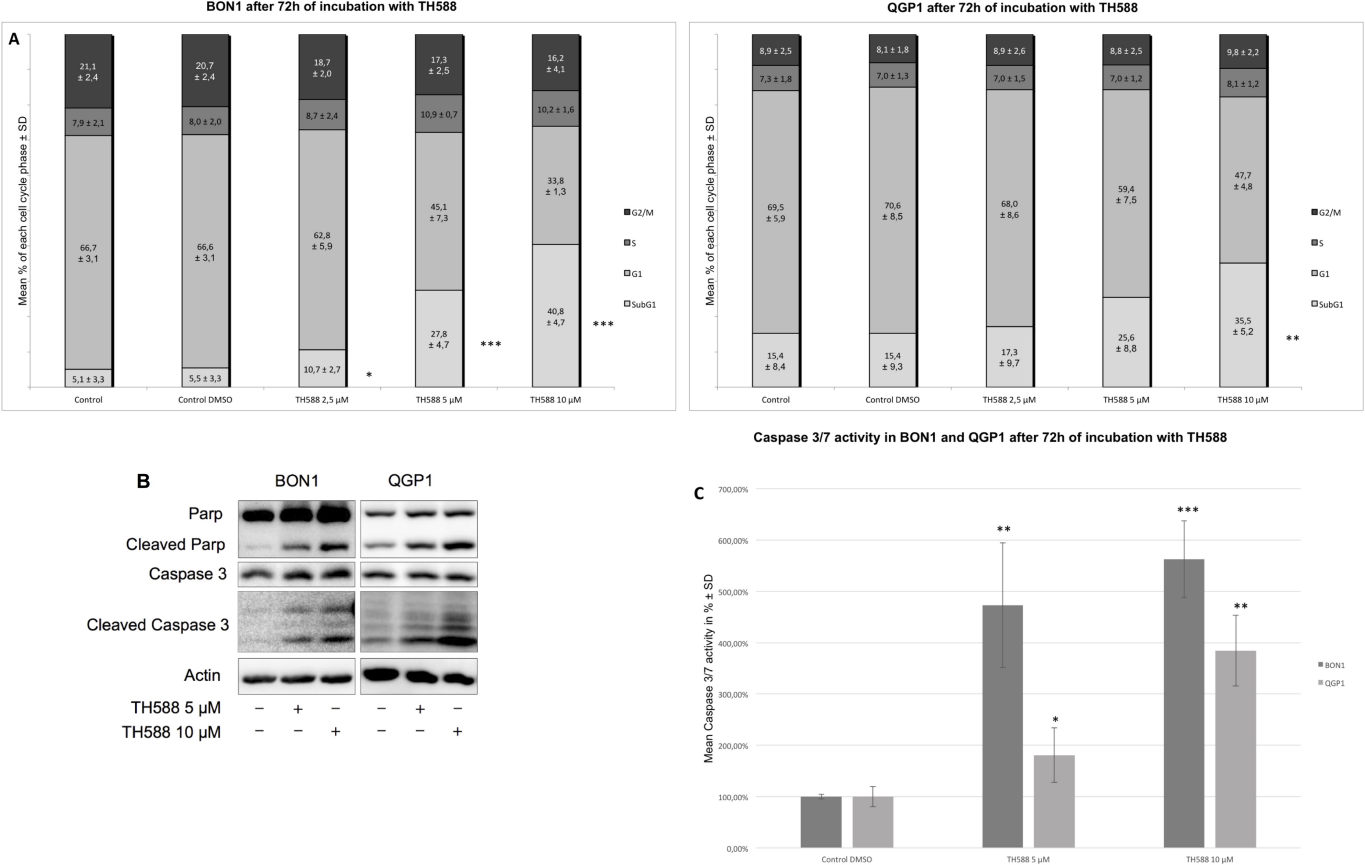
TH588 treatment causes apoptotic cell death. (A) FACS analysis of BON1 and QGP1 cells after 72 h of incubation with TH588. The arithmetic means and standard deviation of at least three independent experiments are shown. Statistical significant different results in comparison to either sinlge substance treatment are shown considering p<0,05 = *; p<0,01 = **; p<0,001 = ***. (B) Western blot analysis of PARP and Caspase 3 cleavage in NETs. A representative blot out of three independently performed experiments is shown. (C) Caspase 3/7 activity in BON1 and QGP1 cells upon 72 h of incubation with TH588. The arithmetic means and standard deviation of at least three independent experiments are shown. Statistical significant different results in comparison to either sinlge substance treatment are shown considering p<0,05 = *; p<0,01 = **; p<0,001 = ***.

To test whether TH588 induces apoptosis in NET cells we calculated the amounts of cells that exhibit sub-G1 genomic contents after exposure to TH588 (Fig 2A). We used the BON1 and the QGP1 cell line as these showed highest vulnerability towards TH588 (Fig 1B). In both cell lines, TH588 induced significant populations of sub-G1 cells in a striking dose-dependent manner (Fig 2A). This was accompanied by a concomitant decrease of G1 phase cells while other cell cycle phases remained unaffected. To confirm that these sub-G1 cells were indeed apoptotic we performed Western blot analysis detecting for proteolytic cleavage of the procaspase-3 and the prototype caspase substrate PARP (Fig 2B). In both cell lines, treatment with TH588 led to substantial cleavage of PARP–as well as of caspase-3 (Fig 2B), confirming apoptosis induction. We also measured the proteolytic activity of caspases 3 and 7 (Fig 2C) in an *in vitro* assay, which confirmed that treatment with TH588 induces apoptosis in NET cells.

### TH588 treatment causes downregulation of the PI3K-Akt-mTOR pathway and of pathway related growth factor receptors

**Fig 3:**
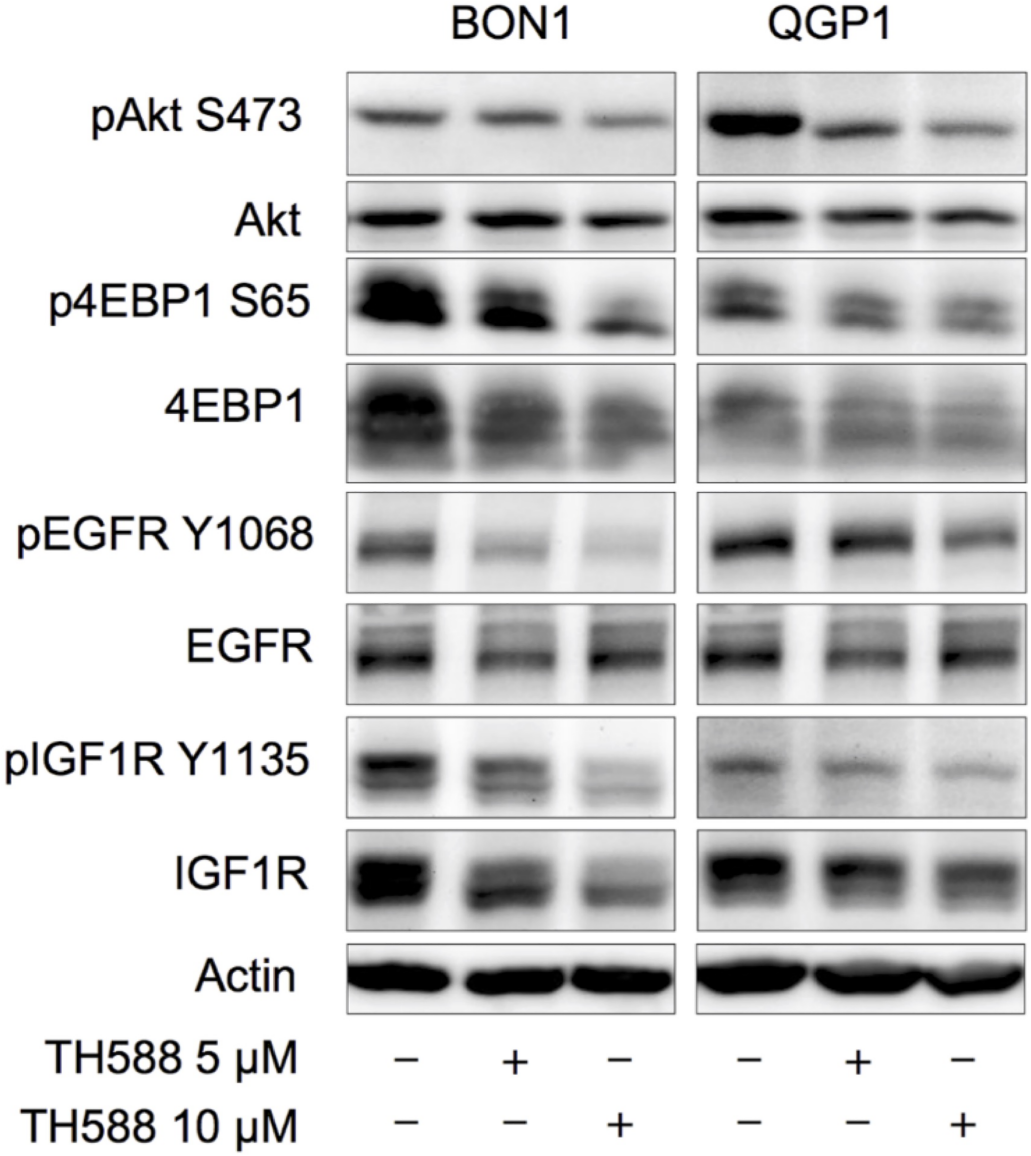
TH588 causes PI3K-Akt-mTOR pathway and pathway related growth factor receptor downregulation. Western blot analysis of components from the PI3K-Akt-mTOR pathway (Akt and 4EBP1) and 2 types of pathway related growth factor receptors (EGFR and IGFR) were analysed after 72 h of incubation with TH588 (5 μM and 10 μM). A representative blot out of three independently performed experiments is shown.

We also investigated whether TH588 affects the PI3K-Akt-mTOR pathway, the major cellular signaling cascade that regulates cell cycle progression, cell proliferation and quiescence [26]. For that we performed Western blot analysis detecting the activatory phosphorylations of Akt (S473) and 4EBP1 (S65) in BON1- and QGP1 cells treated with TH588 (Fig 3). We found that activatory phosphorylation of both, Akt and 4EBP1 declined upon exposure to TH588 in a dose-dependent manner (Fig 3). This is further supported by the finding that activatory phosphorylations of the respective growth factor receptors (EGFR Y1068 and IGF1R Y1135) are also decreased upon TH588 treatment (Fig 3).

### Dual-targeting approaches show additive effects in cell survival decrease, which are mirrored by apoptotic cell death enhancement and cooperative PI3K-Akt-mTOR pathway downregulation

**Fig 4:**
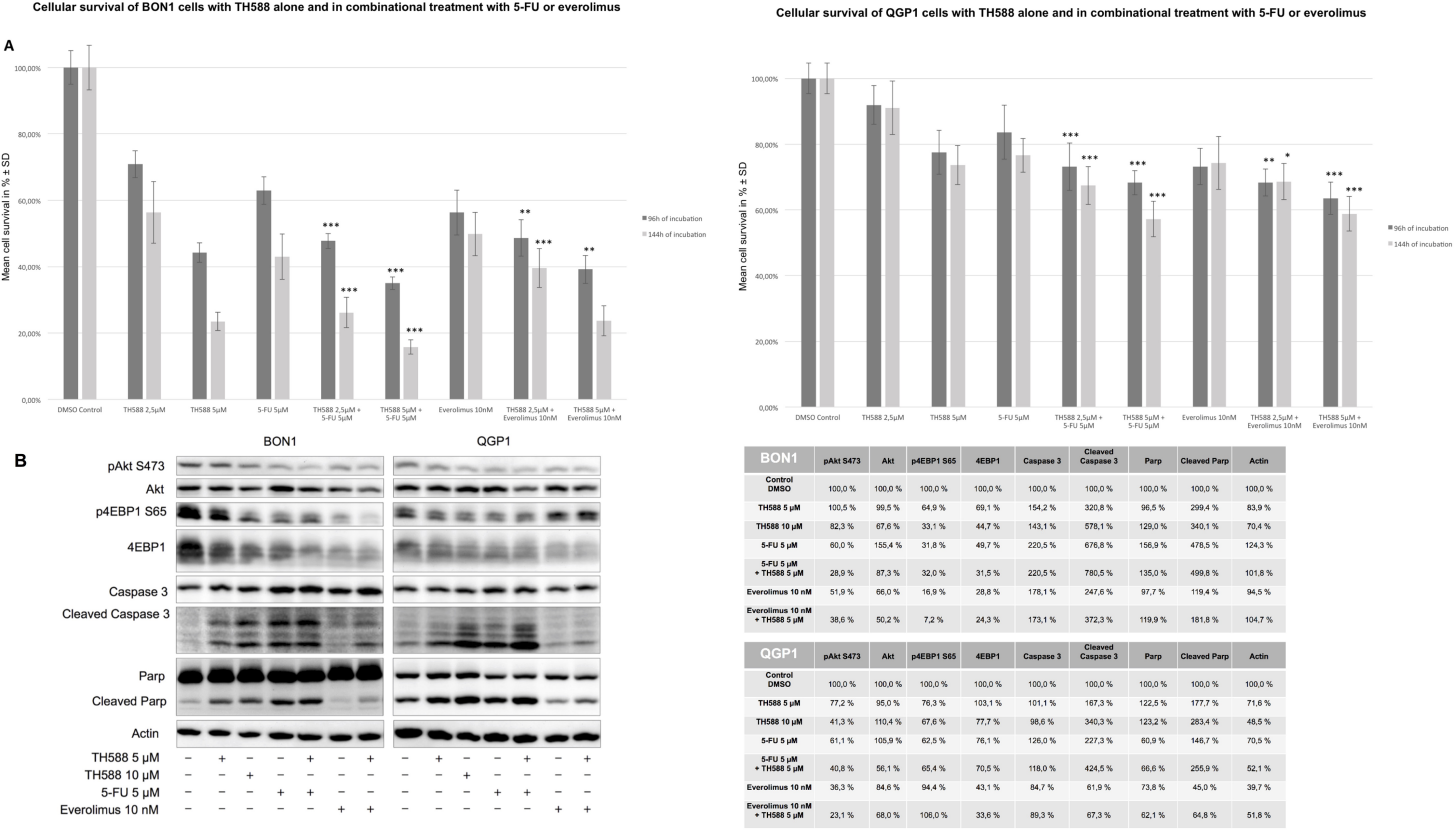
Dual-targeting approaches show agonistic effects in cell survival decrease due to either apoptotic cell death enhancement or cooperative PI3K-Akt-mTOR pathway downregulation. (A) Effect of TH588 on cell survival. Human neuroendocrine pancreatic BON1 and pancreatic islet QGP1 cells were incubated with TH588 (5 μM and 10 μM) alone and in combination (TH588 (5 μM)) with 5-FU (5 μM) and everolimus (10 nM) for 96 h and 144 h. The arithmetic means and standard deviation of at least three independent experiments are shown. Statistical significant different results in comparison to either sinlge substance treatment are shown, considering p<0,05 = *; p<0,01 = **; p<0,001 = ***. (B) Western blot analysis components from PI3K-Akt-mTOR pathway and the apoptotic cell apparatus were analyzed with TH588 alone (5 μM and 10 μM) alone and in combination with 5-FU (5 μM) and everolimus (10 nM) after 96 h. A representative blot out of three independently performed experiments is shown, together with the densitometry quantification.

Due to the pro-apoptotic effects and the down-regulation of the PI3K-Akt-mTOR pathway in response to TH588 treatment, we decided to combine TH588 with a substance affecting either pathway in order to further investigate and confirm the effects of TH588 and to assess possible agonistic effects of the combinational treatment (Fig 4). We therefore tested TH588 in combination with 5-fluorouracil (5-FU) or the mTORC1 inhibitor everolimus (Fig 4A). In both analysed cell lines, co-administration of TH588 led to an enhancement in the decrease of cell survival when compared to the respective single treatment regime employing 5-FU or everolimus (Fig 4A). To get an insight into how TH588 agonizes with 5-FU and everolimus, we performed Western blot analysis again detecting activatory phosphorylation of Akt and 4EBP1 as well as cleavage of PARP and procaspase-3 (Fig 4B). In BON1 cells the combination of TH588 (5 μM) with either 5-FU (5 μM) or everolimus showed cooperative enhancement over single substance treatment in downregulating pAkt and p4EBP1 respectively (Fig 4B). The apoptotic components, cleaved PARP and cleaved Caspase 3 showed also a slight upregulation enhancement over single substance treatment in BON1 cells (Fig 4B). However QGP1 cells demonstrated a clear enhancement over single substance treatment in PARP- and procaspase 3- cleavage when combined with 5-FU (Fig 4B). Cooperative downregulation of pAkt is only week in QGP1, combining TH588 with 5-FU or everolimus (Fig 4B). Hence, cooperative decrease of cellular survival in the combinational treatment arise from a PI3K-Akt-mTOR downregulation and/or apoptosis up-regulation, depending on the cell line (Fig 4B).

**Fig 5:**
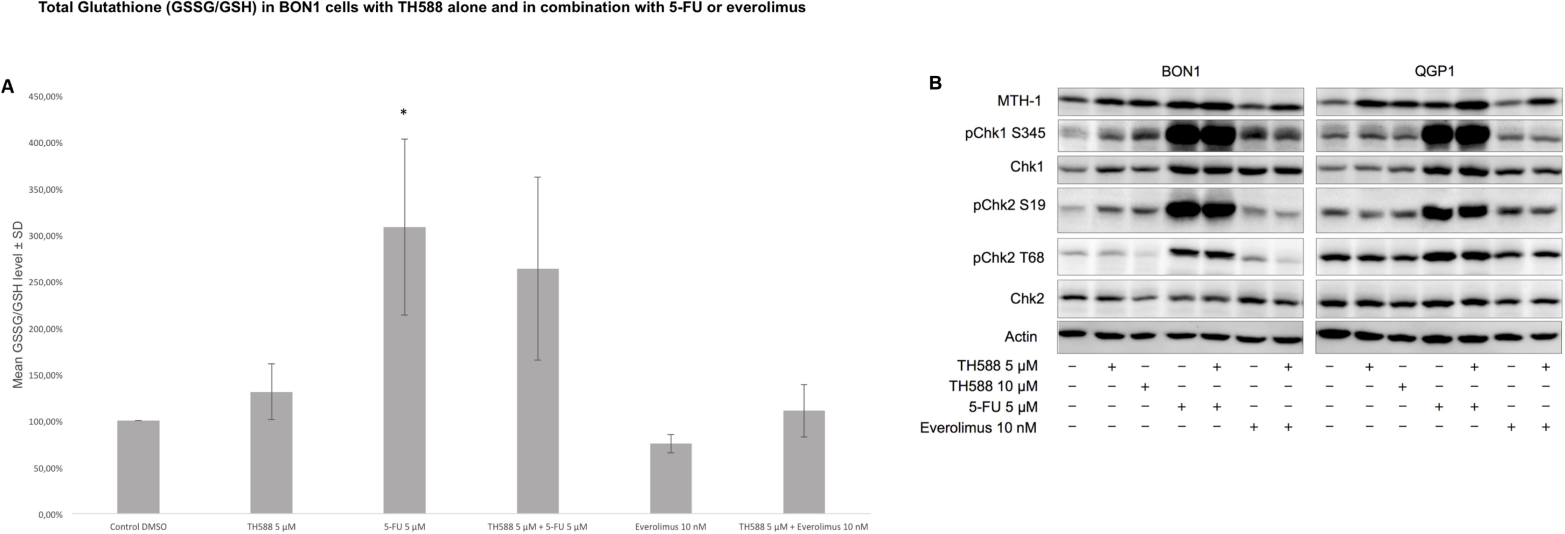
TH588 causes oxidative stress and serves as radio-sensitizing adjuvant. (A) Displayed is the relative oxidative stress after 96 h of incubation with TH588 alone (5 μM) and in combination with 5-FU (5 μM) and everolimus (10 nM). The arithmetic means and standard deviation of at least three independent experiments are shown. Statistical significant different results in comparison to either single substance treatment are shown, considering p<0,05 = *; p<0,01 = **; p<0,001 = ***. (B) Western blot analysis of components from DNA damage response and MTH1 are displayed after 96 h of incubation with TH588 alone and in combination with 5-FU and everolimus. A representative blot out of three independently performed experiments is shown.

We also tested whether co-administration of TH588 and 5-FU or everolimus increases the levels of oxidative stress but could not find any significant differences between combinatorial treatment approaches and the respective single-drug treatments (Fig 5A). Furthermore, co-administration of TH588 did not affect the 5-FU-mediated activation of the DNA damage response as revealed by similar levels of phosphorylation of the checkpoint kinases 1 and 2 and the same accounted for everolimus (Fig 5B). In both cell lines MTH1 is upregulated upon TH588 treatment (Fig 5B).

### TH588 as a radio-sensitizing adjuvant

**Fig 6:**
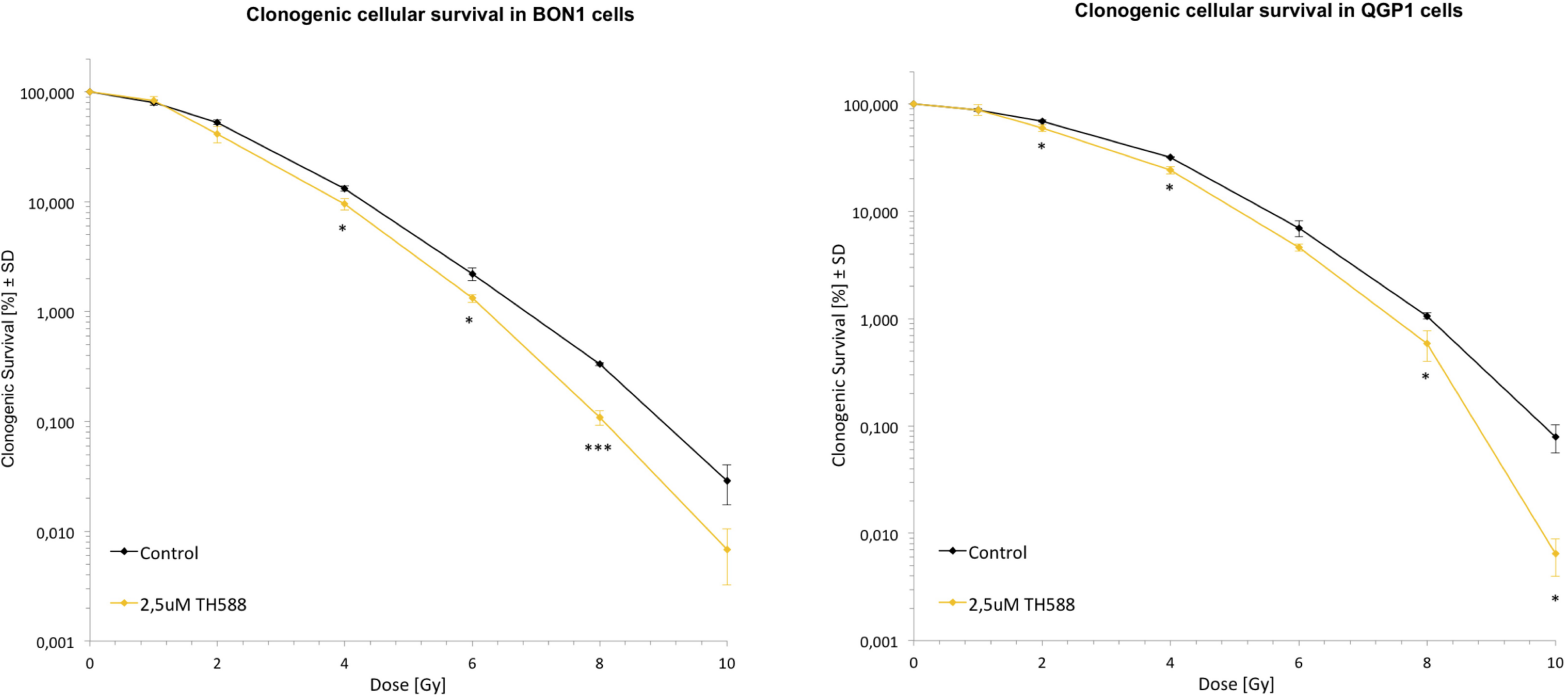
TH588 as a radio-sensitizing adjuvant in BON1 and QGP1 cells. The arithmetic means and standard deviation of at least three independent experiments are shown. Statistical significant different results in comparison to either single x-ray treatment are shown, considering p<0,05 = *; p<0,01 = **; p<0,001 = ***.

Since we and others could show that TH588 impacts the levels of oxidative stress in cancer cells (Fig 5A) and reactive oxygen species (ROS) are a major contributor in terms of facilitating DNA damage, e.g. upon exposure to ionizing irradiation we tested whether TH588 might function as a radiosensitizing agent when combined with irradiation. Therefore performed colony formation assay and indeed found that co-administration of TH588 at 2,5 μM concentration led to radiosensitization of both analyzed NET cell lines (Fig 6). Thus, TH588 might serve as a radiosensitizer in combinatorial treatment approaches involving radiotherapy.

## Discussion

Oxidative stress in cancer has been of great interest lately, as modulation of the redox system offers a new target for anti-cancer strategies [6]. In particular a number of MTH1 inhibitors, including TH588 have been claimed to effectively eradicate cancer cells by elevating the oxidative stress level to a cytotoxic level, causing cancer cell death [25, 27, 28]. However recent publications have questioned the specificity of the MTH1 inhibitors, pointing out off-target effects as principal cause for the anti-proliferative effects of the developed MTH1 inhibitors [12, 14, 15]. Furthermore, the validity and importance of MTH1 for a novel anti-cancer strategy has been challenged, as knock-down and knock-out models have rendered MTH1 redundant for cancer cell survival [12–14]. Recently only gliomatumorigenesis showed MTH1 dependency [29]. Here we bring new insight into the effects of TH588 and contribute to the assessment of the impact of TH588 on cellular signaling pathways. Furthermore we reveal agonistic interactions between the MTH1 inhibitor TH588 and current (state-of-the-art) cancer treatment modalities in neuroendocrine tumors.

All four of our heterogeneous NET cell lines tested showed a time- and dose- dependent decrease of cellular survival after treatment with TH588 (Fig 1B). Similar effects of TH588 regarding cellular survival were shown recently [15, 25, 28]. Our results show a potent induction of apoptotic cell death mechanisms upon TH588 treatment (Fig 2). An increase of apoptosis due to TH588 treatment has also been described lately [15, 25]. The PI3K-Akt-mTOR pathway is one of the principal proliferative pathways and often up-regulated in human cancer [30]. We found that TH588 downregulates crucial components of the PI3K-Akt-mTOR pathway (Fig 3). Additionally, the growth factor receptors IGFR and EGFR, which cause PI3K-Akt-mTOR pathway activation and foment cancer cell proliferation [31, 32] were also downregulated upon TH588-treatment (Fig 3). Our results confirmed a moderate increase in cellular oxidative stress upon TH588 treatment (Fig 5A) and MTH1 was upregulated upon TH588 treatment possibly due to feedback mechanisms (Fig 5B). In addition compounds of the cellular DNA damage response such as pChk1 and pChk2 were upregulated with TH588 treatment (Fig 5B). Similar results regarding oxidative stress increase and DNA damaging effects of TH588 have been reported lately [14, 15, 25]. As a consequence to the effects of single TH558 substance treatment we decided to combine TH588 with either the mTORC1 inhibitor everolimus or the cytotoxic chemotherapeutic agent 5-FU or ionizing radiation in order to confirm our results and to assess possible agonistic combinatory effects of simultaneous pathway signaling inhibition [33–35]. Everolimus is a molecular targeting agent used in oncology, which inhibits mTORC1 and thus downregulates the PI3K-Akt-mTOR pathway [36–38]. The mTOR inhibitor everolimus has proven anti-tumoral efficacy in several clinical phase 3 trials in patients with neuroendocrine tumors [39–41] and is approved for pancreatic, intestinal and pulmonary neuroendocrine tumors [42]. Unfortunately only a subset of patients respond to everolimus treatment due to intrinsic resistance or the development of an acquired resistance in response to long term treatment [43–47]. Thus, dual-targeting approaches in order to overcome resistance against everolimus are investigated [47]. Furthermore Cytotoxic 5-FU and ionizing radiation are commonly implied in the clinic and recognized for their oxidative stress increasing, DNA damaging characteristics [48, 49]. The dual-targeting approach with TH588 and everolimus or 5-FU showed a significant enhancement in cellular survival decrease, in comparison to single substance treatments (Fig 4A). Similar effects of chemo-sensitizing by TH588 were described in a melanoma cancer cell model, where MTH1 inhibition was neglectable for the cancer cells, but sensitized the cancer cells for cytotoxic treatments with elesclomol [15]. The molecular mechanisms beyond the cooperative effects of TH588 and everolimus showed an enhancement of PI3K-Akt-mTOR downregulation, whereas combination with 5-FU demonstrated either an increase in apoptotic cell death or a downregulation of the PI3K-Akt-mTOR pathway (Fig 4B). Interestingly under similar and pre-established experimental conditions, regarding times of incubation and concentration TH588 killed cancer cells more efficiently than 5-FU (S1 Fig), albeit inducing significantly less oxidative stress (Fig 5A). Consequently, the higher efficiency of TH588 in killing cells at similar experimental conditions in comparison to 5-FU indicates that in NET cells, as described for cell lines derived from other cancer entities [12, 14, 15] TH588 abrogates cell survival via alternative mechanisms than inhibition of MTH1 and cytotoxic increase in oxidative stress. Radiotherapy is a crucial factor for clinical anti-cancer treatment approaches [50, 51] and is considered one of the most important treatment modalities in nowadays cancer treatment [16]. Resistance formation and treatment inefficiency of radiotherapy are major reasons for the permanent need of targets to re-sensitize and support radio-therapeutic approaches [49]. Due to the cellular effects of TH588 mentioned previously, we examined the characteristics of TH588 as radio-sensitizer. The combinational treatment of TH588 and ionizing **γ**-irradiation significantly abrogated clonogenic survival and TH588 was allocated radio-sensitizing adjuvant properties (Fig 6).

Four rationales indicate that the cancer cell eradicating effects of TH588 alone or in dual-targeting approaches are attributed to an TH588-mediated increase in apoptosis and PI3K-Akt-mTOR pathway downregulation but not oxidative stress:

- TH588 alone or in combination with 5-FU or everolimus revealed an increase in apoptotic cell death mechanisms and PI3K-Akt-mTOR pathway downregulation (Figs 2, 3 and 4B).
- Dual-targeting approaches with either cytotoxic 5-FU or molecular targeting everolimus could not demonstrate an enhancement in oxidative stress level (Fig 5A)
- Neither combinational treatment revealed an increase in activatory phosphorylation of the major DNA damage response kinases Chk1 and Chk2 versus single substance treatment (Fig 5B)
- TH588 killed cancer cells more efficiently than 5-FU (S1 Fig) at established experimental conditions, but TH588 induced significantly less oxidative stress than 5-FU (Fig 5A). Consequently, the higher efficiency of TH588 in killing cells at similar experimental conditions in comparison to 5-FU stems from the above mentioned off-target effects, not from a MTH1 inhibitory cytotoxic increase in oxidative stress.

## Conclusion

Considering our results, we determine that the MTH1 inhibitor TH588 is an effective substance to decrease cellular viability of heterogeneous tumors such as neuroendocrine tumors. We suggest that anti-proliferative effects of TH588 are attributed to increased apoptosis, increased DNA damage response as revealed by pChk1 or pChk2, increase of oxidative stress levels and downregulation of the PI3K-Akt-mTOR axis. Both dual-targeting strategies of TH588 with either cytotoxic 5-fluorouracil or mTORC1 inhibitor everolimus showed additive effects on cell survival decrease via cellular apoptotic increase and/or PI3K-Akt-mTOR axis downregulation and further confirm the involvement of TH588 in those pathways. The dual-targeting approaches could not achieve an agonistic effect on the oxidative stress level; thus TH588 demonstrated to abrogate cell survival via the above mentioned mechanisms rather than inhibition of MTH1 and cytotoxic increase in oxidative stress. Furthermore, the MTH1 inhibitor TH588 displayed properties as chemo- and radio-sensitizer. Conclusively, our data provided new insights into the cancer eradicating effects of TH588 on cellular mechanisms and also opened up novel perspectives for combined-modality treatment approaches encompassing TH588. We suggest further investigation of the effects of TH588 other than MTH1 inhibition and implications on cellular signaling in order to clarify its role as a possible anti-cancer therapy in the clinic.

## Supporting information

S1 Fig TH588 eradicates NET cells at established conditions more efficiently than 5-FU. Effect of TH588 on cell survival. Human neuroendocrine pancreatic BON1 cells were incubated with TH588 (5 μM) or 5-FU (5 μM) for 96 h. The arithmetic means and standard deviation of at least three independent experiments are shown. Statistical significant different results in comparison to either single substance treatment are shown, considering p<0,05 = *; p<0,01 = **; p<0,001 = ***.
(TIF)

S2 Fig Uncropped Western blot of MTH1 in all tested cell lines. Basal expression of MTH1 in different neuroendocrine cell lines (BON1, H727, GOT1 and QGP1) and in HEPG2 and HUH7 cells.
(TIF)

S3 Fig Uncropped Western blot of MTH1 in all tested cell lines. Basal expression of MTH1 in different neuroendocrine cell lines (BON1, H727, GOT1 and QGP1) and in HEPG2 and HUH7 cells.
(TIF)

S4 Fig Uncropped Western blot of Actin and PCNA expression in combinational treatment. Expression of Actin and PCNA in neuroendocrine cell lines (BON1, H727 and QGP1) after 96 h of incubation with TH588 (5 μM or 10 μM) alone or in combination with 5FU (5 μM) or everolimus (10 nM).
(TIF)

S5 Fig Uncropped Western blot of Caspase 3 and cleaved Caspase 3 expression in combinational treatment. Expression of Caspase 3 and cleaved Caspase 3 in neuroendocrine cell lines (BON1, H727 and QGP1) after 96 h of incubation with TH588 (5 μM or 10 μM) alone or in combination with 5FU (5 μM) or everolimus (10 nM).
(TIF)

S6 Fig Uncropped Western blot of PARP and cleaved PARP expression in combinational treatment. Expression of PARP and cleaved PARP in neuroendocrine cell lines (BON1, H727 and QGP1) after 96 h of incubation with TH588 (5 μM or 10 μM) alone or in combination with 5FU (5 μM) or everolimus (10 nM).
(TIF)

S7 Fig Uncropped Western blot of Actin and PCNA expression in single substance treatment. Expression of Actin and PCNA in BON1 cells after 24 h, 48 h and 72 h of incubation with TH588 (2,5 μM, 5 μM or 10 μM).
(TIF)

S8 Fig Uncropped Western blot of Caspase 3 and cleaved Caspase 3 expression in single substance treatment. Expression of Caspase 3 and cleaved Caspase 3 in BON1 cells after 24 h, 48 h and 72 h of incubation with TH588 (2,5 μM, 5 μM or 10 μM).
(TIF)

S9 Fig Uncropped Western blot of PARP and cleaved PARP expression in single substance treatment. Expression of PARP and cleaved PARP in BON1 cells after 24 h, 48 h and 72 h of incubation with TH588 (2,5 μM, 5 μM or 10 μM).
(TIF)

S10 Fig Uncropped Western blot of Actin and PCNA expression in combinational treatment. Expression of Actin and PCNA in neuroendocrine cell lines (BON1, H727 and QGP1) after 96 h of incubation with TH588 (5 μM or 10 μM) alone or in combination with 5FU (5 μM) or everolimus (10 nM).
(TIF)

S11 Fig Uncropped Western blot of pEGFR, pIGFR, pAkt, pErk, pS6 and p4EBP1 expression in combinational treatment. Expression of pEGFR, pIGFR, pAkt, pErk, pS6 and p4EBP1 in neuroendocrine cell lines (BON1, H727 and QGP1) after 96 h of incubation with TH588 (5 μM or 10 μM) alone or in combination with 5FU (5 μM) or everolimus (10 nM).
(TIF)

S12 Fig Uncropped Western blot of pEGFR, pIGFR, pAkt, pErk, pS6 and p4EBP1 expression in combinational treatment. Expression of pEGFR, pIGFR, pAkt, pErk, pS6 and p4EBP1 in neuroendocrine cell lines (BON1, H727 and QGP1) after 96 h of incubation with TH588 (5 μM or 10 μM) alone or in combination with 5FU (5 μM) or everolimus (10 nM).
(TIF)

S13 Fig Uncropped Western blot of Caspase 3 and cleaved Caspase 3 expression in combinational treatment. Expression of Caspase 3 and cleaved Caspase 3 in neuroendocrine cell lines (BON1, H727 and QGP1) after 96 h of incubation with TH588 (5 μM or 10 μM) alone or in combination with 5FU (5 μM) or everolimus (10 nM).
(TIF)

S14 Fig Uncropped Western blot of Caspase 3 and cleaved Caspase 3 expression in combinational treatment. Expression of Caspase 3 and cleaved Caspase 3 in neuroendocrine cell lines (BON1, H727 and QGP1) after 96 h of incubation with TH588 (5 μM or 10 μM) alone or in combination with 5FU (5 μM) or everolimus (10 nM).
(TIF)

S15 Fig Uncropped Western blot of Caspase 3 and cleaved Caspase 3 expression in combinational treatment. Expression of Caspase 3 and cleaved Caspase 3 in neuroendocrine cell lines (BON1, H727 and QGP1) after 96 h of incubation with TH588 (5 μM or 10 μM) alone or in combination with 5FU (5 μM) or everolimus (10 nM).
(TIF)

S16 Fig Uncropped Western blot of pEGFR, pIGFR, pAkt, pErk, pS6 and p4EBP1 expression in combinational treatment. Expression of pEGFR, pIGFR, pAkt, pErk, pS6 and p4EBP1 in neuroendocrine cell lines (BON1, H727 and QGP1) after 96 h of incubation with TH588 (5 μM or 10 μM) alone or in combination with 5FU (5 μM) or everolimus (10 nM).
(TIF)

S17 Fig Uncropped Western blot of pEGFR, pIGFR, pAkt, pErk, pS6 and p4EBP1 expression in combinational treatment. Expression of pEGFR, pIGFR, pAkt, pErk, pS6 and p4EBP1 in neuroendocrine cell lines (BON1, H727 and QGP1) after 96 h of incubation with TH588 (5 μM or 10 μM) alone or in combination with 5FU (5 μM) or everolimus (10 nM).
(TIF)

S18 Fig Uncropped Western blot of pEGFR, pIGFR, pAkt, pErk, pS6 and p4EBP1 expression in combinational treatment. Expression of pEGFR, pIGFR, pAkt, pErk, pS6 and p4EBP1 in neuroendocrine cell lines (BON1, H727 and QGP1) after 96 h of incubation with TH588 (5 μM or 10 μM) alone or in combination with 5FU (5 μM) or everolimus (10 nM).
(TIF)

S19 Fig Uncropped Western blot of EGFR, IGFR, Akt, Erk, S6 and 4EBP1 expression in combinational treatment. Expression of EGFR, IGFR, Akt, Erk, S6 and 4EBP1 in neuroendocrine cell lines (BON1, H727 and QGP1) after 96 h of incubation with TH588 (5 μM or 10 μM) alone or in combination with 5FU (5 μM) or everolimus (10 nM).
(TIF)

S20 Fig Uncropped Western blot of EGFR, IGFR, Akt, Erk, S6 and 4EBP1 expression in combinational treatment. Expression of EGFR, IGFR, Akt, Erk, S6 and 4EBP1 in neuroendocrine cell lines (BON1, H727 and QGP1) after 96 h of incubation with TH588 (5 μM or 10 μM) alone or in combination with 5FU (5 μM) or everolimus (10 nM).
(TIF)

S21 Fig Uncropped Western blot of EGFR, IGFR, Akt, Erk, S6 and 4EBP1 expression in combinational treatment. Expression of EGFR, IGFR, Akt, Erk, S6 and 4EBP1 in neuroendocrine cell lines (BON1, H727 and QGP1) after 96 h of incubation with TH588 (5 μM or 10 μM) alone or in combination with 5FU (5 μM) or everolimus (10 nM).
(TIF)

S22 Fig Uncropped Western blot of PARP and cleaved PARP expression in combinational treatment. Expression of PARP and cleaved PARP in neuroendocrine cell lines (BON1, H727 and QGP1) after 96 h of incubation with TH588 (5 μM or 10 μM) alone or in combination with 5FU (5 μM) or everolimus (10 nM).
(TIF)

S23 Fig Uncropped Western blot of PARP and cleaved PARP expression in combinational treatment. Expression of PARP and cleaved PARP in neuroendocrine cell lines (BON1, H727 and QGP1) after 96 h of incubation with TH588 (5 μM or 10 μM) alone or in combination with 5FU (5 μM) or everolimus (10 nM).
(TIF)

S24 Fig Uncropped Western blot of PARP and cleaved PARP expression in combinational treatment. Expression of PARP and cleaved PARP in neuroendocrine cell lines (BON1, H727 and QGP1) after 96 h of incubation with TH588 (5 μM or 10 μM) alone or in combination with 5FU (5 μM) or everolimus (10 nM).
(TIF)

S25 Fig Uncropped Western blot of Actin and PCNA expression in combinational treatment. Expression of Actin and cleaved PCNA in neuroendocrine cell lines (BON1, H727 and QGP1) after 96 h of incubation with TH588 (5 μM or 10 μM) alone or in combination with 5FU (5 μM) or everolimus (10 nM).
(TIF)

S26 Fig Uncropped Western blot of Actin and PCNA expression in combinational treatment. Expression of Actin and cleaved PCNA in neuroendocrine cell lines (BON1, H727 and QGP1) after 96 h of incubation with TH588 (5 μM or 10 μM) alone or in combination with 5FU (5 μM) or everolimus (10 nM).
(TIF)

S27 Fig Uncropped Western blot of Actin and PCNA expression in combinational treatment. Expression of Actin and cleaved PCNA in neuroendocrine cell lines (BON1, H727 and QGP1) after 96 h of incubation with TH588 (5 μM or 10 μM) alone or in combination with 5FU (5 μM) or everolimus (10 nM).
(TIF)

S28 Fig Uncropped Western blot of Chk1, CDK1, Bcl2 and p21 expression in combinational treatment. Expression of Chk1, CDK1, Bcl2 and p21 in neuroendocrine cell lines (BON1, H727 and QGP1) after 96 h of incubation with TH588 (5 μM or 10 μM) alone or in combination with 5FU (5 μM) or everolimus (10 nM).
(TIF)

S29 Fig Uncropped Western blot of Chk1, CDK1, Bcl2 and p21 expression in combinational treatment. Expression of Chk1, CDK1, Bcl2 and p21 in neuroendocrine cell lines (BON1, H727 and QGP1) after 96 h of incubation with TH588 (5 μM or 10 μM) alone or in combination with 5FU (5 μM) or everolimus (10 nM).
(TIF)

S30 Fig Uncropped Western blot of Chk1, CDK1, Bcl2 and p21 expression in combinational treatment. Expression of Chk1, CDK1, Bcl2 and p21 in neuroendocrine cell lines (BON1, H727 and QGP1) after 96 h of incubation with TH588 (5 μM or 10 μM) alone or in combination with 5FU (5 μM) or everolimus (10 nM).
(TIF)

S31 Fig Uncropped Western blot of Chk2, CDK4 and CDK6 expression in combinational treatment. Expression of Chk2, CDK4 and CDK6 in neuroendocrine cell lines (BON1, H727 and QGP1) after 96 h of incubation with TH588 (5 μM or 10 μM) alone or in combination with 5FU (5 μM) or everolimus (10 nM).
(TIF)

S32 Fig Uncropped Western blot of Chk2, CDK4 and CDK6 expression in combinational treatment. Expression of Chk2, CDK4 and CDK6 in neuroendocrine cell lines (BON1, H727 and QGP1) after 96 h of incubation with TH588 (5 μM or 10 μM) alone or in combination with 5FU (5 μM) or everolimus (10 nM).
(TIF)

S33 Fig Uncropped Western blot of Chk2, CDK4 and CDK6 expression in combinational treatment. Expression of Chk2, CDK4 and CDK6 in neuroendocrine cell lines (BON1, H727 and QGP1) after 96 h of incubation with TH588 (5 μM or 10 μM) alone or in combination with 5FU (5 μM) or everolimus (10 nM).
(TIF)

S34 Fig Uncropped Western blot of MTH1 expression in combinational treatment. Expression of MTH1 in neuroendocrine cell lines (BON1, H727 and QGP1) after 96 h of incubation with TH588 (5 μM or 10 μM) alone or in combination with 5FU (5 μM) or everolimus (10 nM).
(TIF)

S35 Fig Uncropped Western blot of MTH1 expression in combinational treatment. Expression of MTH1 in neuroendocrine cell lines (BON1, H727 and QGP1) after 96 h of incubation with TH588 (5 μM or 10 μM) alone or in combination with 5FU (5 μM) or everolimus (10 nM).
(TIF)

S36 Fig Uncropped Western blot of MTH1 expression in combinational treatment. Expression of MTH1 in neuroendocrine cell lines (BON1, H727 and QGP1) after 96 h of incubation with TH588 (5 μM or 10 μM) alone or in combination with 5FU (5 μM) or everolimus (10 nM).
(TIF)

S37 Fig Uncropped Western blot of pChk1 and pCDK1 expression in combinational treatment. Expression of pChk1 and pCDK1 in neuroendocrine cell lines (BON1, H727 and QGP1) after 96 h of incubation with TH588 (5 μM or 10 μM) alone or in combination with 5FU (5 μM) or everolimus (10 nM).
(TIF)

S38 Fig Uncropped Western blot of pChk1 and pCDK1 expression in combinational treatment. Expression of pChk1 and pCDK1 in neuroendocrine cell lines (BON1, H727 and QGP1) after 96 h of incubation with TH588 (5 μM or 10 μM) alone or in combination with 5FU (5 μM) or everolimus (10 nM).
(TIF)

S39 Fig Uncropped Western blot of pChk1 and pCDK1 expression in combinational treatment. Expression of pChk1 and pCDK1 in neuroendocrine cell lines (BON1, H727 and QGP1) after 96 h of incubation with TH588 (5 μM or 10 μM) alone or in combination with 5FU (5 μM) or everolimus (10 nM).
(TIF)

S40 Fig Uncropped Western blot of pChk2 expression in combinational treatment. Expression of pChk2 in neuroendocrine cell lines (BON1, H727 and QGP1) after 96 h of incubation with TH588 (5 μM or 10 μM) alone or in combination with 5FU (5 μM) or everolimus (10 nM).
(TIF)

S41 Fig Uncropped Western blot of pChk2 expression in combinational treatment. Expression of pChk2 in neuroendocrine cell lines (BON1, H727 and QGP1) after 96 h of incubation with TH588 (5 μM or 10 μM) alone or in combination with 5FU (5 μM) or everolimus (10 nM).
(TIF)

S42 Fig Uncropped Western blot of pChk1, pChk2, pCDK1 and p27 expression in combinational treatment. Expression of pChk1, pChk2, pCDK1 and p27 in neuroendocrine cell lines (BON1, H727 and QGP1) after 96 h of incubation with TH588 (5 μM or 10 μM) alone or in combination with 5FU (5 μM) or everolimus (10 nM).
(TIF)

S43 Fig Uncropped Western blot of pRb, pChk1, pChk2, pCDK1 and p27 expression in combinational treatment. Expression of pRb, pChk1, pChk2, pCDK1 and p27 in neuroendocrine cell lines (BON1, H727 and QGP1) after 96 h of incubation with TH588 (5 μM or 10 μM) alone or in combination with 5FU (5 μM) or everolimus (10 nM).
(TIF)

S44 Fig Uncropped Western blot of pChk1, pChk2, pCDK1 and p27 expression in combinational treatment. Expression of pChk1, pChk2, pCDK1 and p27 in neuroendocrine cell lines (BON1, H727 and QGP1) after 96 h of incubation with TH588 (5 μM or 10 μM) alone or in combination with 5FU (5 μM) or everolimus (10 nM).
(TIF)
