# Supplementary figures and images for: The MTH1 inhibitor TH588 demonstrates anti-tumoral effects alone and in combination with everolimus, 5-FU and gamma-irradiation in neuroendocrine tumor cells
Source: PLoS One. 2017 May 25;12(5):e0178375. doi: 10.1371/journal.pone.0178375 (PMC5444855; doi:10.1371/journal.pone.0178375)

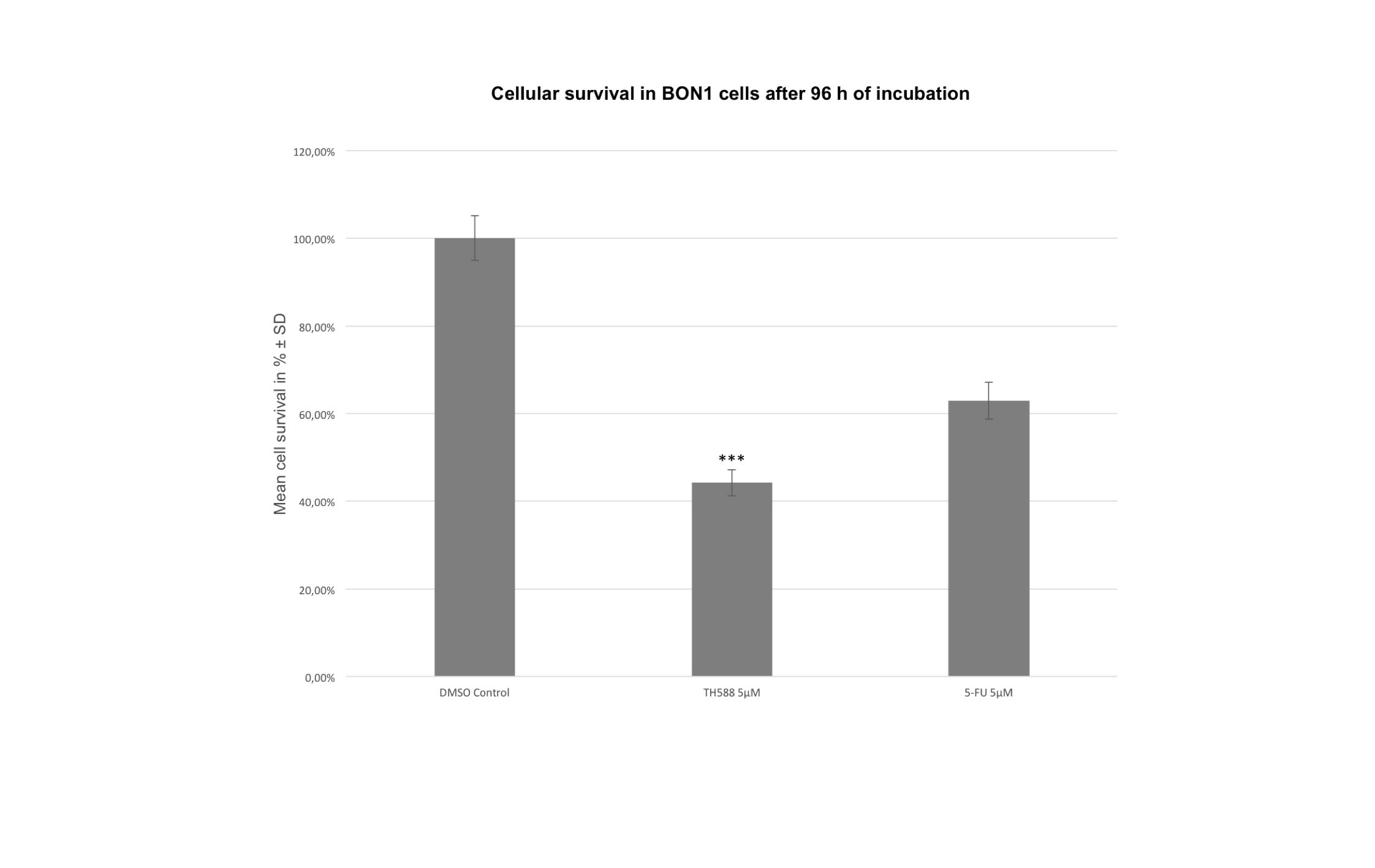

Supplement: S1 Fig — Effect of TH588 on cell survival. Human neuroendocrine pancreatic BON1 cells were incubated with TH588 (5 μM) or 5-FU (5 μM) for 96 h. The arithmetic means and standard deviation of at least three independent experiments are shown. Statistical significant different results in comparison to either single substance treatment are shown, considering p<0,05 = *; p<0,01 = **; p<0,001 = ***. (TIF) [file pone.0178375.s001.tif]

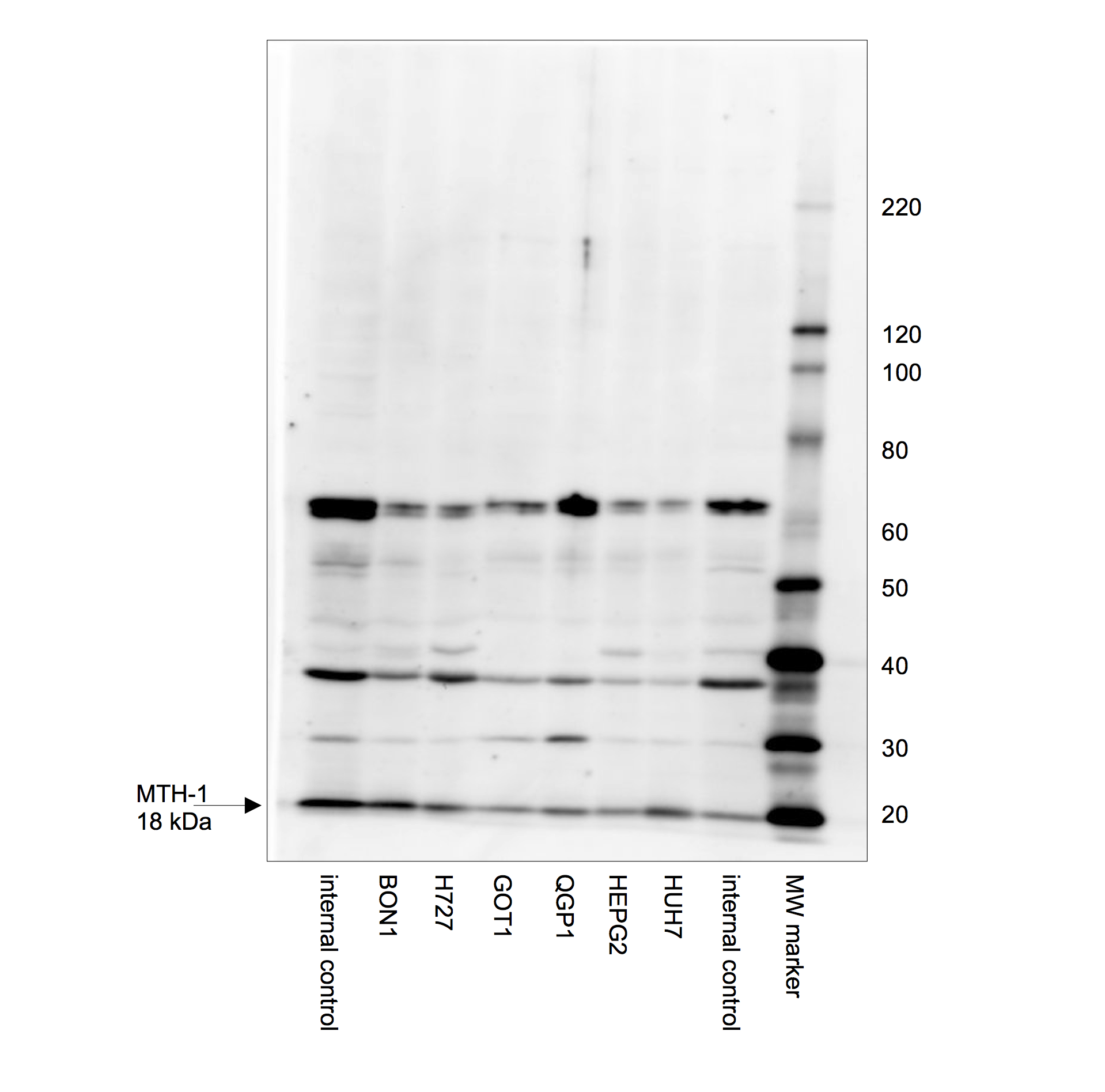

Supplement: S2 Fig — Basal expression of MTH1 in different neuroendocrine cell lines (BON1, H727, GOT1 and QGP1) and in HEPG2 and HUH7 cells. (TIF) [file pone.0178375.s002.tif]

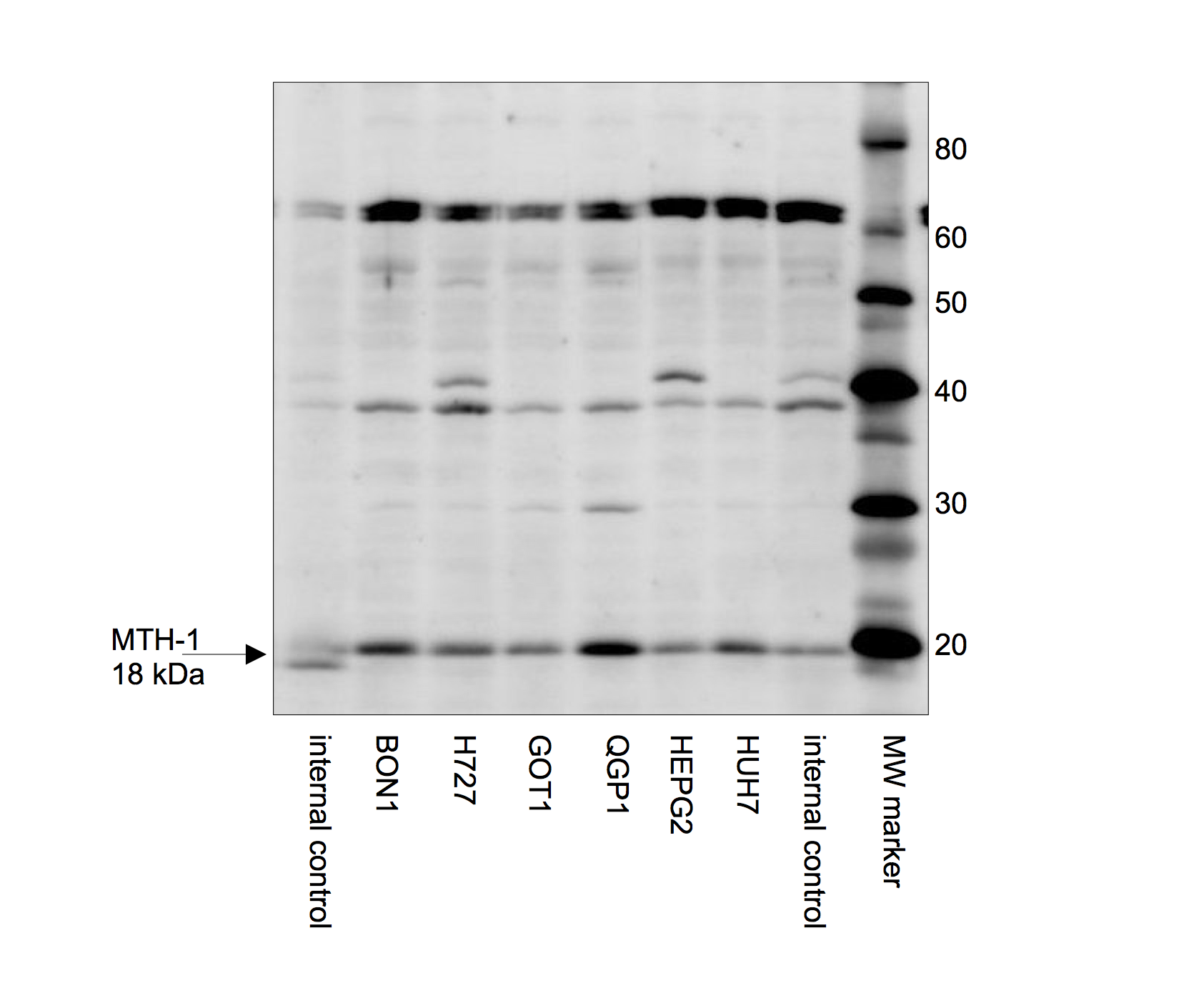

Supplement: S3 Fig — Basal expression of MTH1 in different neuroendocrine cell lines (BON1, H727, GOT1 and QGP1) and in HEPG2 and HUH7 cells. (TIF) [file pone.0178375.s003.tif]

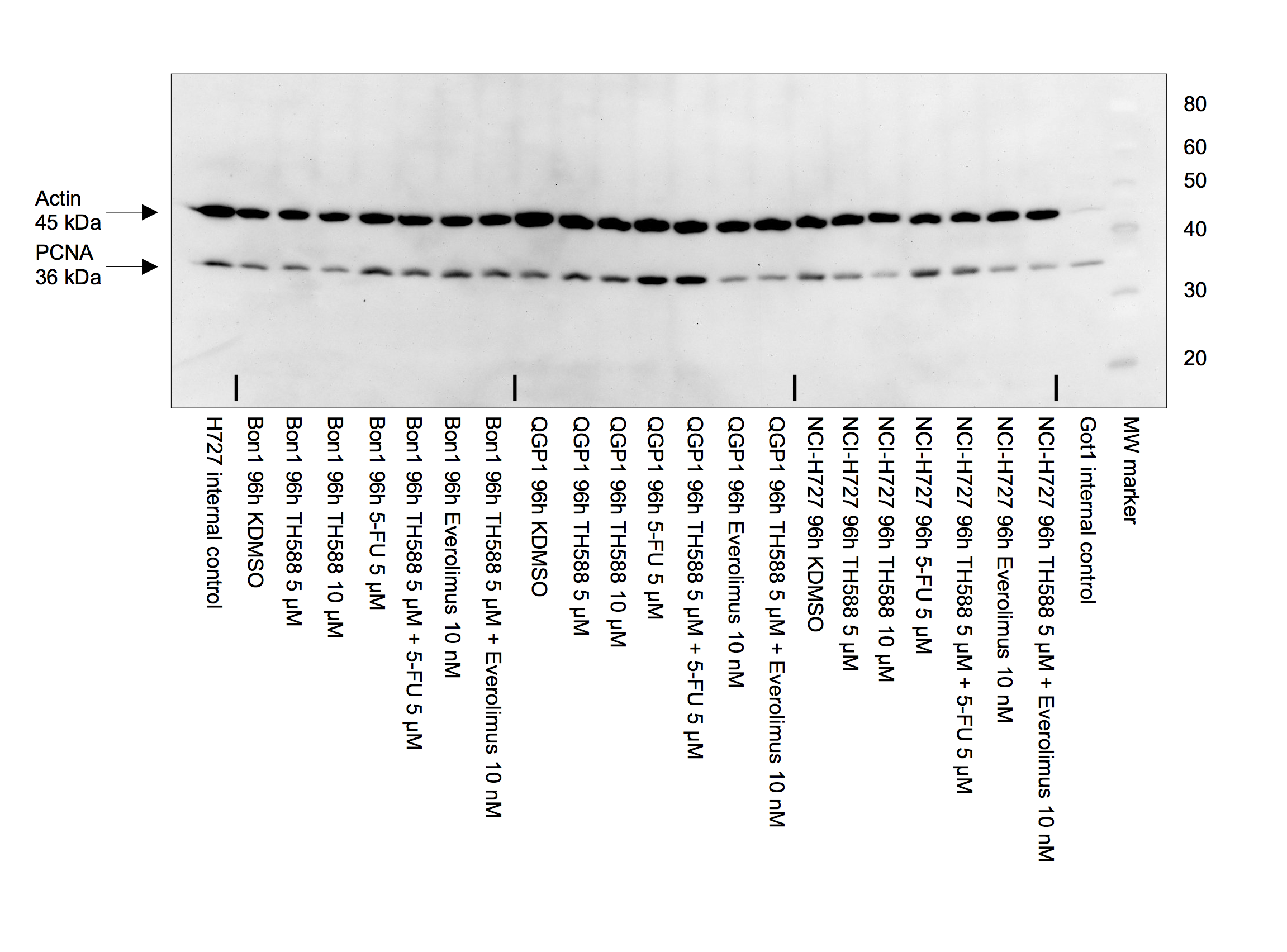

Supplement: S4 Fig — Expression of Actin and PCNA in neuroendocrine cell lines (BON1, H727 and QGP1) after 96 h of incubation with TH588 (5 μM or 10 μM) alone or in combination with 5FU (5 μM) or everolimus (10 nM). (TIF) [file pone.0178375.s004.tif]

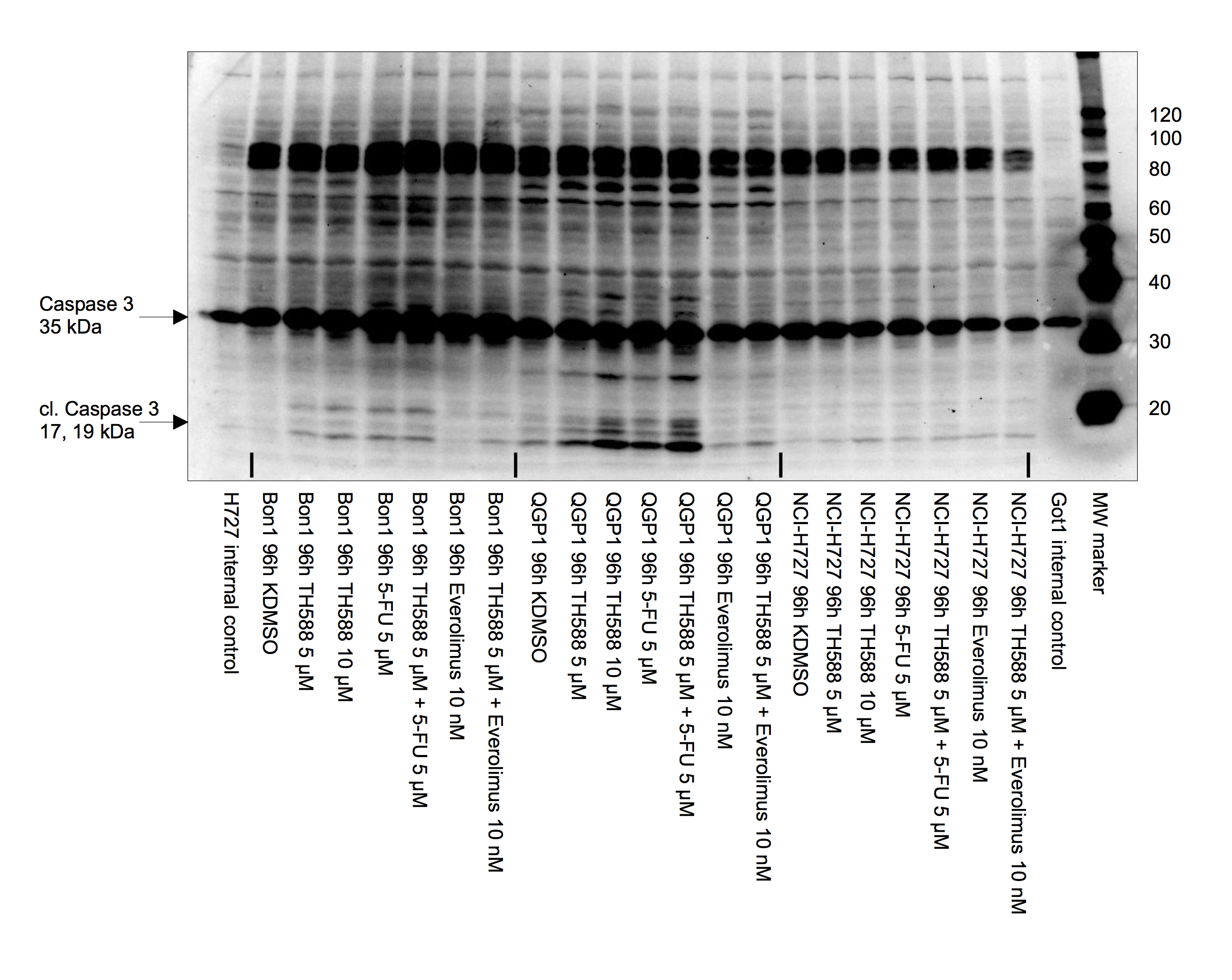

Supplement: S5 Fig — Expression of Caspase 3 and cleaved Caspase 3 in neuroendocrine cell lines (BON1, H727 and QGP1) after 96 h of incubation with TH588 (5 μM or 10 μM) alone or in combination with 5FU (5 μM) or everolimus (10 nM). (TIF) [file pone.0178375.s005.tif]

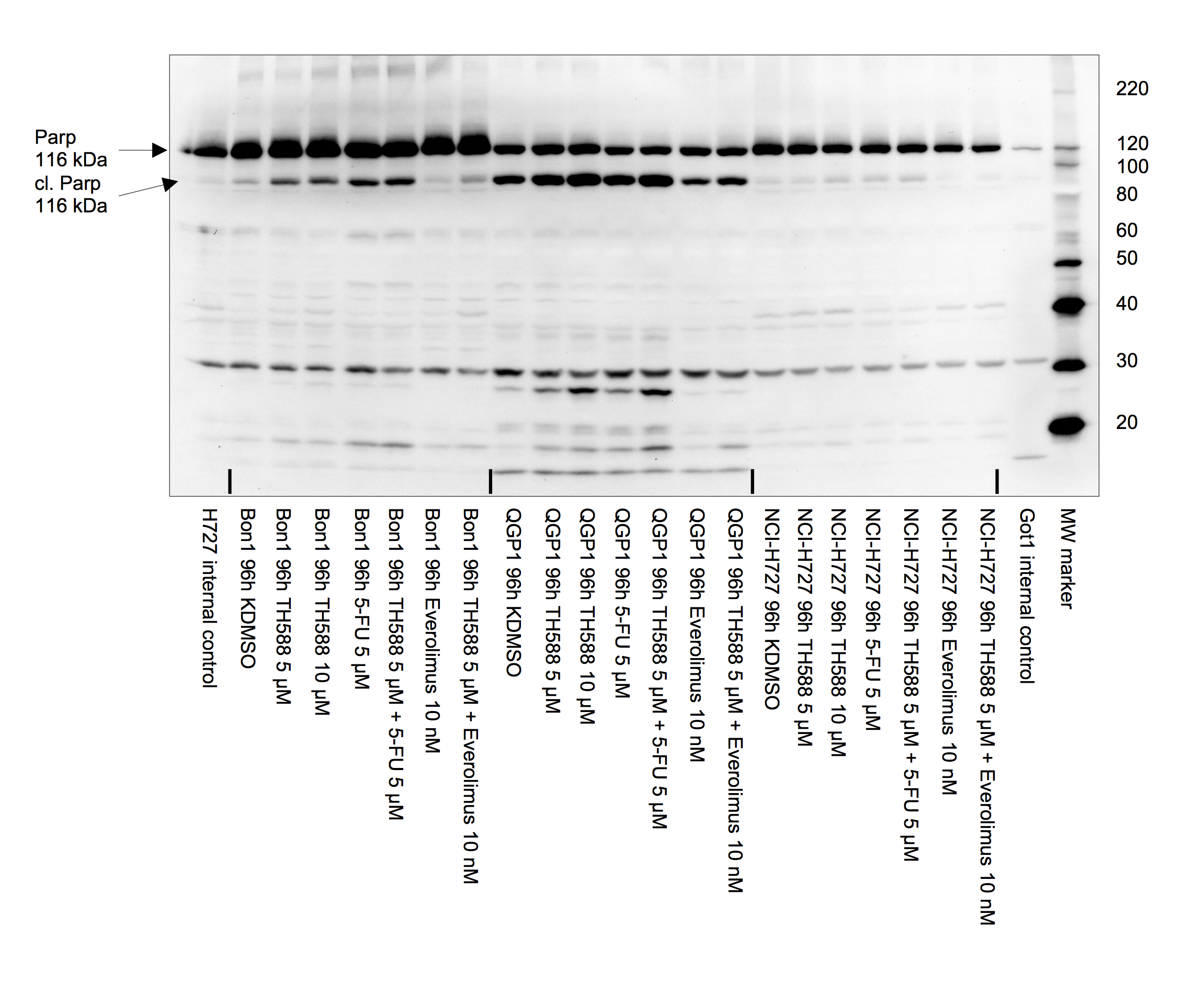

Supplement: S6 Fig — Expression of PARP and cleaved PARP in neuroendocrine cell lines (BON1, H727 and QGP1) after 96 h of incubation with TH588 (5 μM or 10 μM) alone or in combination with 5FU (5 μM) or everolimus (10 nM). (TIF) [file pone.0178375.s006.tif]

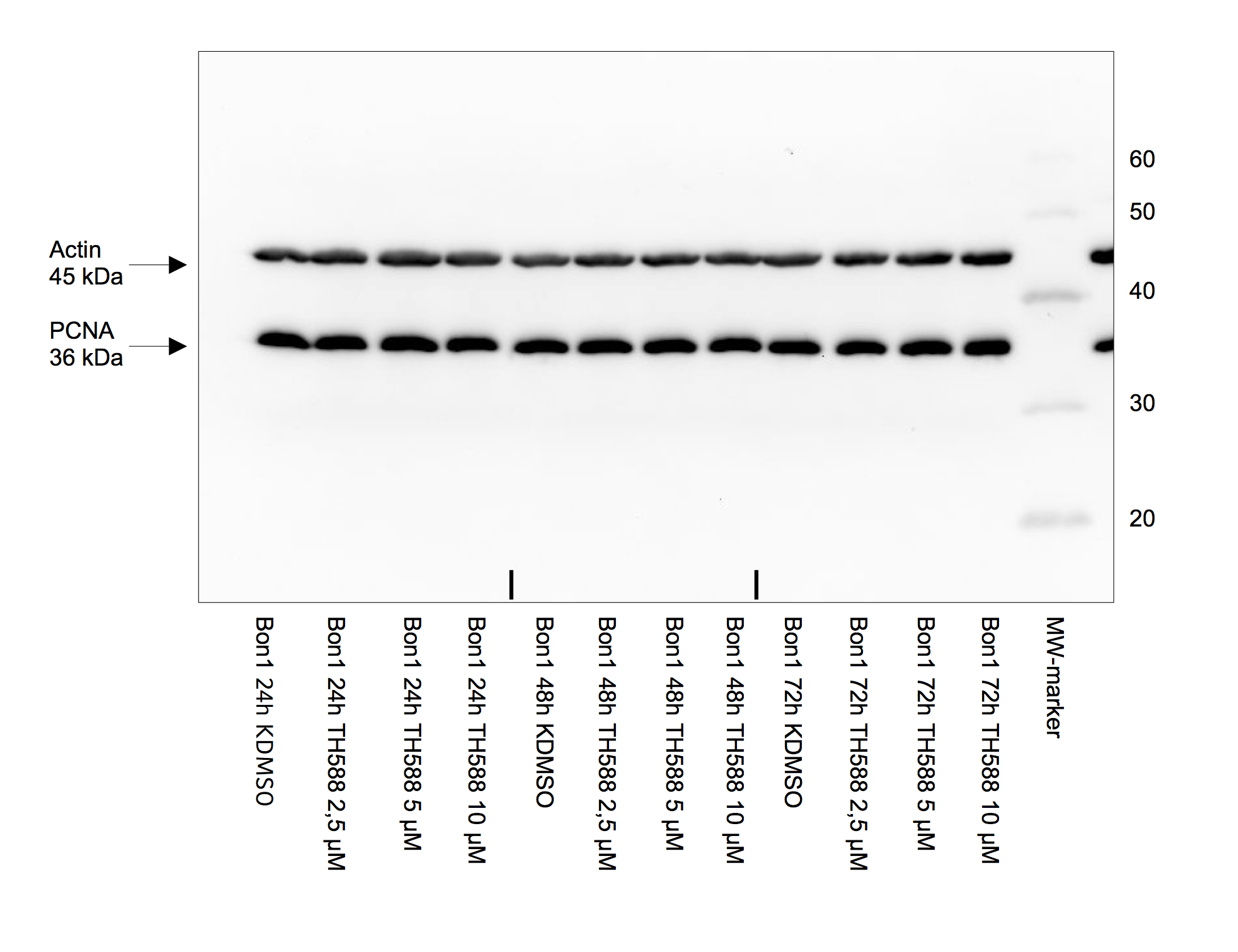

Supplement: S7 Fig — Expression of Actin and PCNA in BON1 cells after 24 h, 48 h and 72 h of incubation with TH588 (2,5 μM, 5 μM or 10 μM). (TIF) [file pone.0178375.s007.tif]

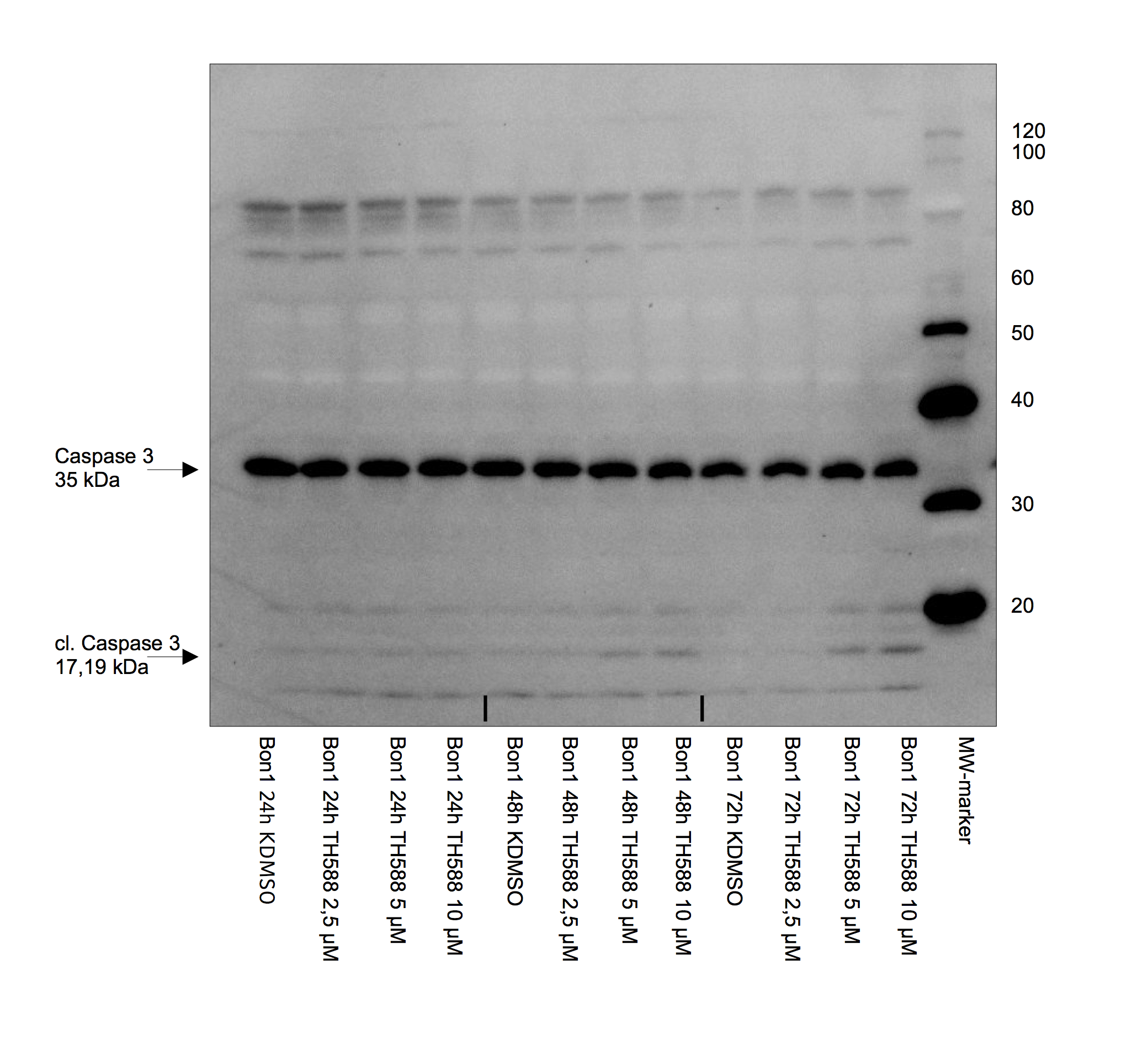

Supplement: S8 Fig — Expression of Caspase 3 and cleaved Caspase 3 in BON1 cells after 24 h, 48 h and 72 h of incubation with TH588 (2,5 μM, 5 μM or 10 μM). (TIF) [file pone.0178375.s008.tif]

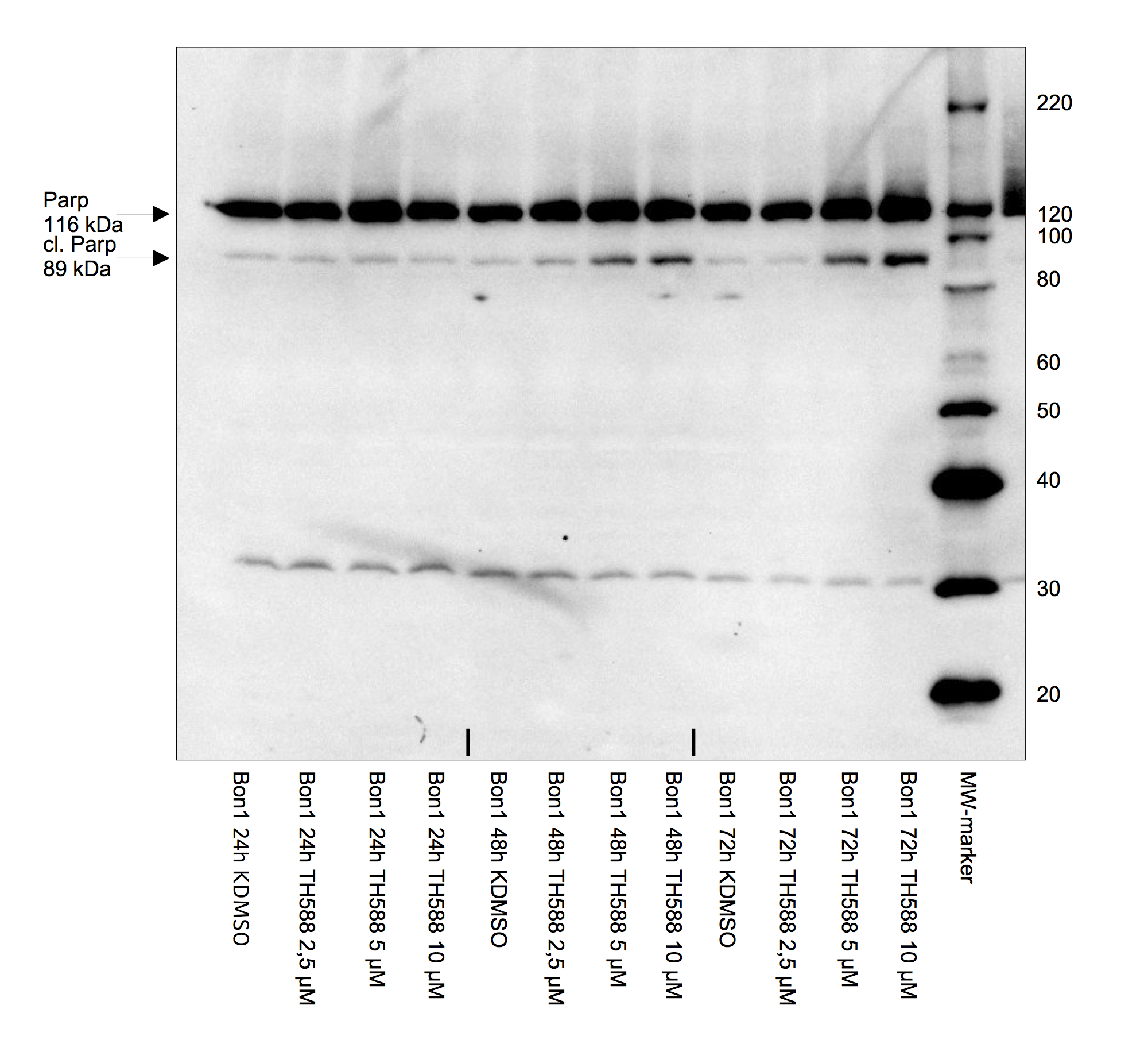

Supplement: S9 Fig — Expression of PARP and cleaved PARP in BON1 cells after 24 h, 48 h and 72 h of incubation with TH588 (2,5 μM, 5 μM or 10 μM). (TIF) [file pone.0178375.s009.tif]

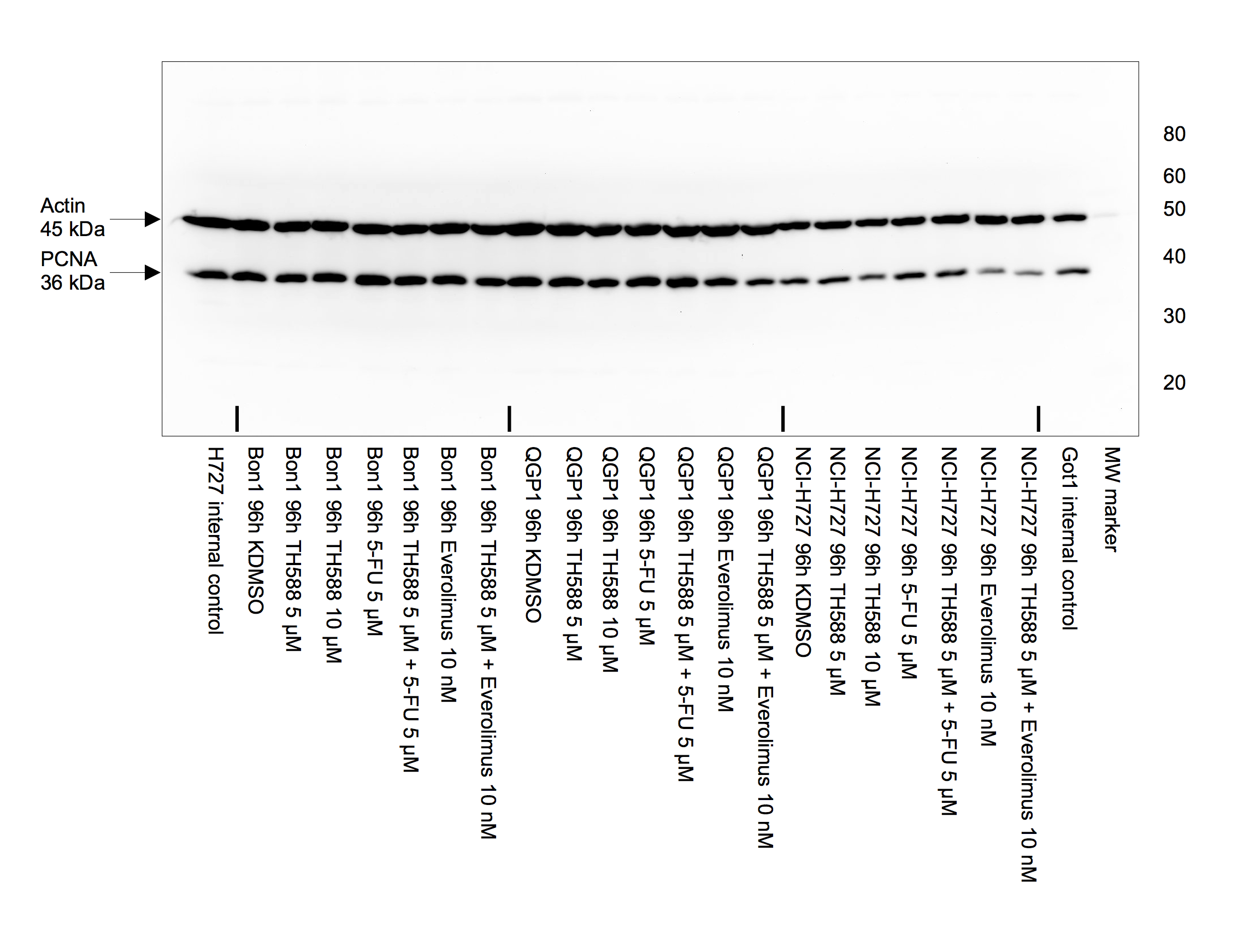

Supplement: S10 Fig — Expression of Actin and PCNA in neuroendocrine cell lines (BON1, H727 and QGP1) after 96 h of incubation with TH588 (5 μM or 10 μM) alone or in combination with 5FU (5 μM) or everolimus (10 nM). (TIF) [file pone.0178375.s010.tif]

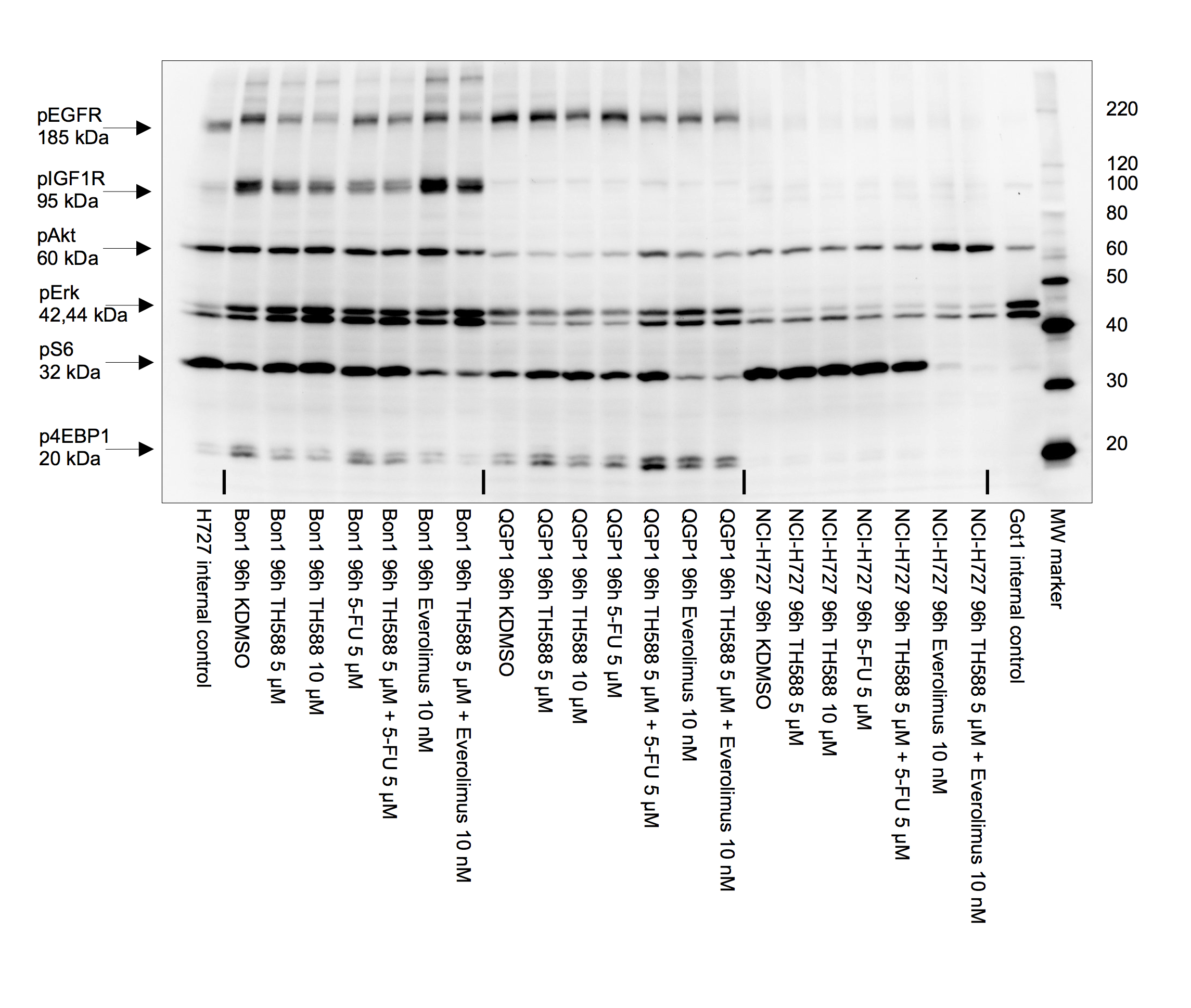

Supplement: S11 Fig — Expression of pEGFR, pIGFR, pAkt, pErk, pS6 and p4EBP1 in neuroendocrine cell lines (BON1, H727 and QGP1) after 96 h of incubation with TH588 (5 μM or 10 μM) alone or in combination with 5FU (5 μM) or everolimus (10 nM). (TIF) [file pone.0178375.s011.tif]

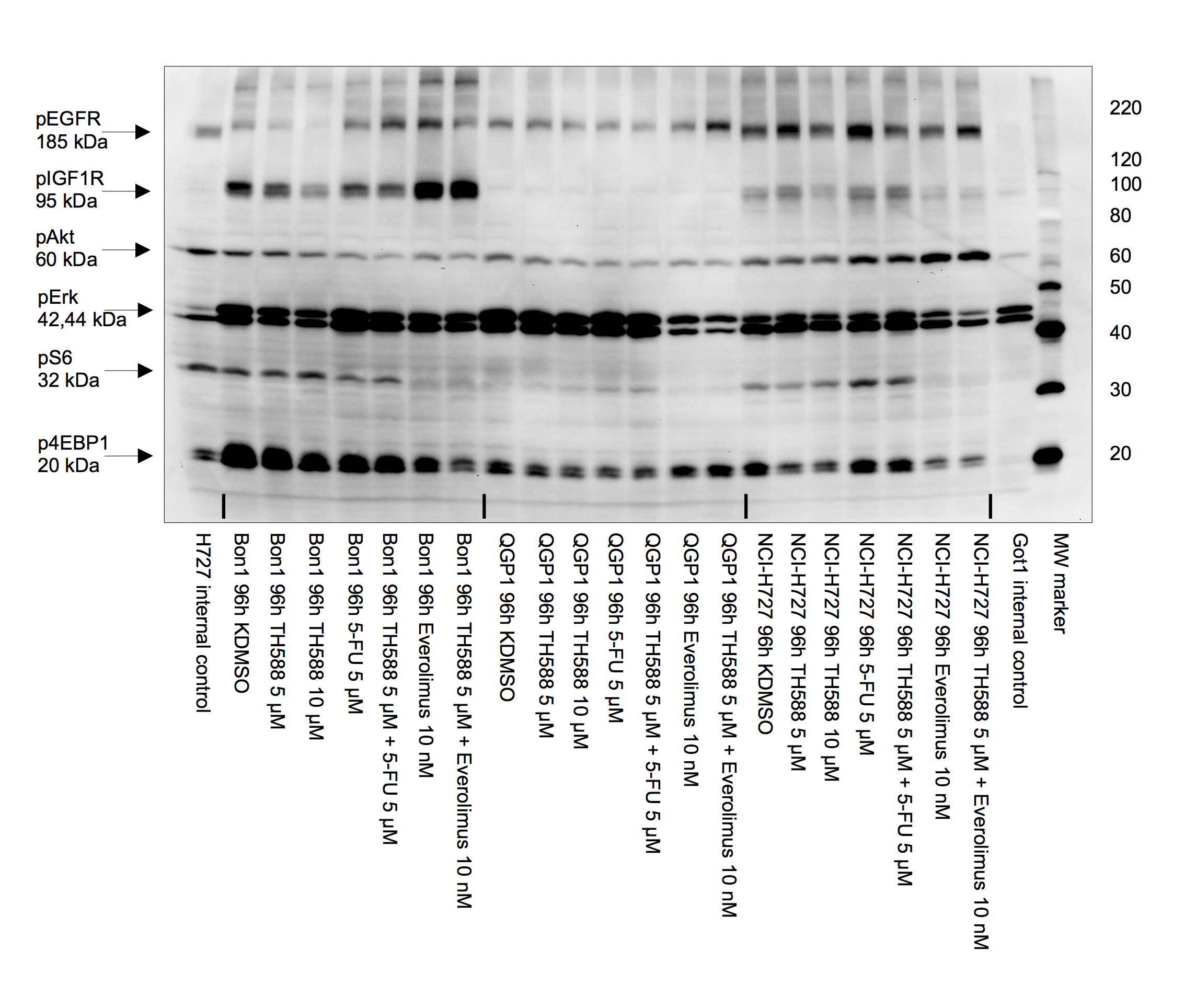

Supplement: S12 Fig — Expression of pEGFR, pIGFR, pAkt, pErk, pS6 and p4EBP1 in neuroendocrine cell lines (BON1, H727 and QGP1) after 96 h of incubation with TH588 (5 μM or 10 μM) alone or in combination with 5FU (5 μM) or everolimus (10 nM). (TIF) [file pone.0178375.s012.tif]

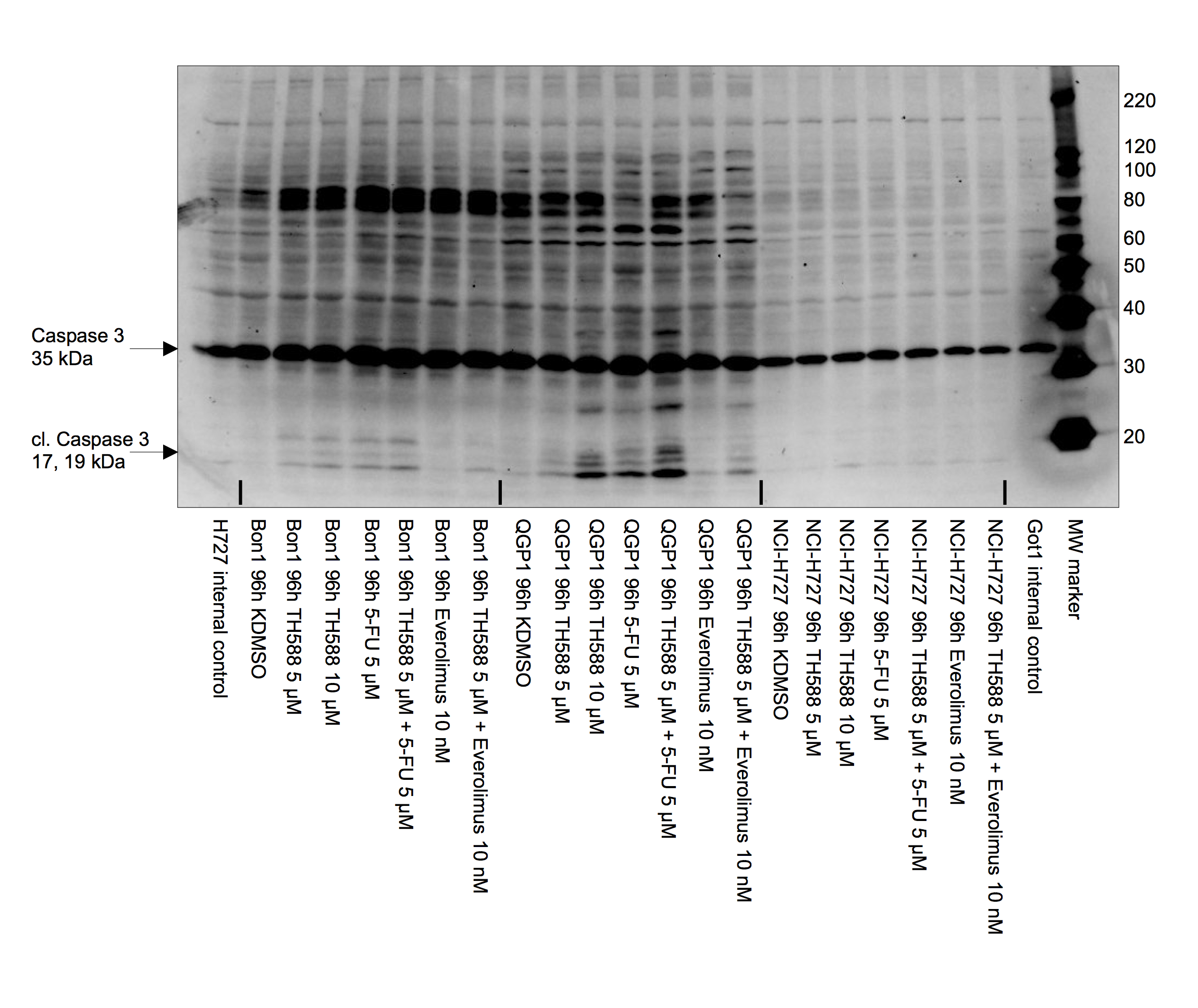

Supplement: S13 Fig — Expression of Caspase 3 and cleaved Caspase 3 in neuroendocrine cell lines (BON1, H727 and QGP1) after 96 h of incubation with TH588 (5 μM or 10 μM) alone or in combination with 5FU (5 μM) or everolimus (10 nM). (TIF) [file pone.0178375.s013.tif]

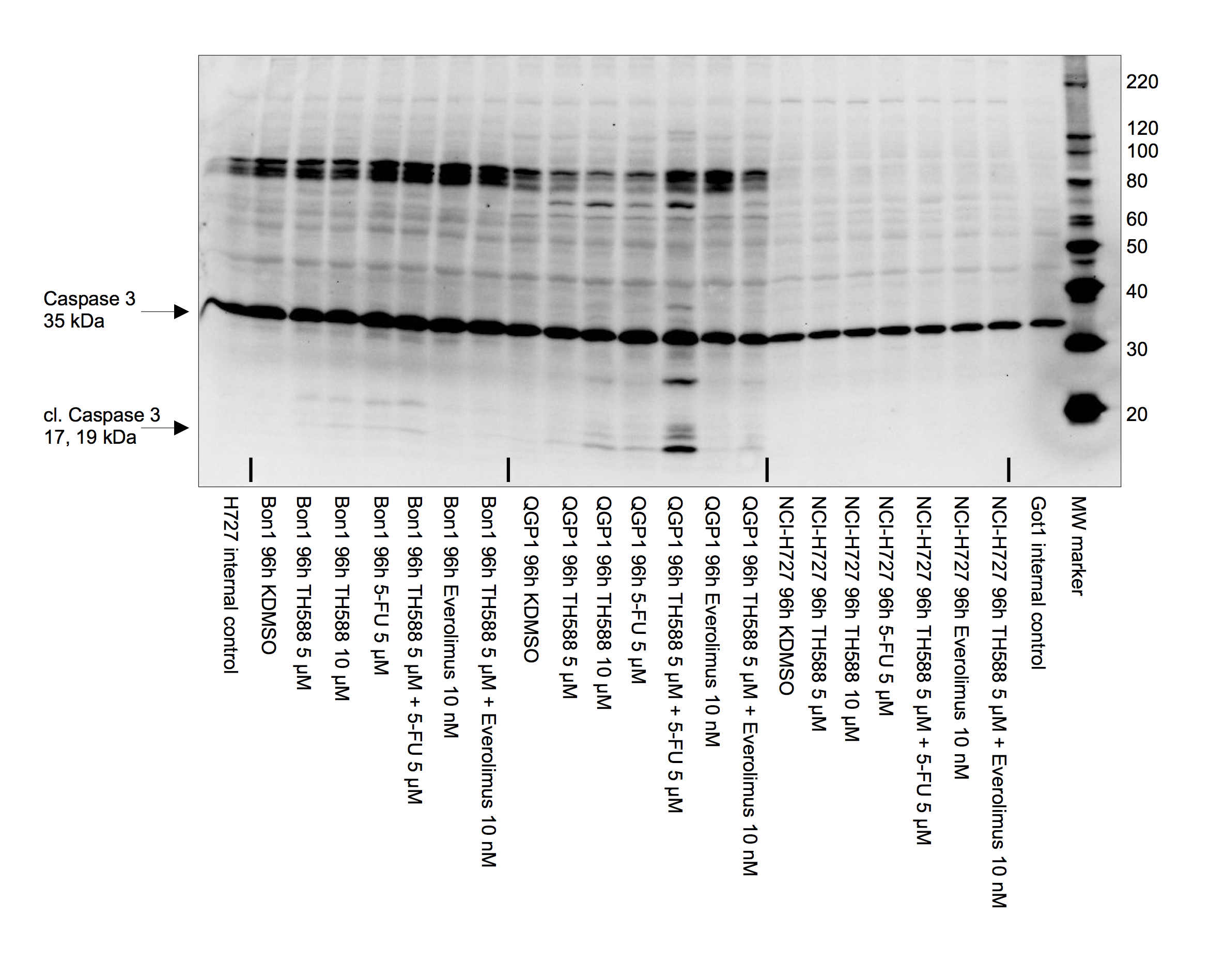

Supplement: S14 Fig — Expression of Caspase 3 and cleaved Caspase 3 in neuroendocrine cell lines (BON1, H727 and QGP1) after 96 h of incubation with TH588 (5 μM or 10 μM) alone or in combination with 5FU (5 μM) or everolimus (10 nM). (TIF) [file pone.0178375.s014.tif]

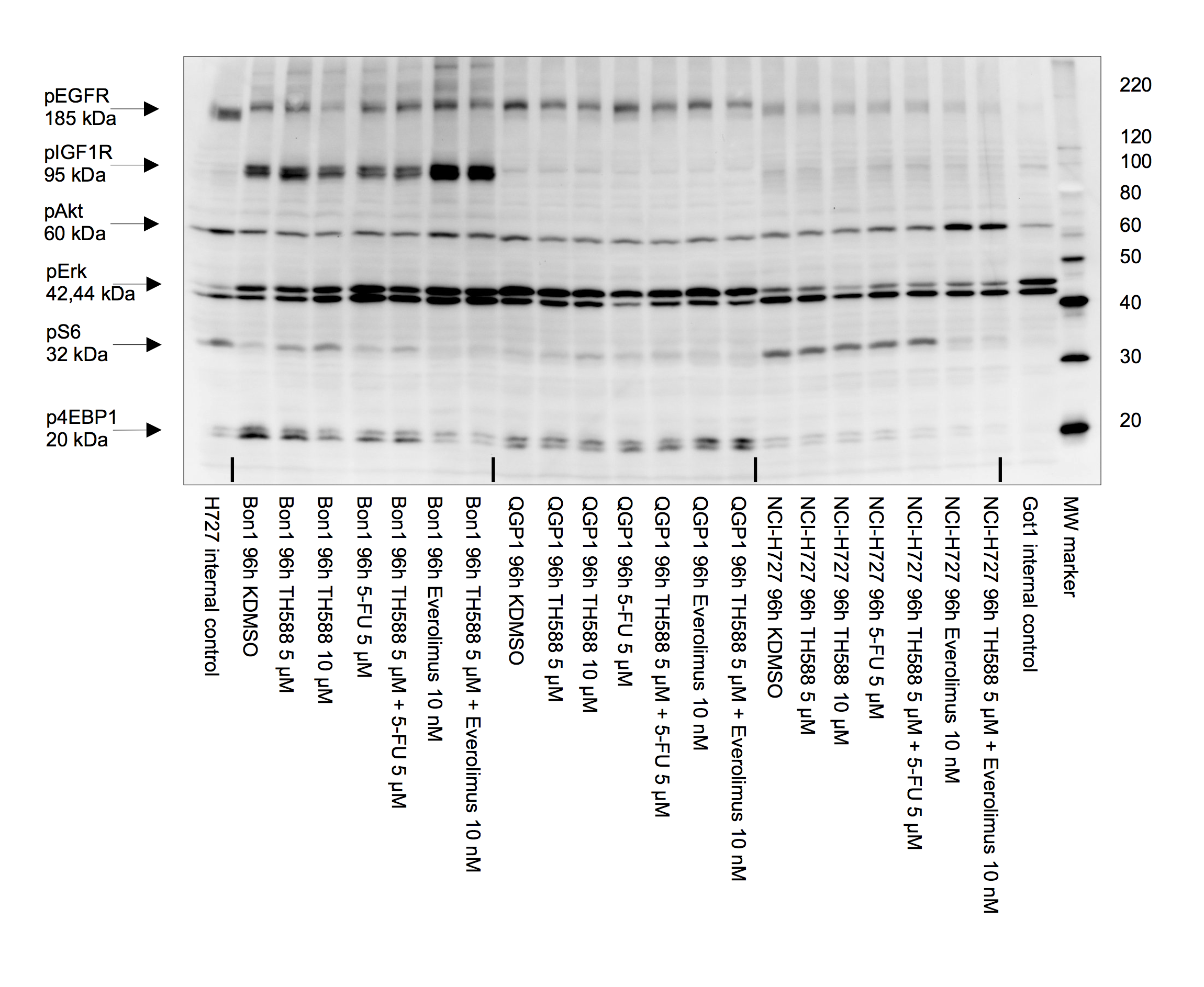

Supplement: S16 Fig — Expression of pEGFR, pIGFR, pAkt, pErk, pS6 and p4EBP1 in neuroendocrine cell lines (BON1, H727 and QGP1) after 96 h of incubation with TH588 (5 μM or 10 μM) alone or in combination with 5FU (5 μM) or everolimus (10 nM). (TIF) [file pone.0178375.s016.tif]

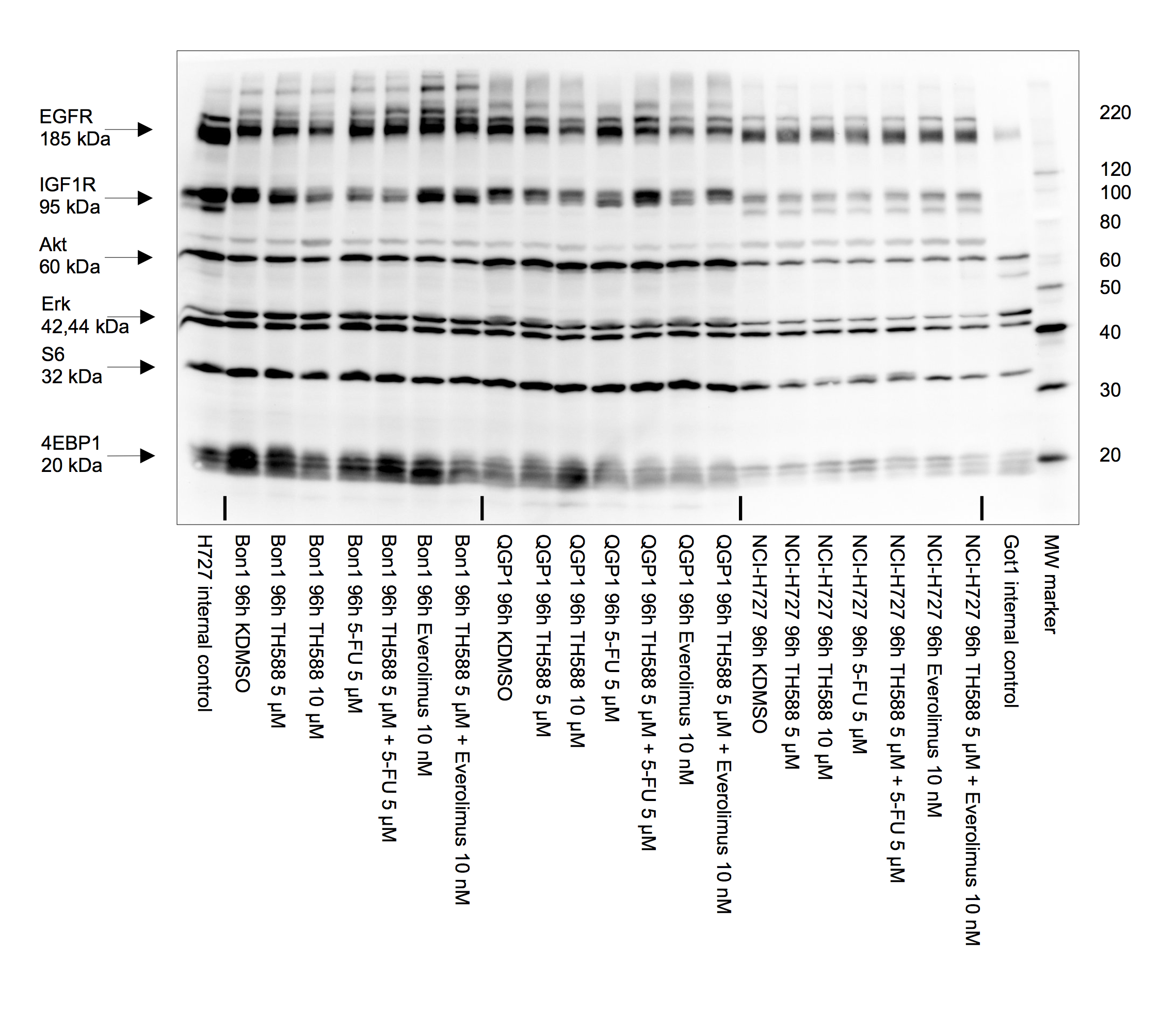

Supplement: S19 Fig — Expression of EGFR, IGFR, Akt, Erk, S6 and 4EBP1 in neuroendocrine cell lines (BON1, H727 and QGP1) after 96 h of incubation with TH588 (5 μM or 10 μM) alone or in combination with 5FU (5 μM) or everolimus (10 nM). (TIF) [file pone.0178375.s019.tif]

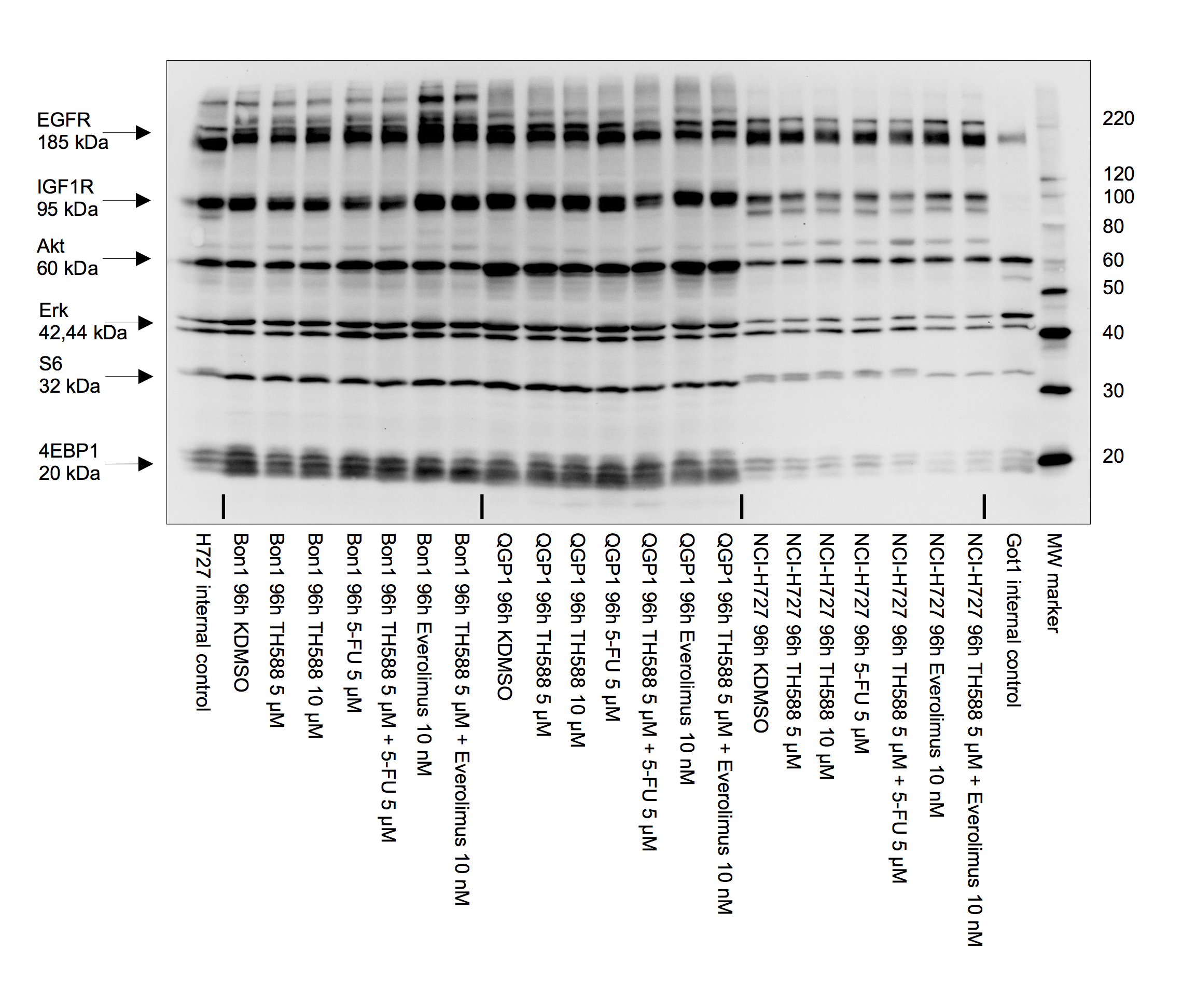

Supplement: S20 Fig — Expression of EGFR, IGFR, Akt, Erk, S6 and 4EBP1 in neuroendocrine cell lines (BON1, H727 and QGP1) after 96 h of incubation with TH588 (5 μM or 10 μM) alone or in combination with 5FU (5 μM) or everolimus (10 nM). (TIF) [file pone.0178375.s020.tif]

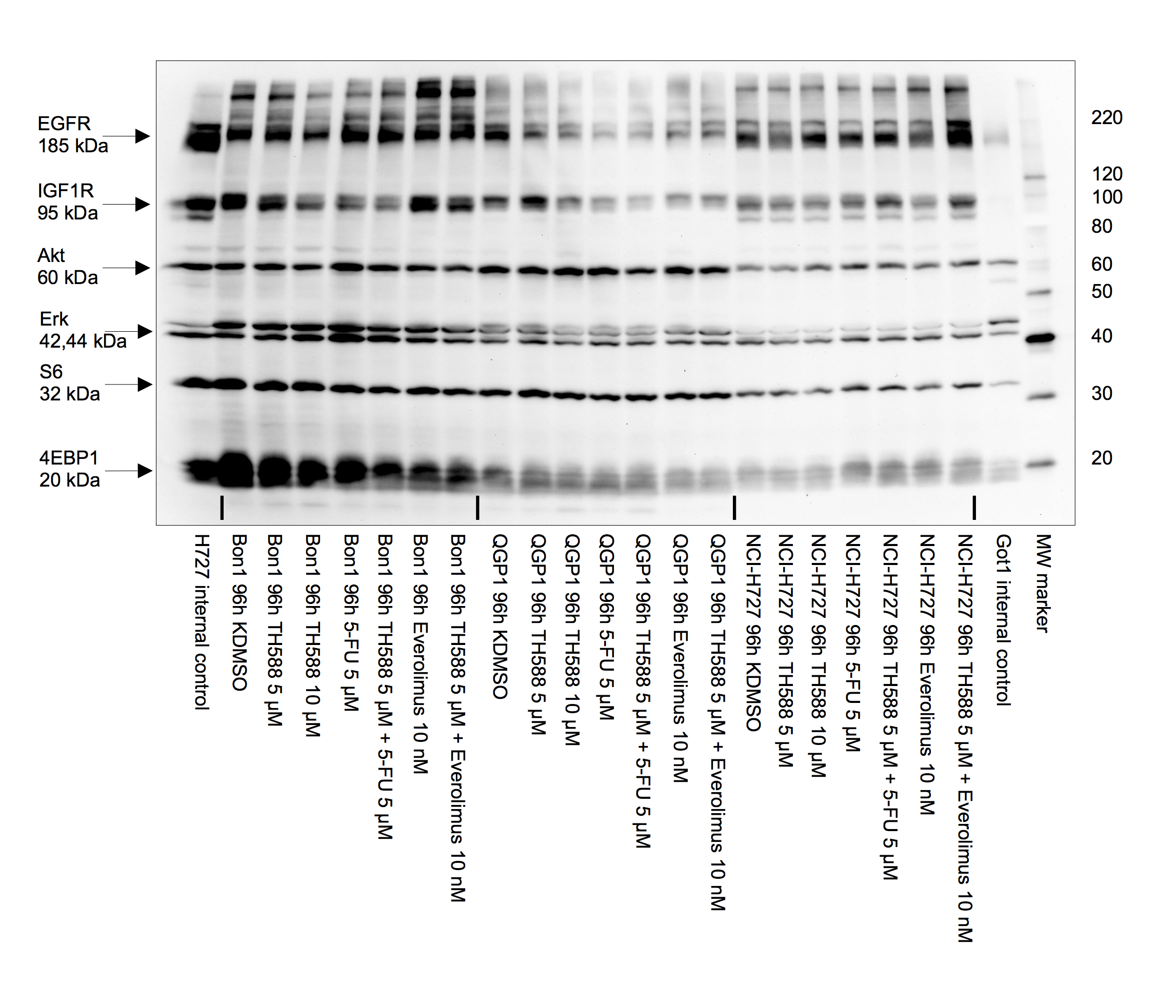

Supplement: S21 Fig — Expression of EGFR, IGFR, Akt, Erk, S6 and 4EBP1 in neuroendocrine cell lines (BON1, H727 and QGP1) after 96 h of incubation with TH588 (5 μM or 10 μM) alone or in combination with 5FU (5 μM) or everolimus (10 nM). (TIF) [file pone.0178375.s021.tif]

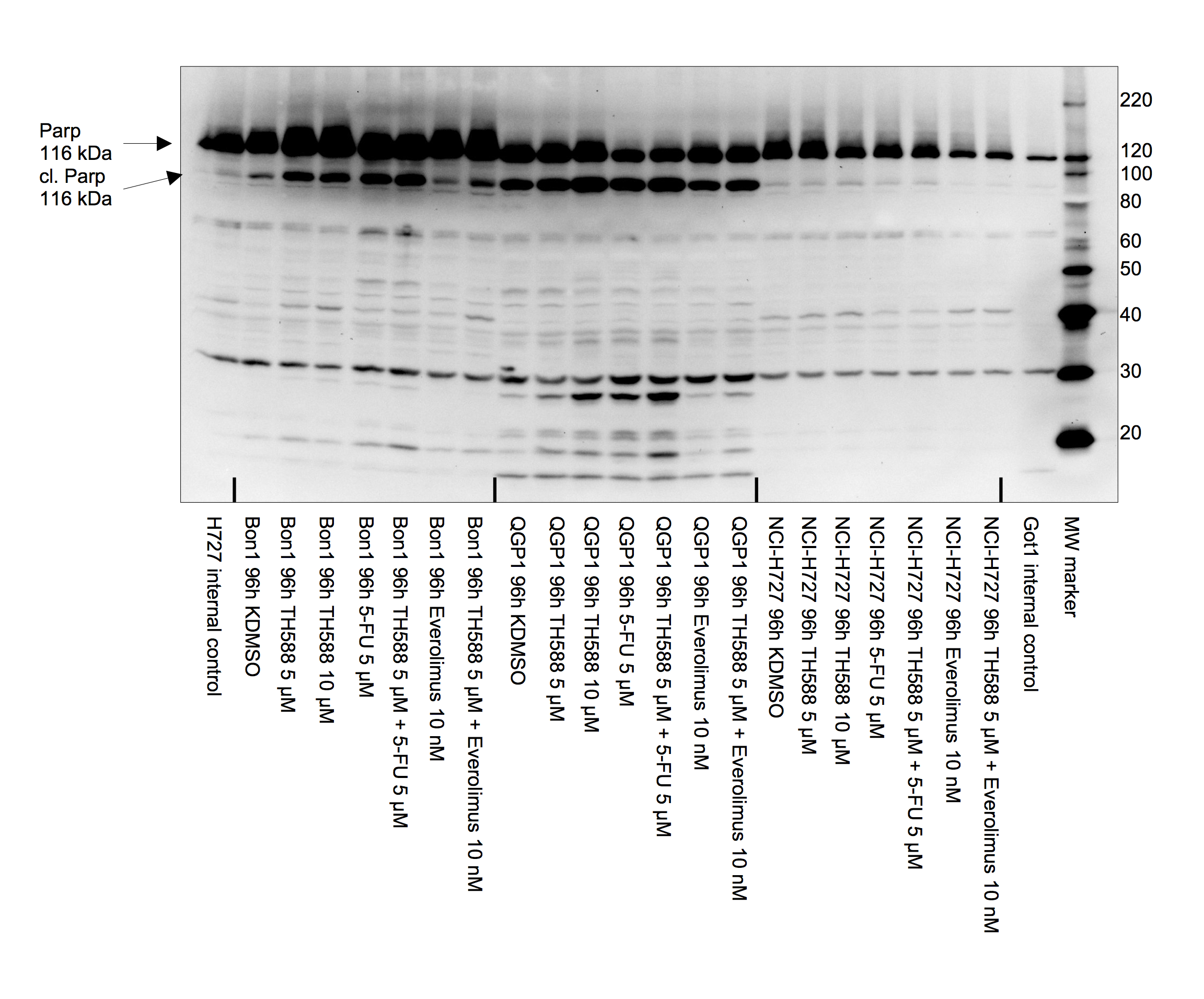

Supplement: S22 Fig — Expression of PARP and cleaved PARP in neuroendocrine cell lines (BON1, H727 and QGP1) after 96 h of incubation with TH588 (5 μM or 10 μM) alone or in combination with 5FU (5 μM) or everolimus (10 nM). (TIF) [file pone.0178375.s022.tif]

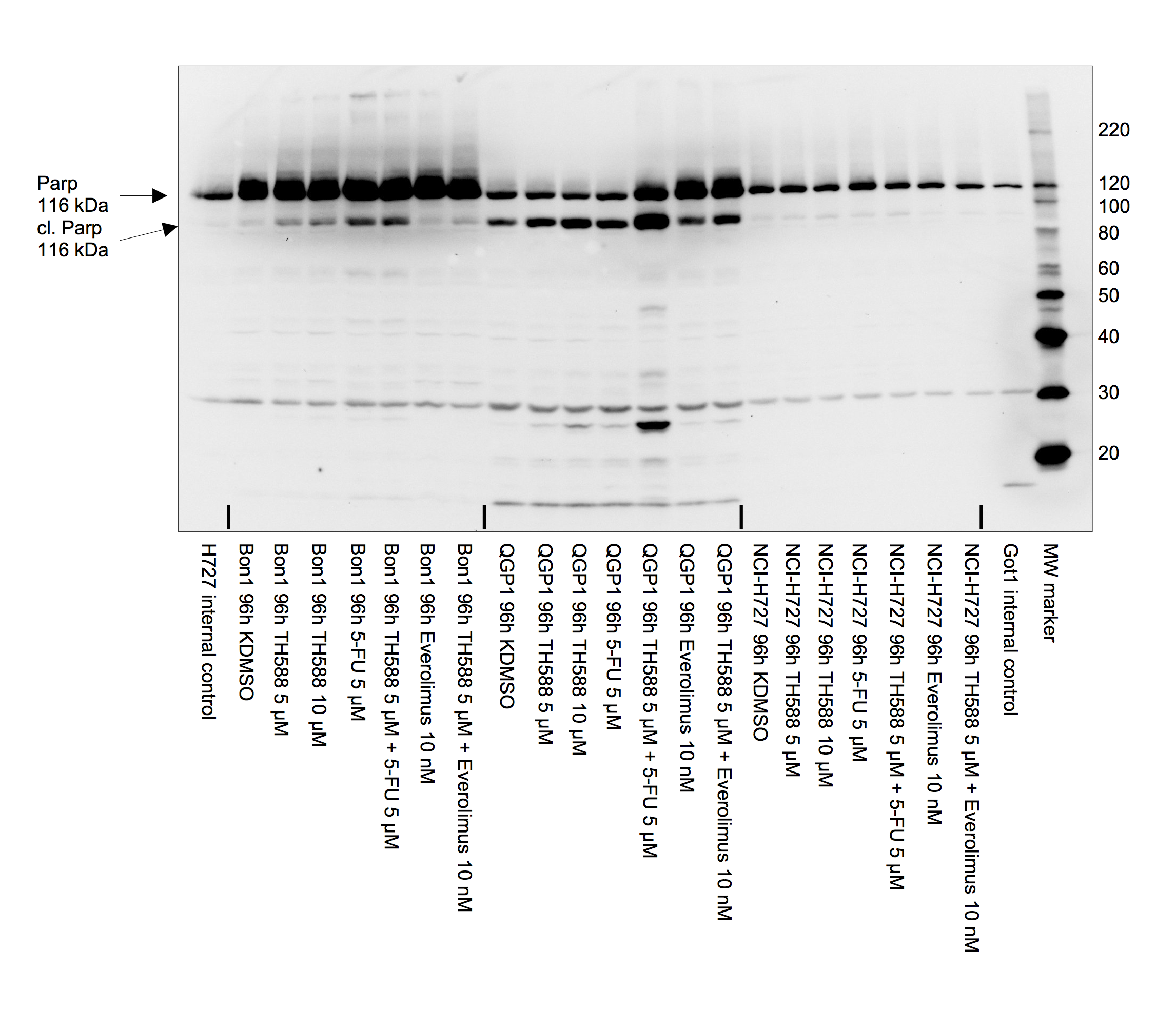

Supplement: S23 Fig — Expression of PARP and cleaved PARP in neuroendocrine cell lines (BON1, H727 and QGP1) after 96 h of incubation with TH588 (5 μM or 10 μM) alone or in combination with 5FU (5 μM) or everolimus (10 nM). (TIF) [file pone.0178375.s023.tif]

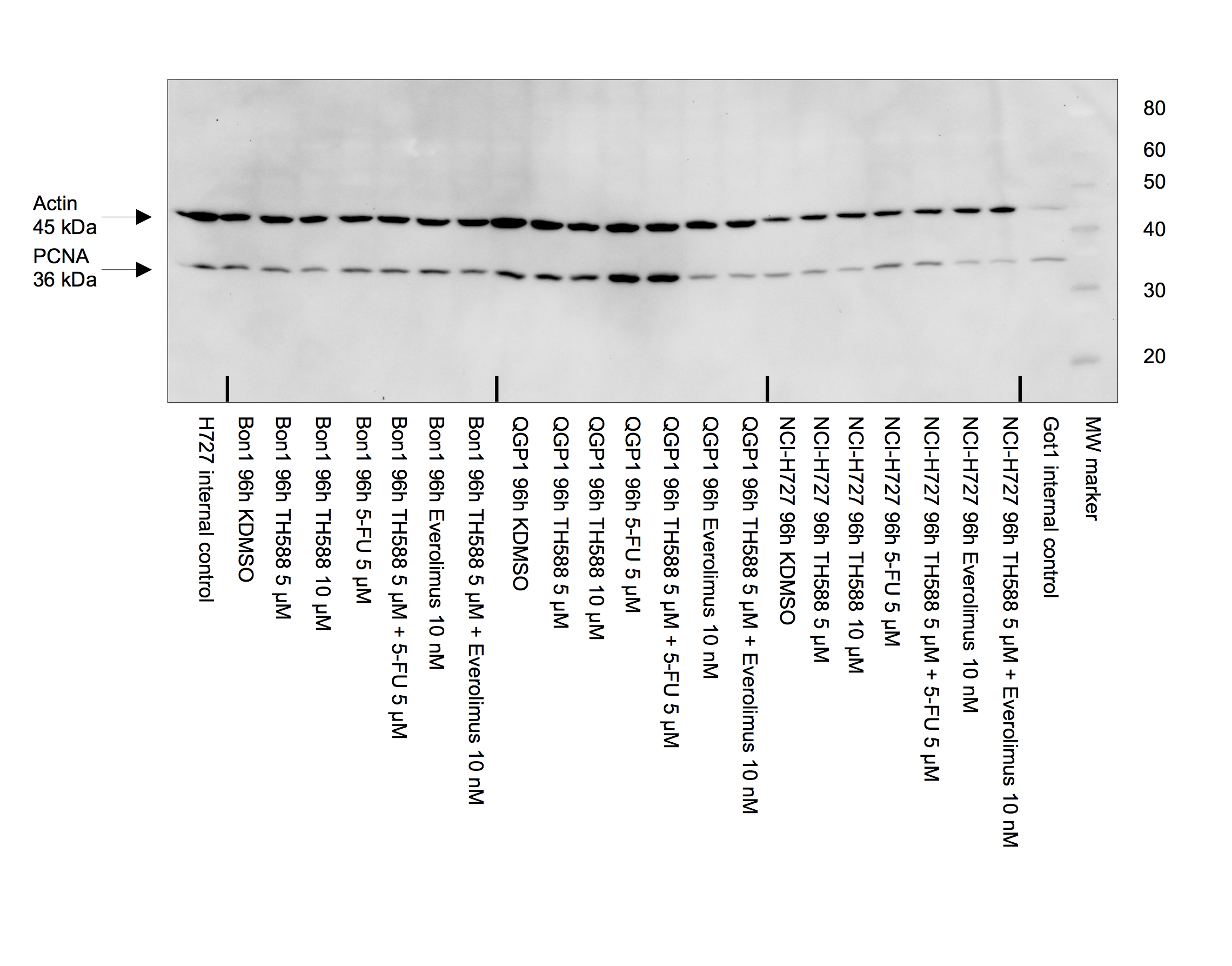

Supplement: S25 Fig — Expression of Actin and cleaved PCNA in neuroendocrine cell lines (BON1, H727 and QGP1) after 96 h of incubation with TH588 (5 μM or 10 μM) alone or in combination with 5FU (5 μM) or everolimus (10 nM). (TIF) [file pone.0178375.s025.tif]

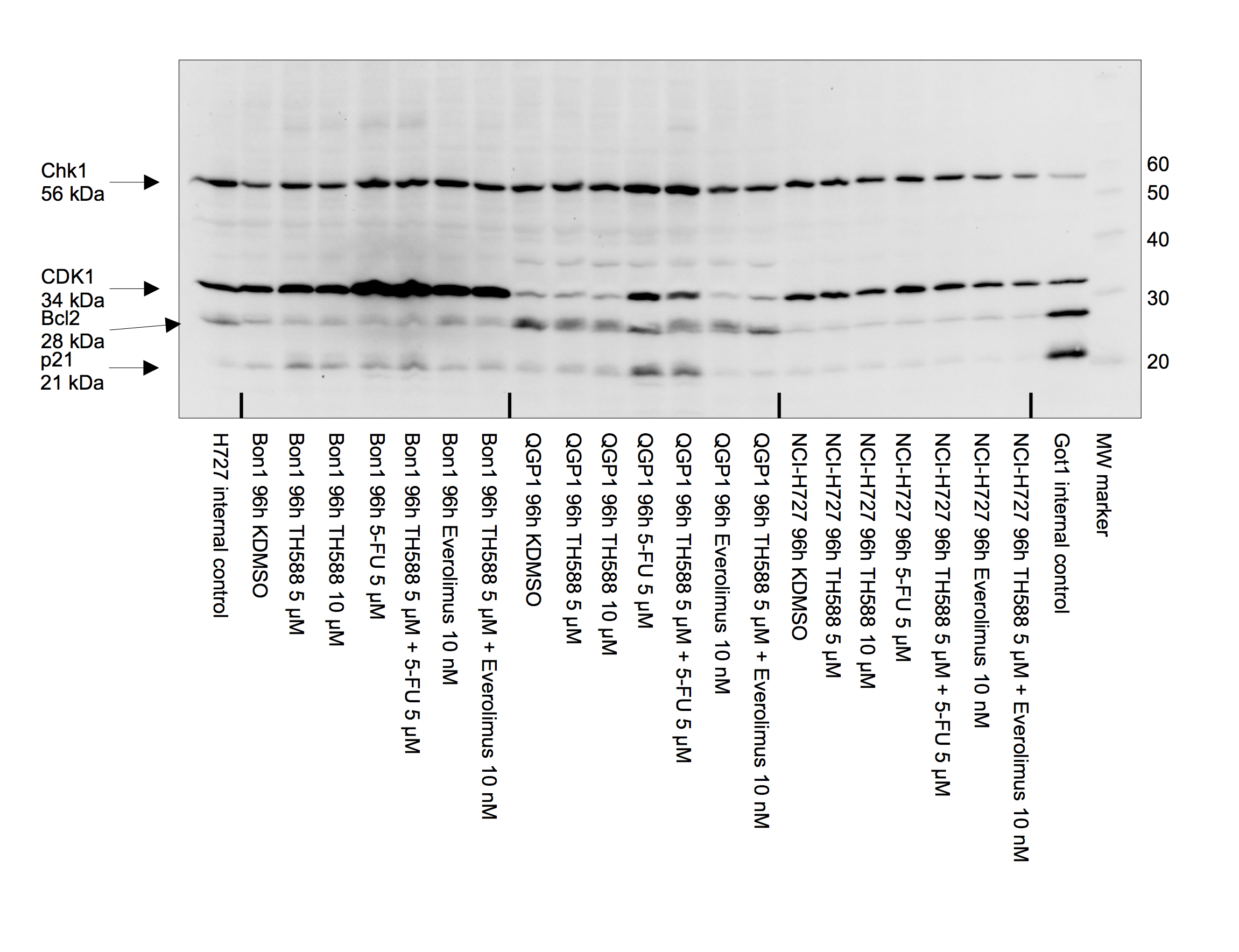

Supplement: S28 Fig — Expression of Chk1, CDK1, Bcl2 and p21 in neuroendocrine cell lines (BON1, H727 and QGP1) after 96 h of incubation with TH588 (5 μM or 10 μM) alone or in combination with 5FU (5 μM) or everolimus (10 nM). (TIF) [file pone.0178375.s028.tif]

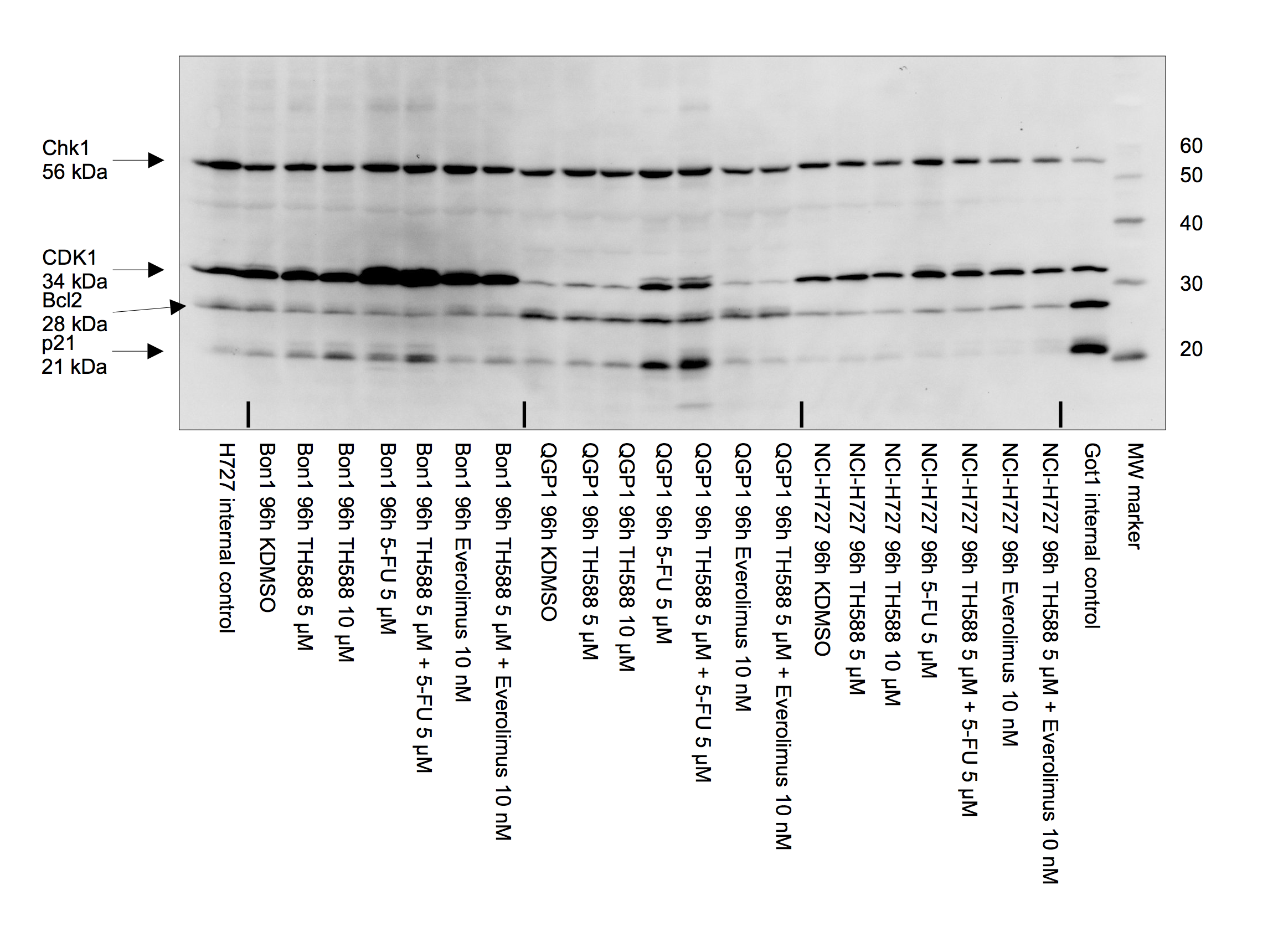

Supplement: S29 Fig — Expression of Chk1, CDK1, Bcl2 and p21 in neuroendocrine cell lines (BON1, H727 and QGP1) after 96 h of incubation with TH588 (5 μM or 10 μM) alone or in combination with 5FU (5 μM) or everolimus (10 nM). (TIF) [file pone.0178375.s029.tif]

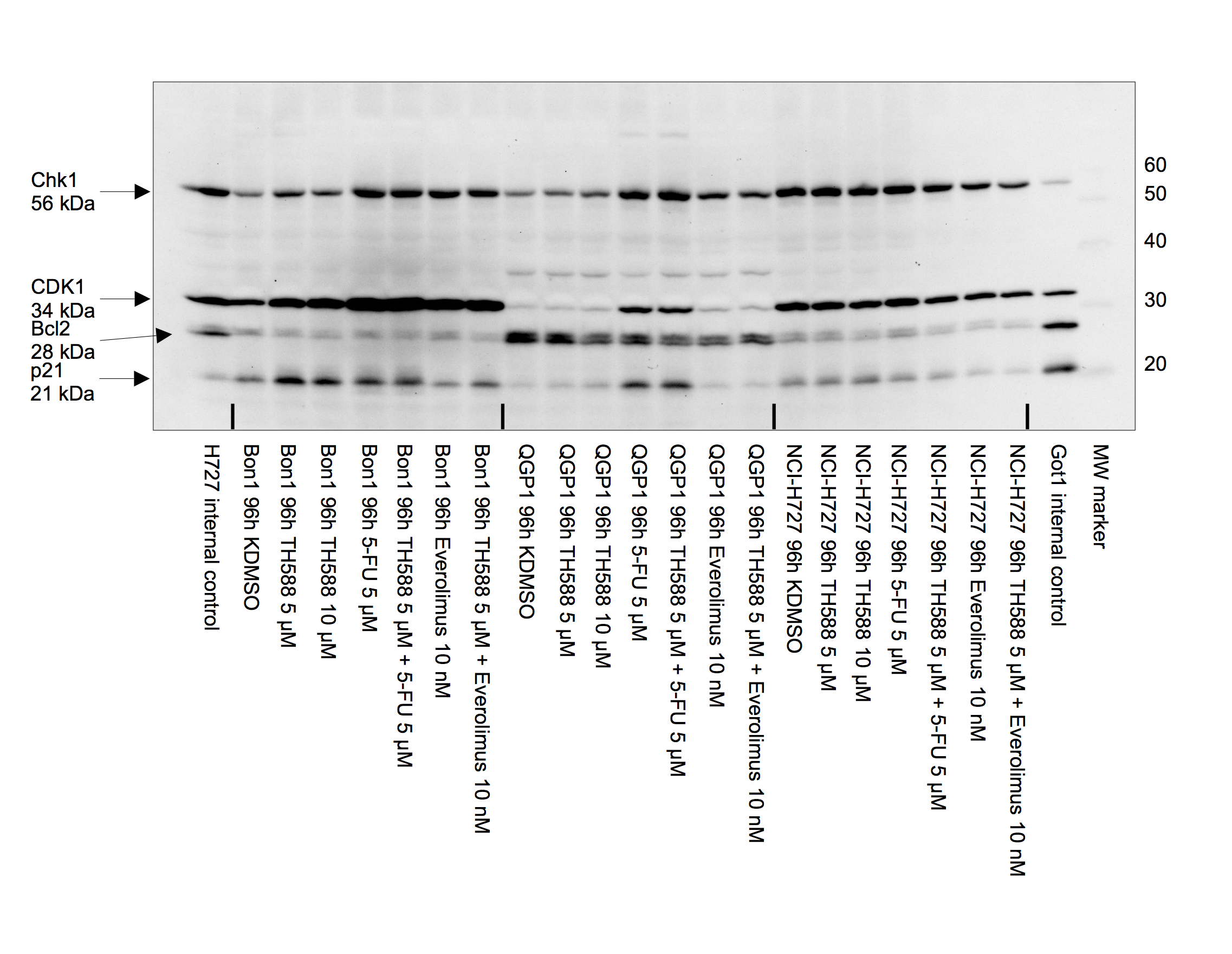

Supplement: S30 Fig — Expression of Chk1, CDK1, Bcl2 and p21 in neuroendocrine cell lines (BON1, H727 and QGP1) after 96 h of incubation with TH588 (5 μM or 10 μM) alone or in combination with 5FU (5 μM) or everolimus (10 nM). (TIF) [file pone.0178375.s030.tif]

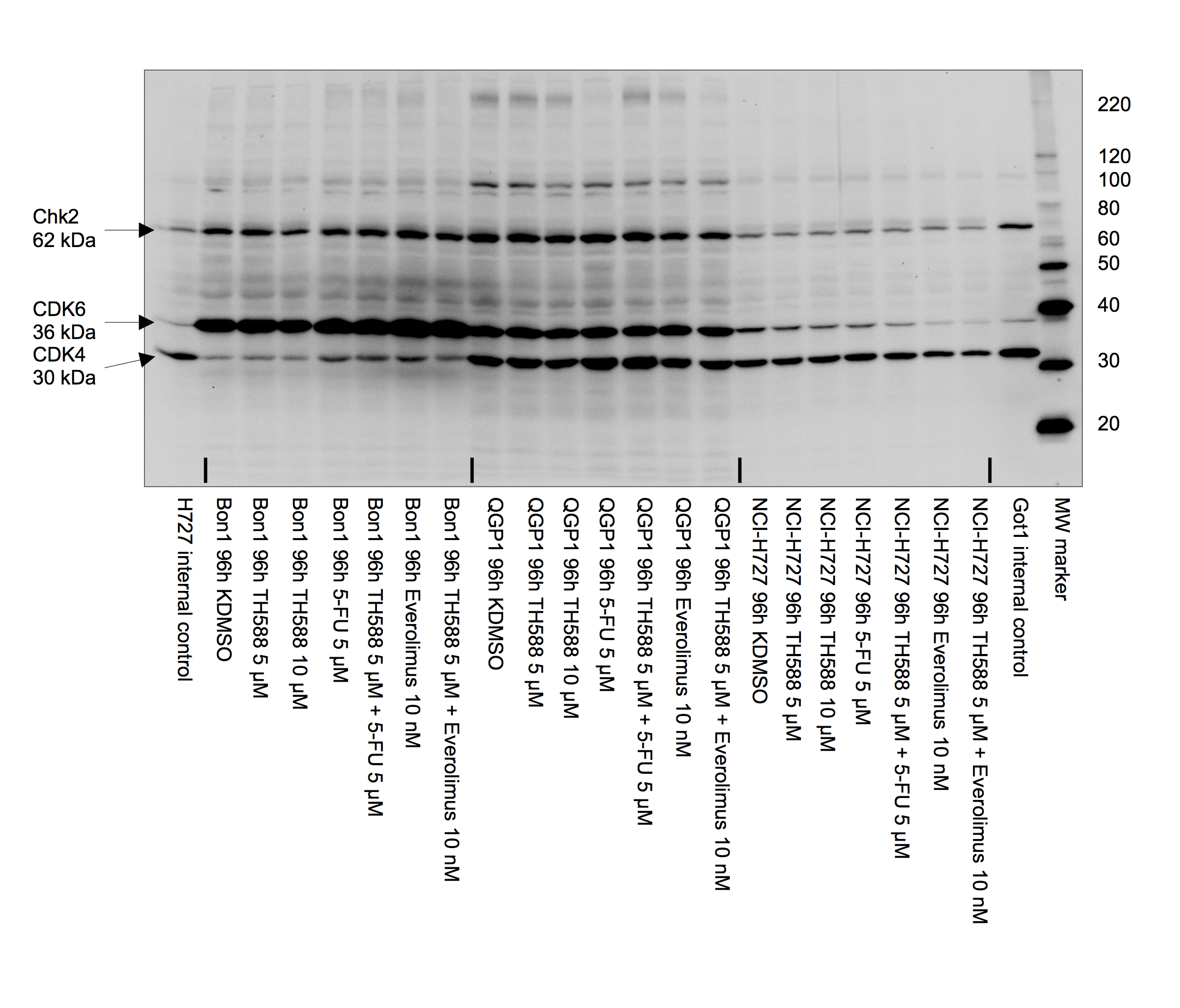

Supplement: S31 Fig — Expression of Chk2, CDK4 and CDK6 in neuroendocrine cell lines (BON1, H727 and QGP1) after 96 h of incubation with TH588 (5 μM or 10 μM) alone or in combination with 5FU (5 μM) or everolimus (10 nM). (TIF) [file pone.0178375.s031.tif]

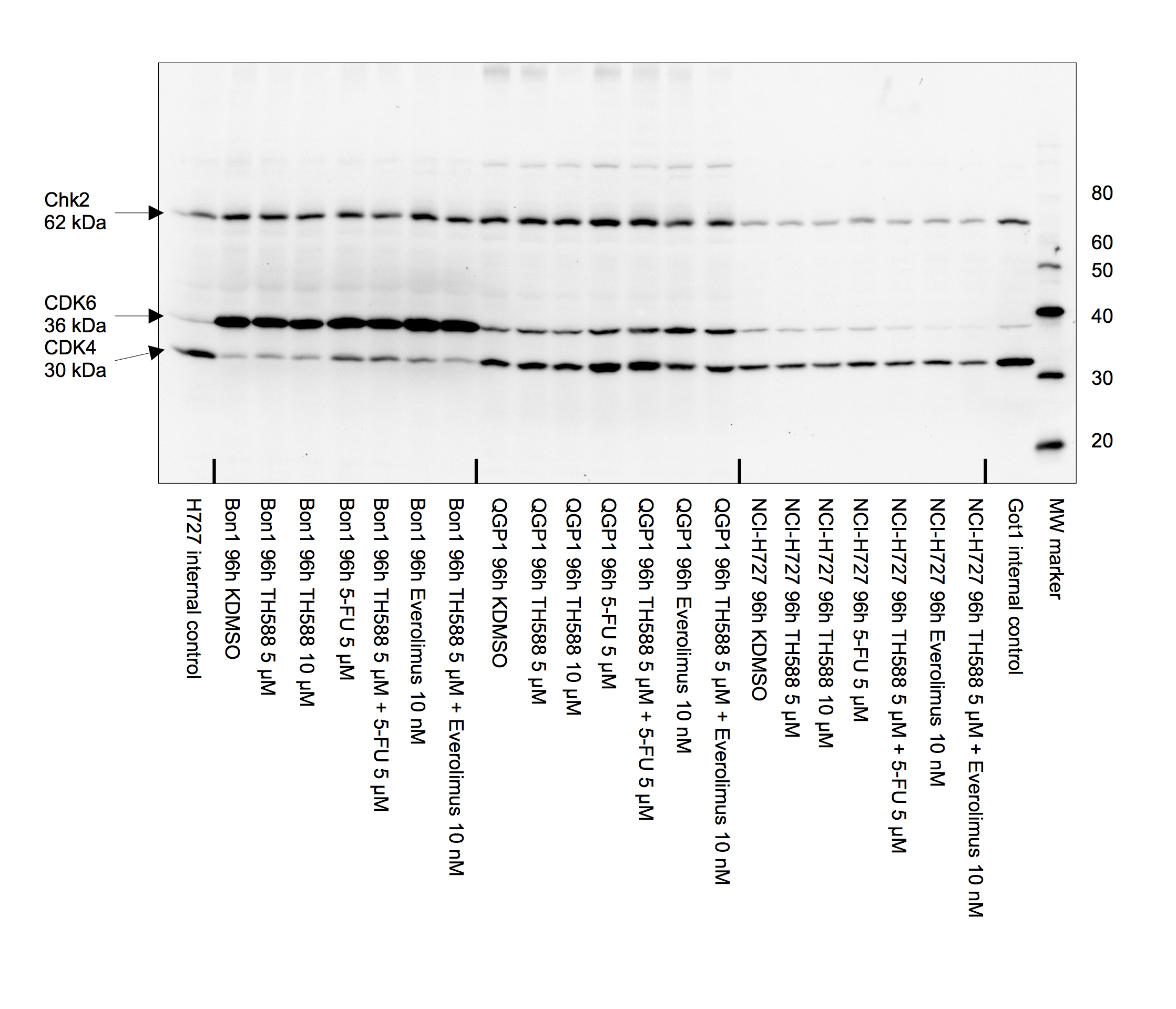

Supplement: S32 Fig — Expression of Chk2, CDK4 and CDK6 in neuroendocrine cell lines (BON1, H727 and QGP1) after 96 h of incubation with TH588 (5 μM or 10 μM) alone or in combination with 5FU (5 μM) or everolimus (10 nM). (TIF) [file pone.0178375.s032.tif]

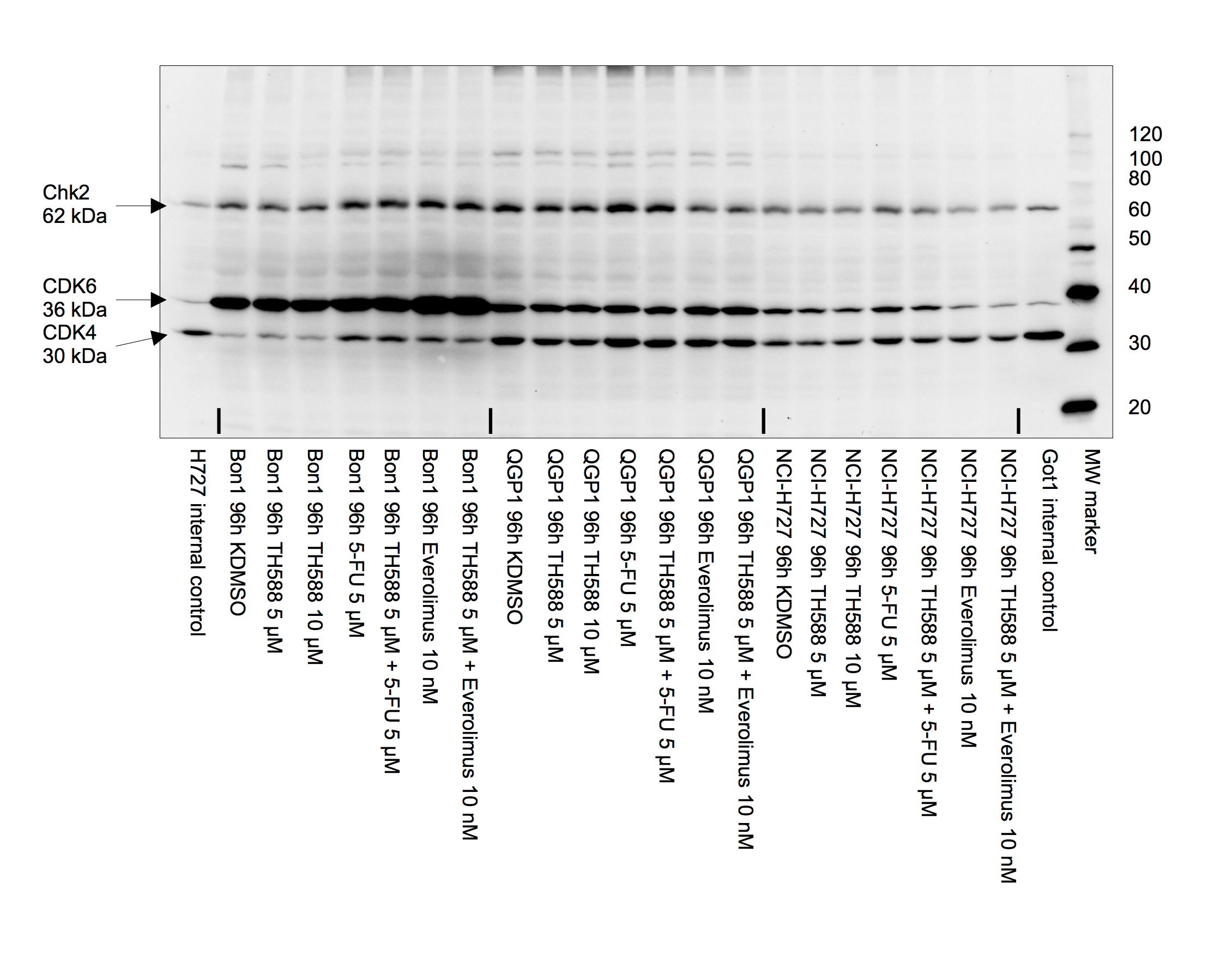

Supplement: S33 Fig — Expression of Chk2, CDK4 and CDK6 in neuroendocrine cell lines (BON1, H727 and QGP1) after 96 h of incubation with TH588 (5 μM or 10 μM) alone or in combination with 5FU (5 μM) or everolimus (10 nM). (TIF) [file pone.0178375.s033.tif]

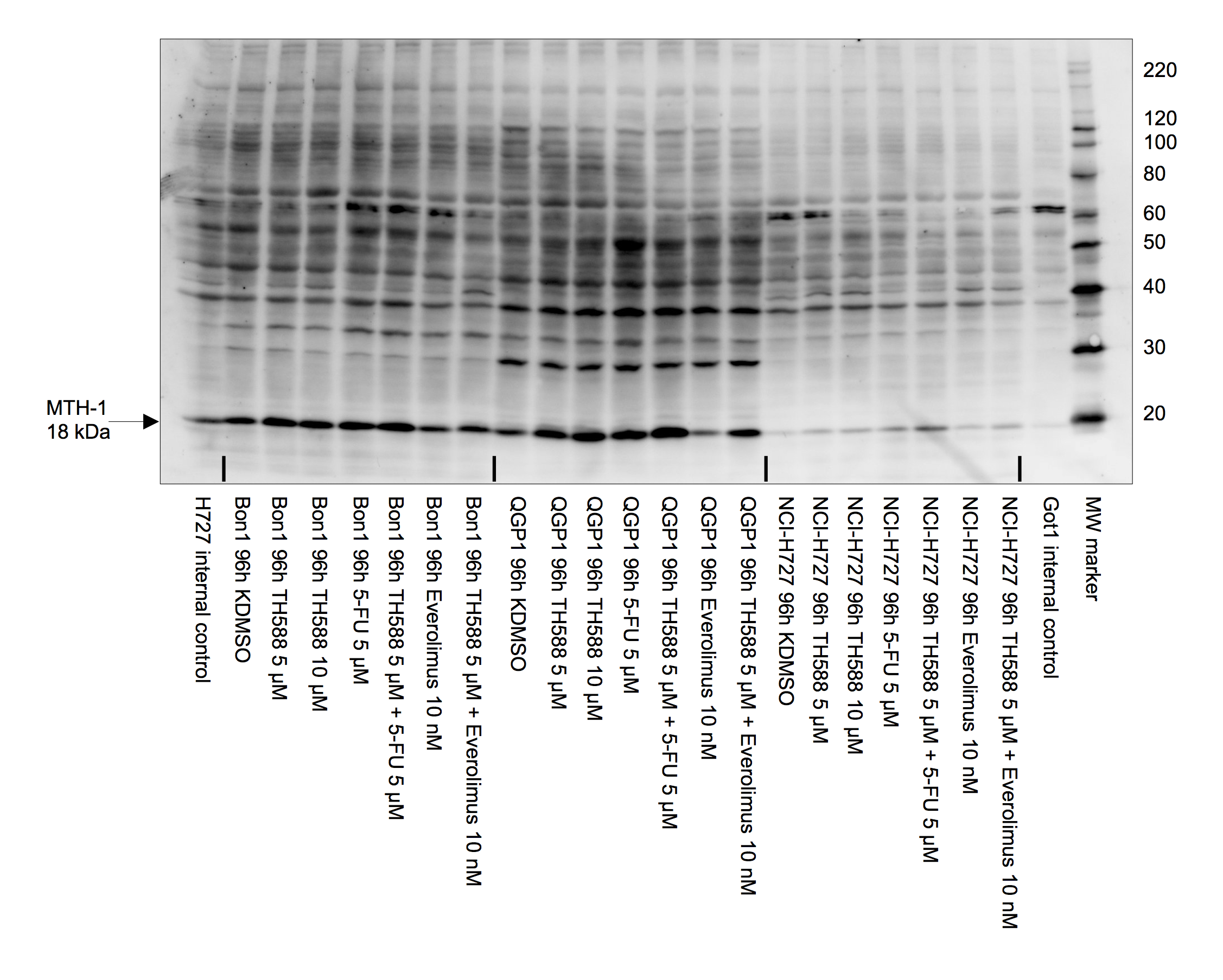

Supplement: S34 Fig — Expression of MTH1 in neuroendocrine cell lines (BON1, H727 and QGP1) after 96 h of incubation with TH588 (5 μM or 10 μM) alone or in combination with 5FU (5 μM) or everolimus (10 nM). (TIF) [file pone.0178375.s034.tif]

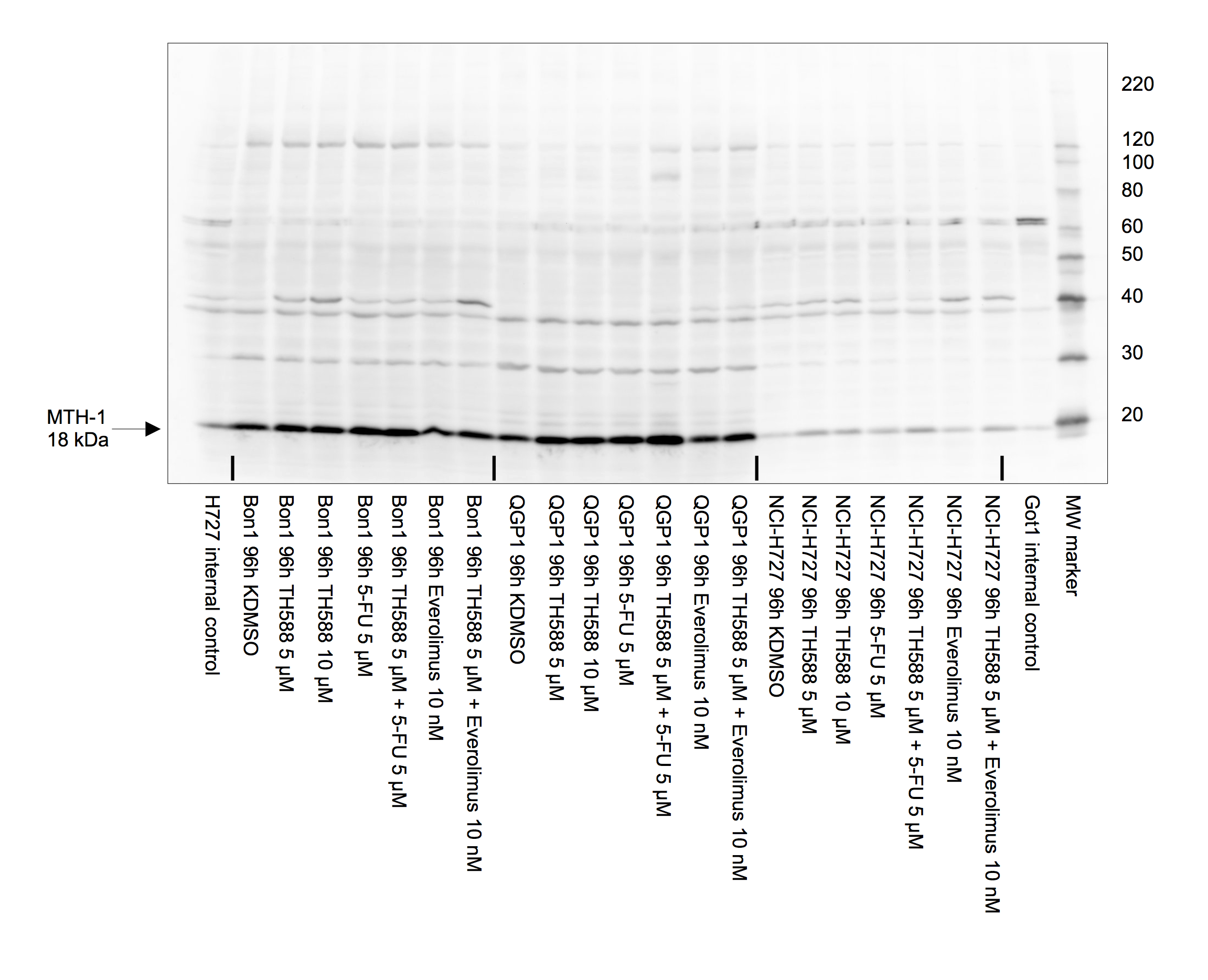

Supplement: S35 Fig — Expression of MTH1 in neuroendocrine cell lines (BON1, H727 and QGP1) after 96 h of incubation with TH588 (5 μM or 10 μM) alone or in combination with 5FU (5 μM) or everolimus (10 nM). (TIF) [file pone.0178375.s035.tif]

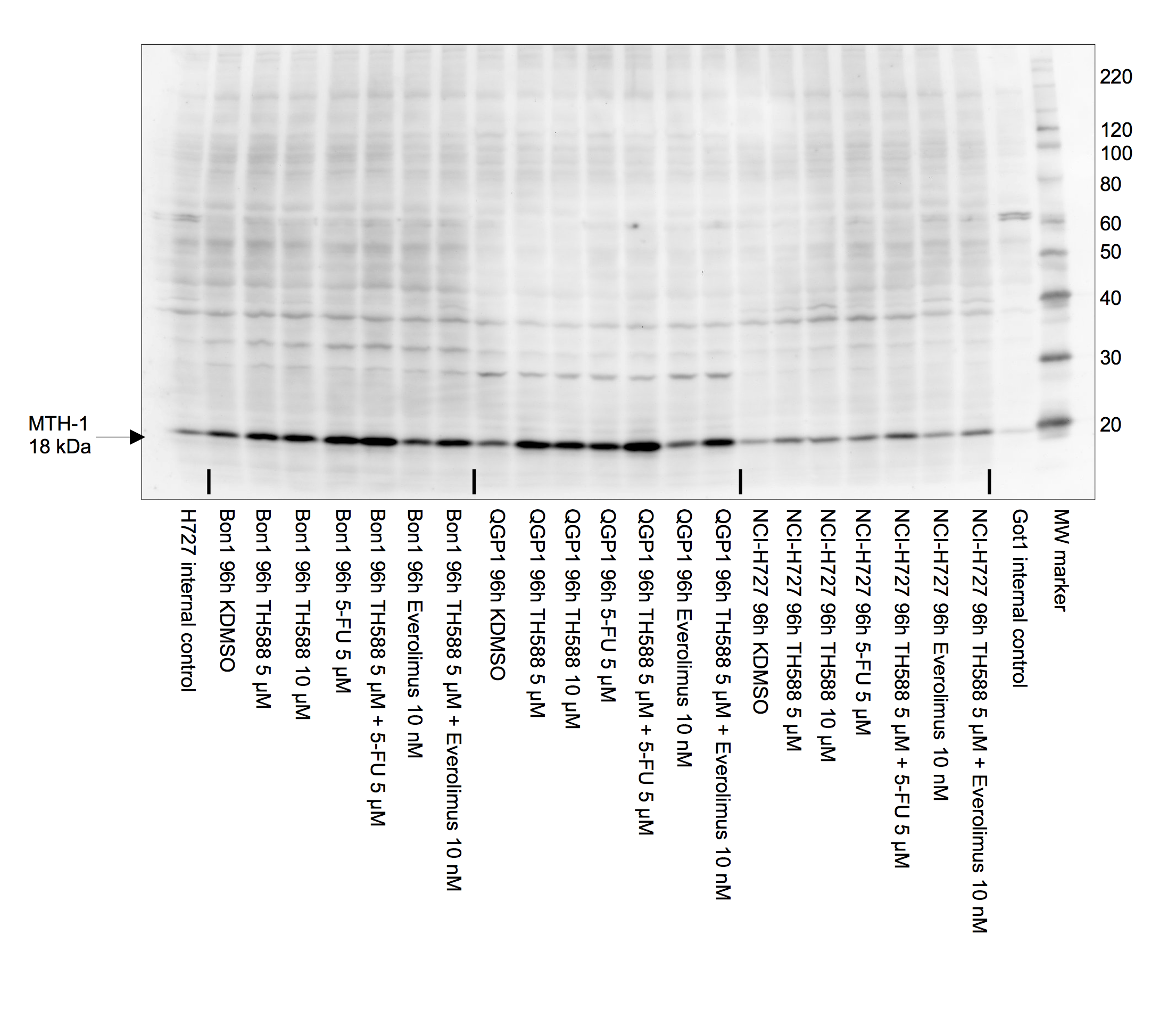

Supplement: S36 Fig — Expression of MTH1 in neuroendocrine cell lines (BON1, H727 and QGP1) after 96 h of incubation with TH588 (5 μM or 10 μM) alone or in combination with 5FU (5 μM) or everolimus (10 nM). (TIF) [file pone.0178375.s036.tif]

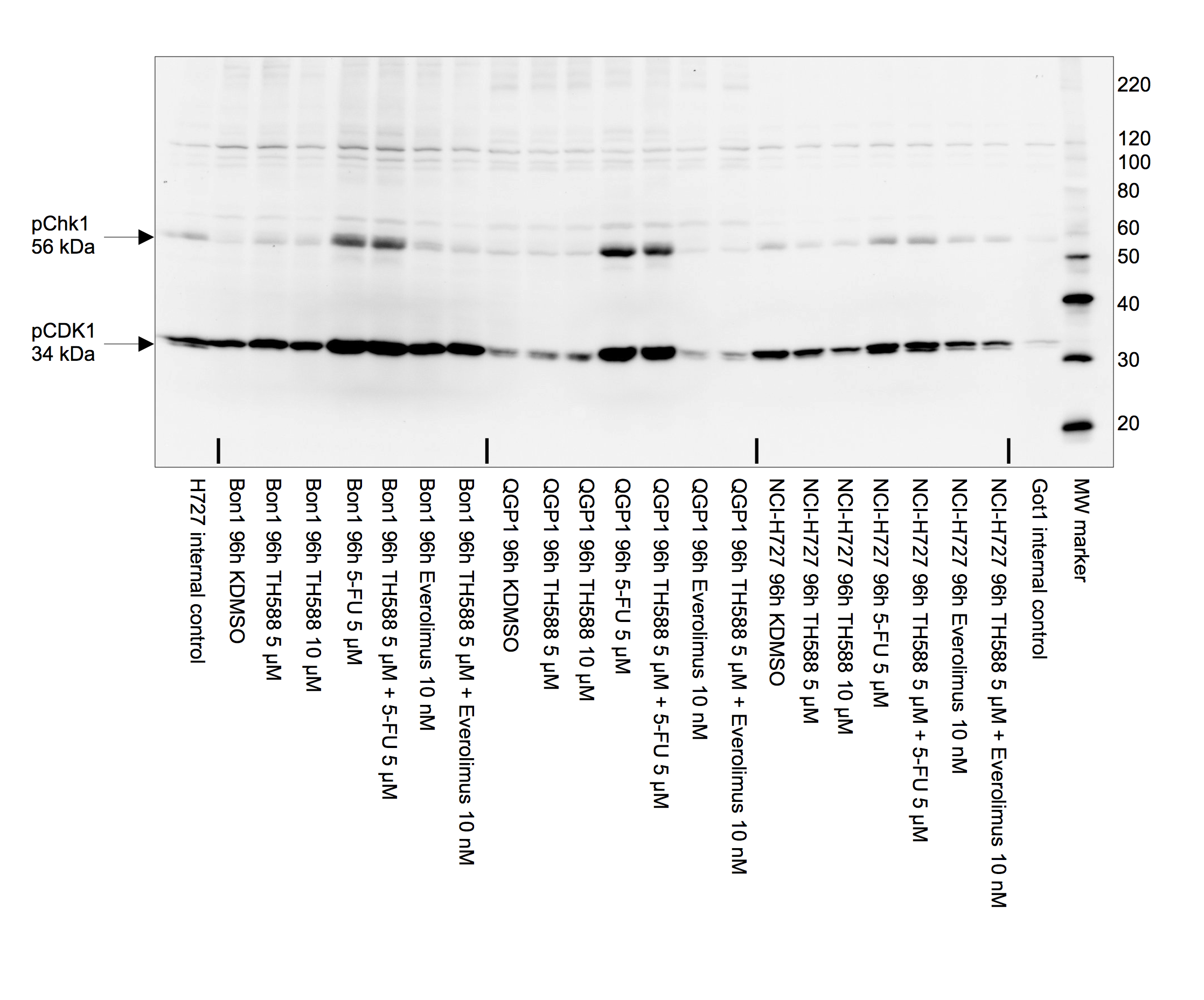

Supplement: S37 Fig — Expression of pChk1 and pCDK1 in neuroendocrine cell lines (BON1, H727 and QGP1) after 96 h of incubation with TH588 (5 μM or 10 μM) alone or in combination with 5FU (5 μM) or everolimus (10 nM). (TIF) [file pone.0178375.s037.tif]

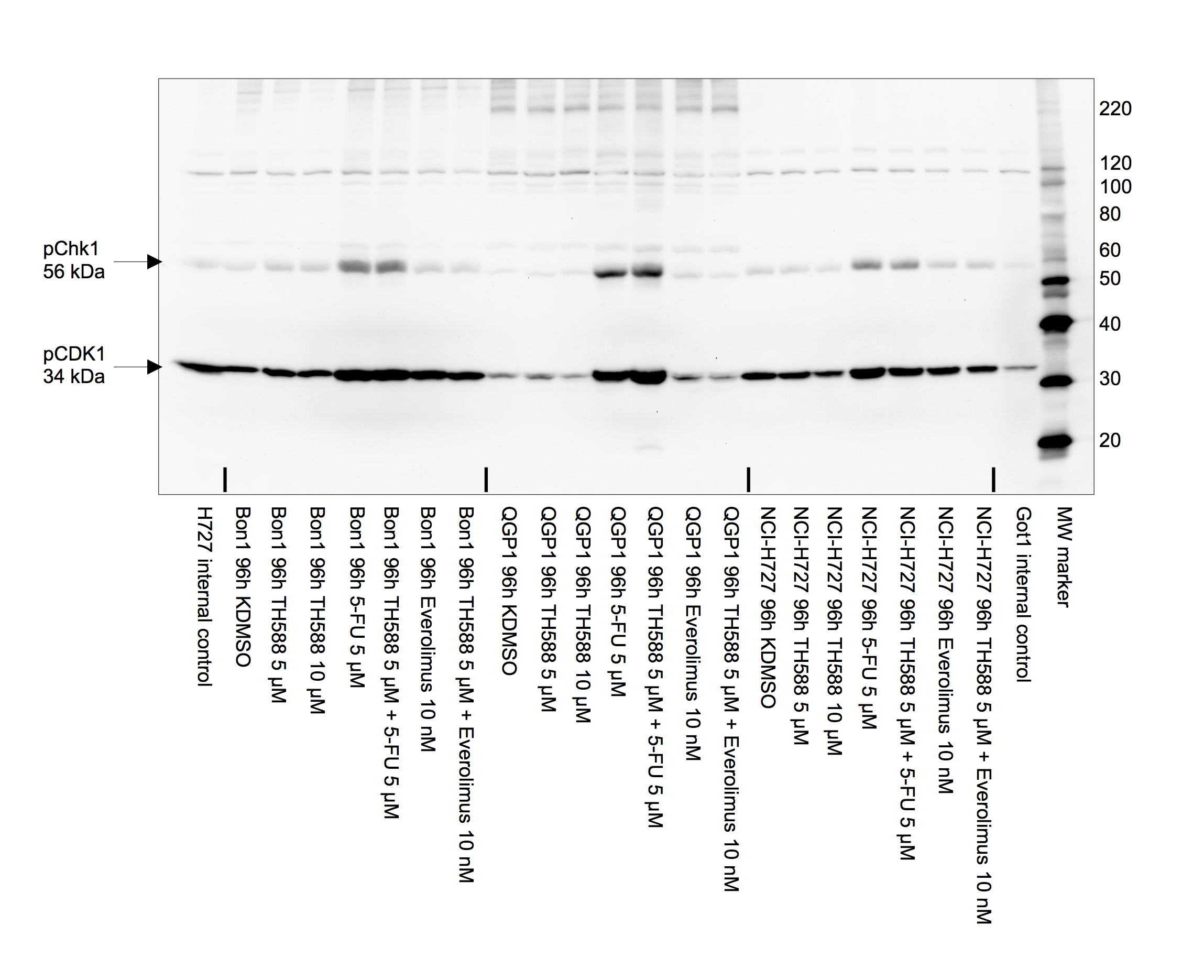

Supplement: S38 Fig — Expression of pChk1 and pCDK1 in neuroendocrine cell lines (BON1, H727 and QGP1) after 96 h of incubation with TH588 (5 μM or 10 μM) alone or in combination with 5FU (5 μM) or everolimus (10 nM). (TIF) [file pone.0178375.s038.tif]

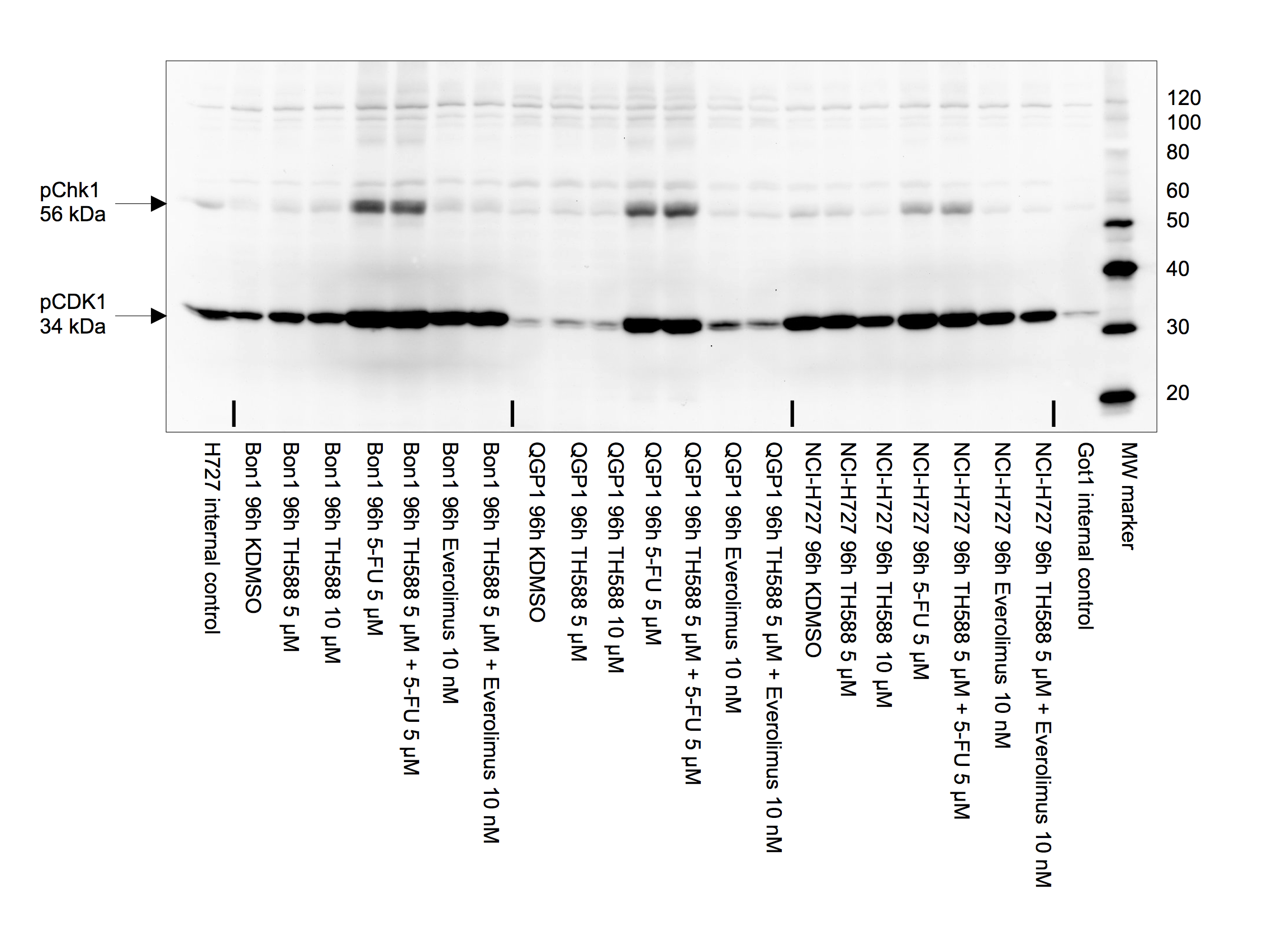

Supplement: S39 Fig — Expression of pChk1 and pCDK1 in neuroendocrine cell lines (BON1, H727 and QGP1) after 96 h of incubation with TH588 (5 μM or 10 μM) alone or in combination with 5FU (5 μM) or everolimus (10 nM). (TIF) [file pone.0178375.s039.tif]

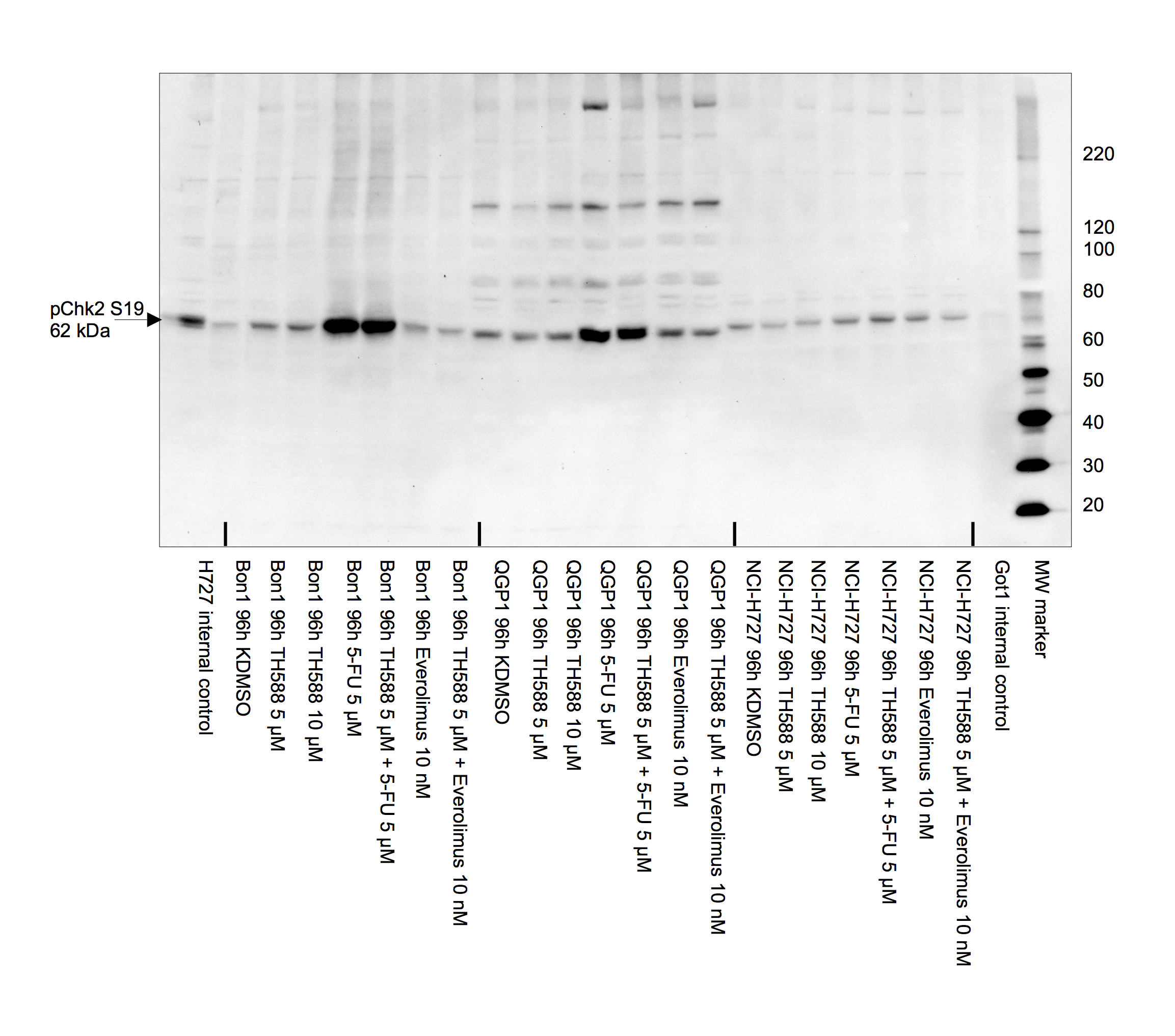

Supplement: S40 Fig — Expression of pChk2 in neuroendocrine cell lines (BON1, H727 and QGP1) after 96 h of incubation with TH588 (5 μM or 10 μM) alone or in combination with 5FU (5 μM) or everolimus (10 nM). (TIF) [file pone.0178375.s040.tif]

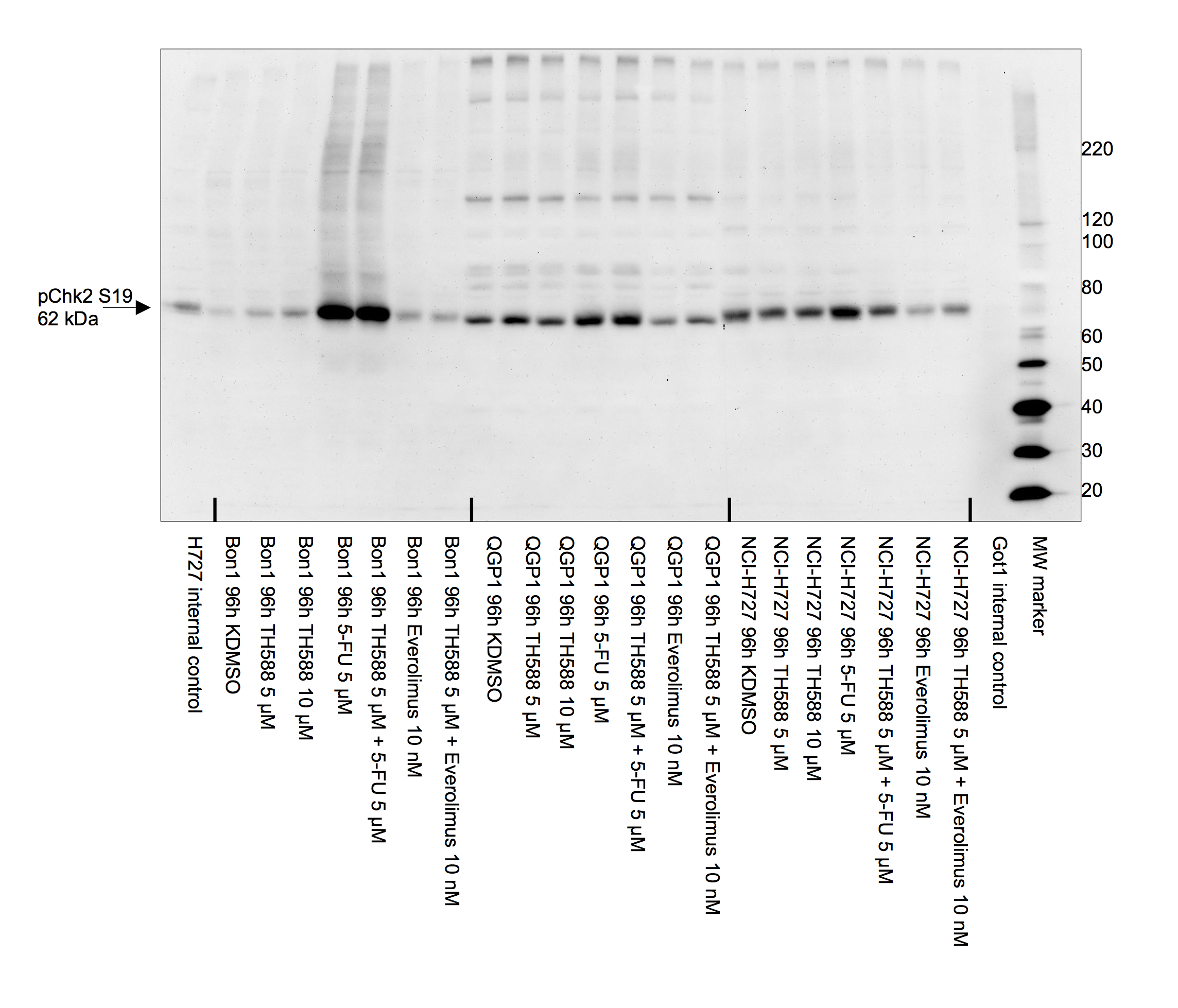

Supplement: S41 Fig — Expression of pChk2 in neuroendocrine cell lines (BON1, H727 and QGP1) after 96 h of incubation with TH588 (5 μM or 10 μM) alone or in combination with 5FU (5 μM) or everolimus (10 nM). (TIF) [file pone.0178375.s041.tif]

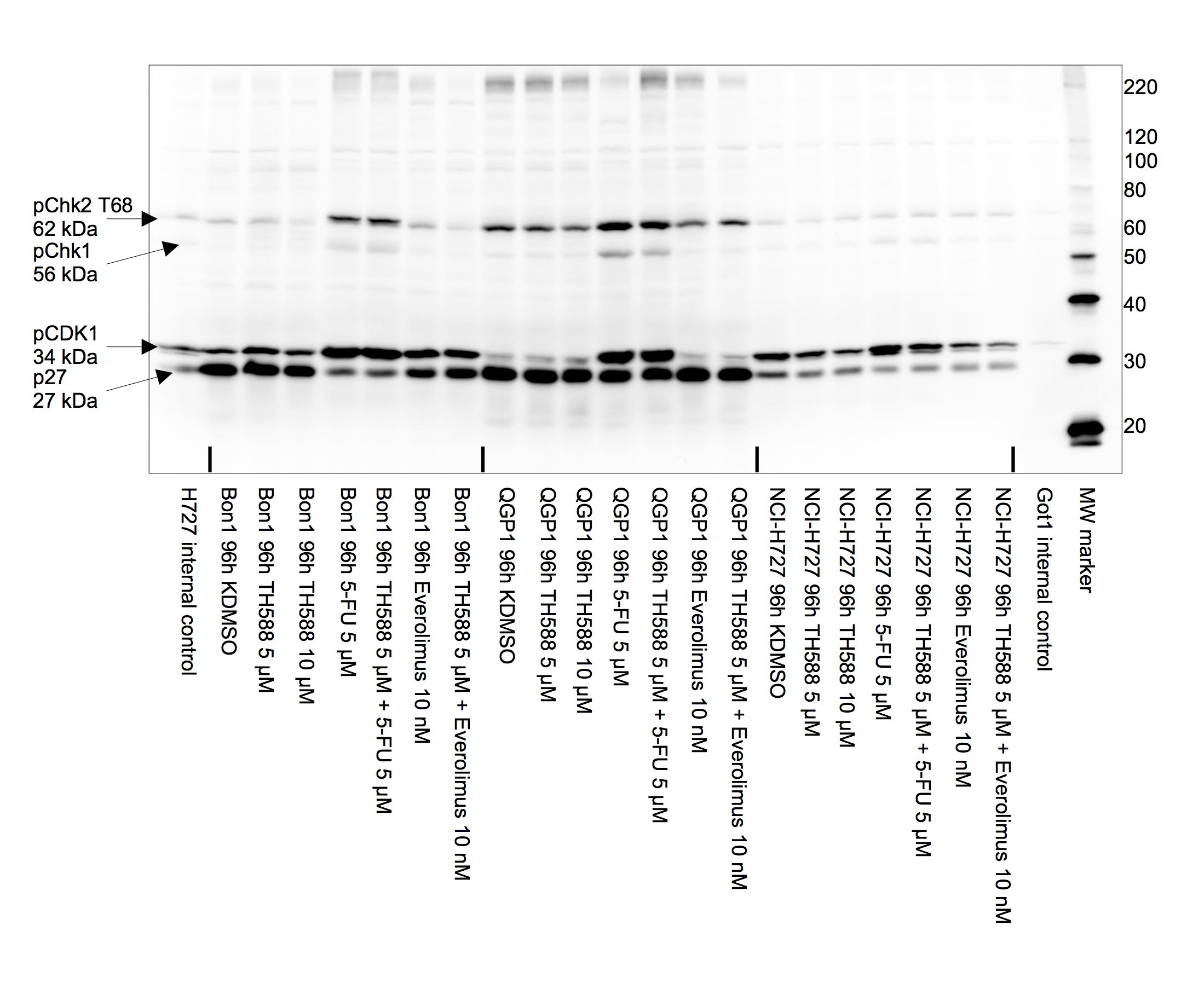

Supplement: S42 Fig — Expression of pChk1, pChk2, pCDK1 and p27 in neuroendocrine cell lines (BON1, H727 and QGP1) after 96 h of incubation with TH588 (5 μM or 10 μM) alone or in combination with 5FU (5 μM) or everolimus (10 nM). (TIF) [file pone.0178375.s042.tif]

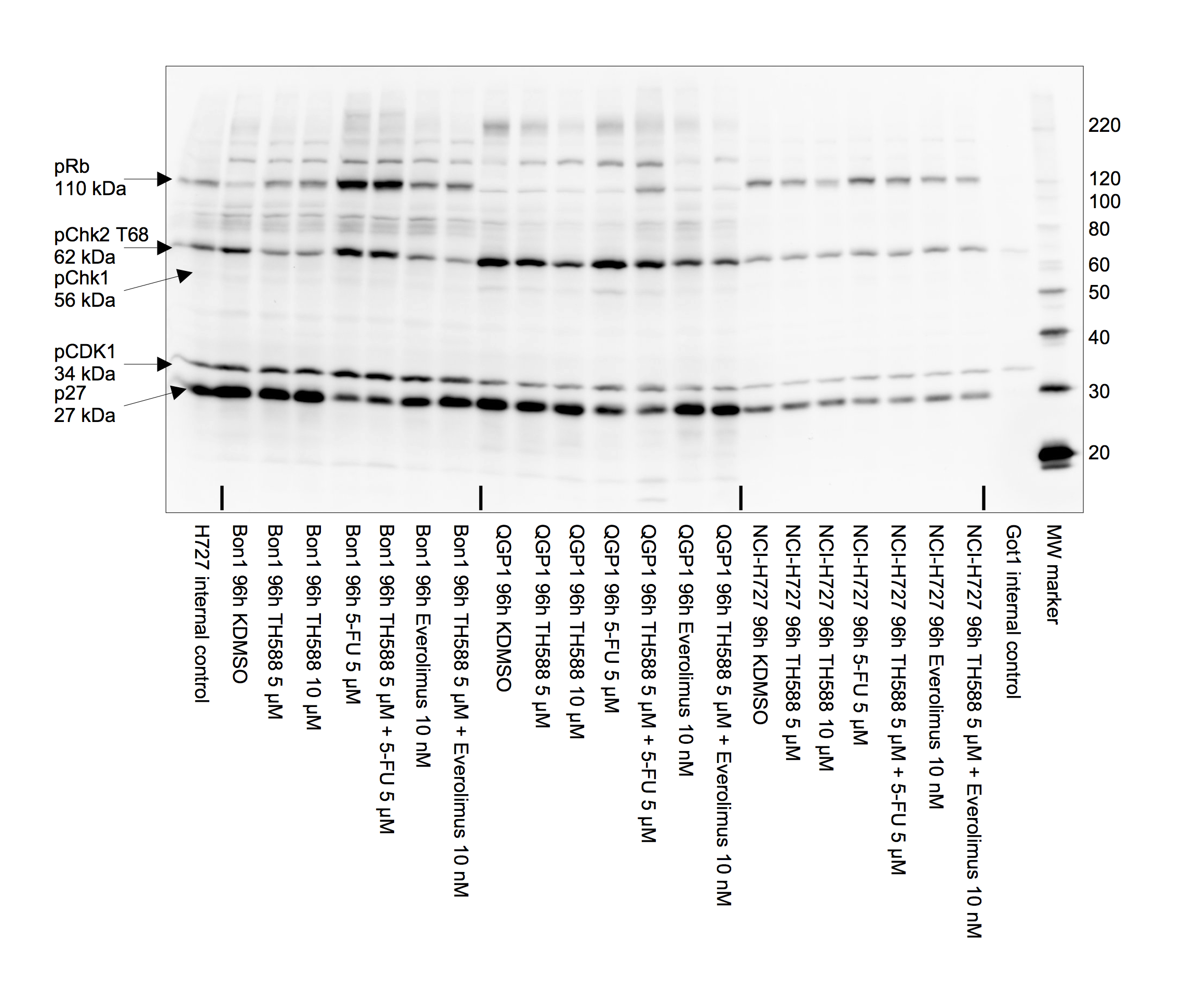

Supplement: S43 Fig — Expression of pRb, pChk1, pChk2, pCDK1 and p27 in neuroendocrine cell lines (BON1, H727 and QGP1) after 96 h of incubation with TH588 (5 μM or 10 μM) alone or in combination with 5FU (5 μM) or everolimus (10 nM). (TIF) [file pone.0178375.s043.tif]

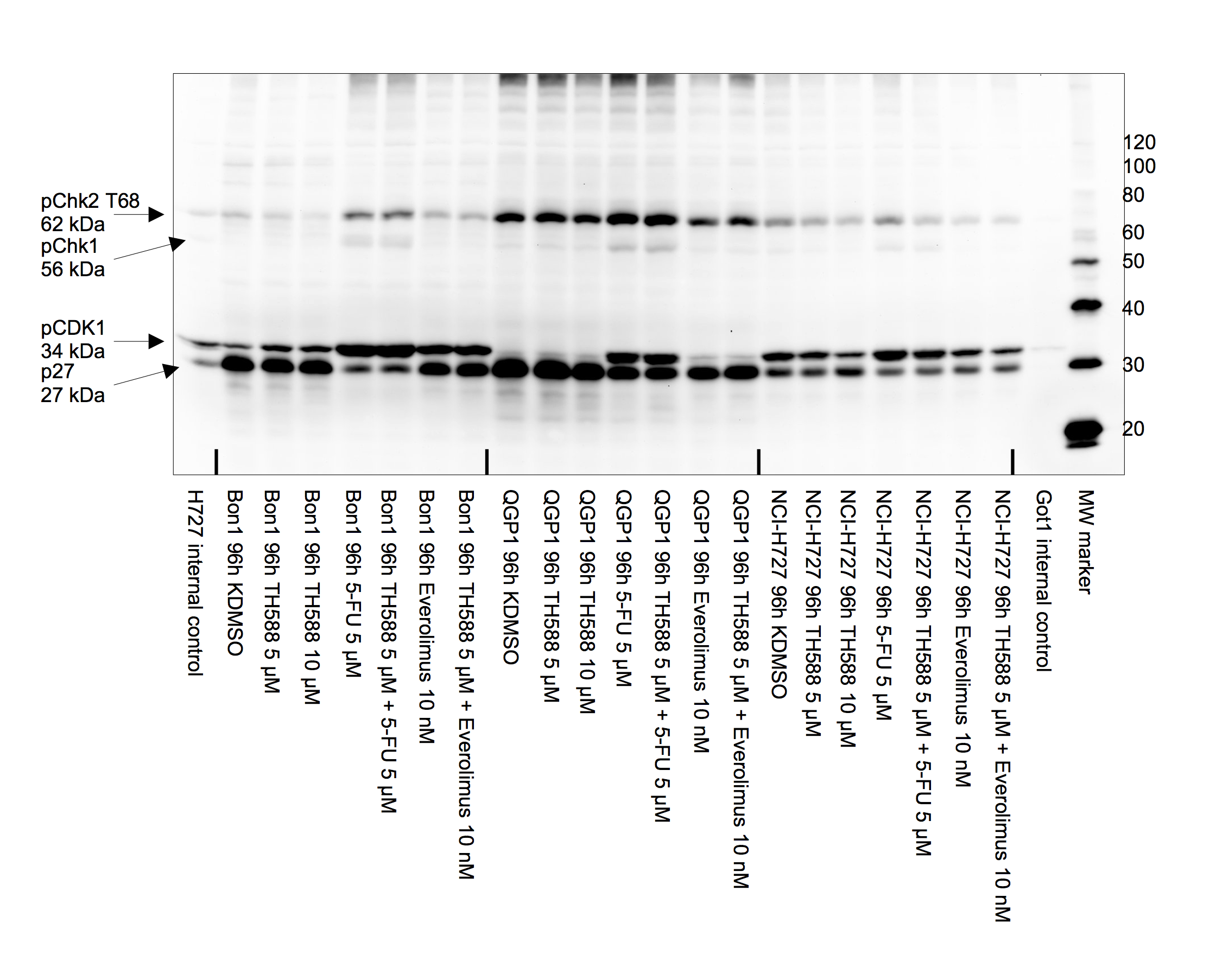

Supplement: S44 Fig — Expression of pChk1, pChk2, pCDK1 and p27 in neuroendocrine cell lines (BON1, H727 and QGP1) after 96 h of incubation with TH588 (5 μM or 10 μM) alone or in combination with 5FU (5 μM) or everolimus (10 nM). (TIF) [file pone.0178375.s044.tif]
